# Methylation of RBM39 by PRMT6 enhances resistance to Indisulam in non-small cell lung cancer by promoting alternative splicing of proto-oncogenes

**DOI:** 10.1371/journal.pbio.3002846

**Published:** 2025-06-04

**Authors:** Tongjia Zhang, Shujie Wang, Yue Zhou, Zitao Jiao, Kejia Lu, Xinyi Liu, Hui Li, Wei Jiang, Xiaowei Zhang

**Affiliations:** Department of Biochemistry and Molecular Biology, School of Basic Medical Sciences, Beijing Key Laboratory of Protein Posttranslational Modifications and Cell Function, Peking University Health Science Center, Beijing, China; UC Los Angeles: University of California Los Angeles, UNITED STATES OF AMERICA

## Abstract

Indisulam, a sulfonamide-based compound, is employed as a second-line therapy for NSCLC due to its anti-tumor activity. However, its clinical efficacy is hindered by acquired resistance, the molecular basis of which remains poorly understood. Here, we demonstrate that hypermethylation of RNA-binding protein 39 (RBM39), a specific target of Indisulam, is closely associated with Indisulam resistance. PRMT6 methylates RBM39 at R92. This methylation inhibits Indisulam-induced ubiquitination and proteasomal degradation of RBM39, increases RBM39 protein levels, promotes alternative splicing and expression of proto-oncogenes, and ultimately leads to malignant proliferation and metastasis of NSCLC cells and tumor growth in xenograft mouse models. Inhibiting PRMT6 with MS023 or mutating the RBM39 methylation site enhances Indisulam sensitivity in NSCLC and significantly improves its anti-tumor efficacy. Our findings identify methylated RBM39 as a key biomarker of Indisulam resistance and suggest a potential therapeutic strategy for NSCLC.

## Abstract 2

The RNA-binding protein RBM39 has been previously shown to be a target of the anti-cancer agent Indisulam. This study shows that RBM39 is methylated by PRMT6, which determines the resistance to Indisulam in lung cancer models due to aberrant splicing events.

## Introduction

Lung cancer remains the leading cause of cancer-related death worldwide [1]. Non-small cell lung cancer (NSCLC) is the most common subtype, accounting for approximately 85% of all lung cancer, including lung adenocarcinoma (LUAD), lung squamous cell carcinoma (LUSC), and large cell lung carcinoma (LCLC) [2,3]. Over 60% of NSCLC patients are diagnosed with advanced stage, often with metastatic disease, poor prognosis, and a 5-year overall survival rate below 15% [1,4,5]. In the past 2 decades, substantial progress in understanding the molecular biology of oncogene-driven tumors has led to improved treatment strategies for these patients [6]. But until now, for NSCLC, surgery is the only curative option for patients in the early stage. The majority of patients present with advanced disease, for which treatment is palliative [7]. First-line chemotherapy has limited impact on clinical outcome, and while second-line treatment can prolong survival, its response rate remains low at 7%–9% [7–9]. There is an urgent clinical need to develop effective second-line therapies to improve patient response and survival.

Indisulam is a clinically developed sulfonamide used as a second-line treatment for solid tumors. In human cancer cell lines, Indisulam induces G1 phase arrest followed by cell death [10,11]. In multiple phase I and II trials in advanced NSCLC patients, Indisulam monotherapy showed limited clinical benefit, indicating substantial resistance [7,12–18]. Recent studies identified RNA-binding motif protein 39 (RBM39) as a key target of Indisulam. The compound functions as a molecular glue, promoting interaction between RBM39 and the DCAF15-associated E3 ubiquitin ligase complex, leading to its selective degradation [19,20]. RBM39 degradation causes aberrant splicing and altered gene expression, which suppresses tumor progression in various preclinical models. These potent anti-cancer effects contrast sharply with Indisulam’s limited efficacy in NSCLC clinical trials. Therefore, exploring the function and regulation to identify predictive markers for Indisulam-based anti-cancer therapy.

RBM39 is an essential serine/arginine (SR)-rich RNA-binding protein (RBP) with dual functions of transcriptional coactivation and RNA splicing. Consequently, it contributes to tumor progression by regulating transcription and alternative splicing (AS) of tumor-related genes. Its expression is up-regulated in several cancers, including lung, breast, and colorectal cancers [14–16]. As a coactivator, RBM39 enhances the activity of transcription factors such as ERα, ERβ, AP-1, and NF-κB [21–24]. However, its role in RNA splicing has drawn increasing research attention. RBM39 interacts with vital splicing factors U2AF65 and PUF60 to mediate pre-mRNA AS and promote oncogene expression [25]. RBM39 depletion causes splicing defects, the most common forms of splicing defects being cassette exon (exon skipping or inclusion) and intron retention. These splicing abnormalities severely affect the metabolism, cell cycle, and stress response of cancer cells [19,25,26]. Given its function, RBM39 may serve as a prognostic marker and a tool to monitor drug resistance in NSCLC patients [27]. Unfortunately, the post-translational modification (PTM) of RBM39 and its impact in drug resistance remain poorly understood. RBM39 undergoes ubiquitination and phosphorylation, which regulate its degradation and transcription coactivation activity, respectively [28]. As an SR-rich protein, it remains to be determined whether RBM39 is methylated by protein arginine methyltransferases (PRMTs) and how this affects NSCLC resistance to Indisulam.

Protein arginine methylation refers to the transfer of a methyl group from the S-adenosyl-L-methionine (SAM) donor molecule to the arginine residue guanidine nitrogen atom in the protein substrate under the catalysis of PRMT [29]. In mammals, the PRMT family is classified into three types based on catalytic activity: type I enzymes (PRMT1–4, PRMT6, and PRMT8); type II enzymes (PRMT5 and PRMT9); and type III enzymes (PRMT7). Type III enzymes exclusively generate ω-NG-monomethyl arginine (MMA, Rme1), an intermediate between ω-NG, NG-asymmetric dimethylarginine (ADMA, Rme2a) and ω-NG, NG-symmetric dimethylarginine (SDMA, Rme2s) catalyzed by type I and type II enzymes, respectively [30]. Many PRMTs are abnormally expressed in cancer cells, promoting cancer stem cell (CSC) generation, epithelial–mesenchymal transition (EMT), and tumor progression. Hence, aberrant arginine methylation of some proteins contributes to drug resistance by enabling the formation of drug-tolerant cancer cells [31]. PRMT6, a type I enzyme, generates ADMA and regulates gene expression epigenetically, along with AS, cell proliferation, and drug resistance [32]. These characteristics make PRMT6 a promising target for cancer therapy.

In this study, we demonstrate that PRMT6 methylates RBM39 at arginine 92 (R92). This methylation stabilizes RBM39 protein and promotes the splicing and expression of proto-oncogenes, contributing to Indisulam resistance in NSCLC cells. The PRMT6 inhibitor MS023 inhibits RBM39 methylation, thereby reducing Indisulam resistance and enhancing Indisulam sensitivity. This provides an effective regimen and specific drug target for second-line drug treatment of NSCLC.

## Results

### RBM39 promotes Indisulam resistance in NSCLC cells

**Fig 1:**
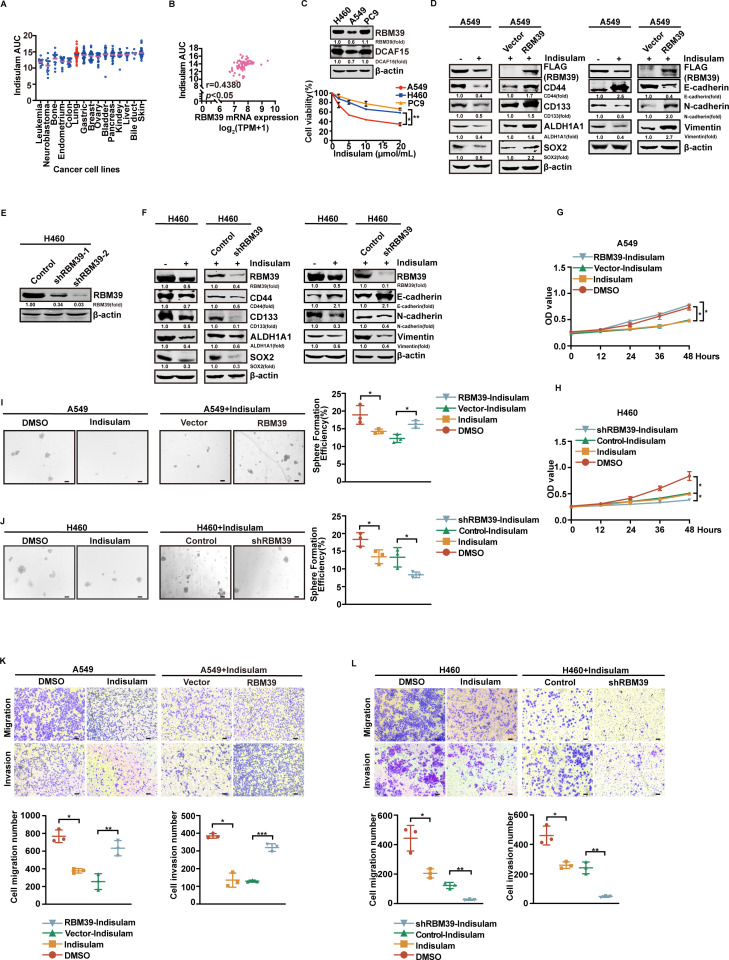
RBM39 promotes Indisulam resistance in NSCLC cells. **(A–C**) RBM39 expression is associated with Indisulam resistance in NSCLC cell lines. (**A**) Sensitivity to Indisulam in various cancer cell lines (*n* = 15). (**B**) The correlation between RBM39 expression and Indisulam sensitivity in NSCLC cells lines from CTRP and CCLE public database (*n* = 89). (**C**) Western blotting was assessed the protein levels of RBM39 and DCAF15. Western blot and MTT assays validated that NSCLC cells with high RBM39 expression were less sensitive to Indisulam. In NSCLC, sensitivity to Indisulam correlates with RBM39 expression but not with DCAF15. (**D–J**) RBM39 reverses Indisulam-induced inhibition of stemness and proliferation in NSCLC cells. (**D**) RBM39 overexpression reversed CSC markers and EMT marker levels inhibited by Indisulam. Protein levels were analyzed by western blotting in A549 cells transfected with empty vector or RBM39, with or without Indisulam treatment. (**E**) Western blotting showed the knockdown effect of RBM39 by shRNA in H460 cells. (**F**) RBM39 knockdown enhanced Indisulam-induced suppression of CSC and EMT marker expression. Western blotting was used to assess the protein levels of CSC and EMT markers in H460 cells treated with or without Indisulam and RBM39 knockdown or not. (**G** and **H**) Cell proliferation was evaluated using MTT assay (*n* = 3). (**G**) RBM39 overexpression reversed the Indisulam-mediated inhibition of A549 cell proliferation. (**H**) Compared with the control, RBM39 knockdown combined with Indisulam further reduced proliferation. (**I** and **J**) Sphere formation assays were performed in the indicated cell lines. Bars = 200 μm. RBM39 overexpression increased the number of spheres in A549 cells treated with Indisulam (**I**). RBM39 knockdown reduced the number of spheres in Indisulam-treated H460 cells (**J**). **(K** and **L**) Transwell assays were used to assess cell migration and invasion. Bars = 200 μm. RBM39 overexpression reversed Indisulam-induced suppression of migration and invasion in A549 cells (**K**). RBM39 knockdown enhanced Indisulam-induced inhibition of migration and invasion in H460 cells (**L**). The underlying data for Fig 1A–1C and 1G–1L can be found in S3 Data. Data calculates the mean ± SD (*n* = 3). *p *< *0.05, **p *< *0.01, ***p *< *0.001. Statistical analysis was calculated using a two-tailed Student *t* test. NSCLC, non-small cell lung cancer; CSC, cancer stem cell; EMT, epithelial–mesenchymal transition; MTT, 3-(4,5-Dimethylthiazol-2-yl)-2,5-diphenyltetrazolium bromide assay; CCLE, Cancer Cell Line Encyclopedia; CTRP, Cancer Therapeutics Response Portal; RBM39, RNA-binding protein 39.

Recent studies have proven that Indisulam selectively induces proteasomal degradation of RBM39 via DCAF15-E3-ubiquitin ligase complex [33]. Although Indisulam induces tumor-specific apoptosis, it remains ineffective as a monotherapy for second-line NSCLC treatment [7]. In Cancer Therapeutics Response Portal (CTRP) analysis of cell lines, the area under the dose–response curve (AUC) of Indisulam for lung cancer showed lower sensitivity compared with cell lines from leukemia, neuroblastoma, bone, endometrium, and colon cancers (Fig 1A). Additionally, we discovered a negative correlation between RBM39 mRNA expression and Indisulam sensitivity (Fig 1B). Western blotting and MTT, 3-(4,5-Dimethylthiazol-2-yl)-2,5-diphenyltetrazolium bromide assay (MTT) validated the results: Indisulam sensitivity negatively correlates with RBM39 levels (Fig 1C). It has been reported that ubiquitin E3 ligase DCAF15 mediates the sensitivity of gastric cancer cells to Indisulam [34]. Thus, we also detected DCAF15 protein level in the three NSCLC lines and found that the DCAF15 protein level in A549 cells was lower than that in H460 and PC9 cells, which was not consistent with the RBM39 protein level. There was no clear association with reduced cell survival induced by Indisulam (Fig 1C). Our data suggest that DCAF15 expression may not be the key factor determining cell sensitivity to Indisulam in NSCLC. Previous studies have reported that Indisulam promotes RBM39 ubiquitination via the DCAF15 complex [9]. To assess the role of RBM39 expression in Indisulam sensitivity, we generated stable A549 cells overexpressing RBM39 (low endogenous expression) and H460 cells with RBM39 knockdown via shRNA (high endogenous expression). Among the shRNAs tested, shRBM39-2 showed the most effective knockdown and was used in all subsequent experiments (Fig 1E). CSC-like characteristics and EMT are two paramount attributes that drive the cascade of tumor development and metastasis. These markers were assessed using western blotting. As shown in Fig 1D, Indisulam treatment reduced the expression of CSC markers (CD44, CD133, ALDH1A1, SOX2) and EMT markers (N-cadherin, vimentin), while increasing E-cadherin expression. Compared to Indisulam alone, the RBM39 overexpression attenuated the drug’s suppressive effects, resulting in elevated CSC markers, N-cadherin, and vimentin, and reduced E-cadherin levels. We found that RBM39 promotes cell proliferation in the absence of Indisulam, whereas its overexpression reverses the inhibitory effect of Indisulam on proliferation when Indisulam is present (Figs 1G and S1A). RBM39 overexpression reversed the inhibitory effects of Indisulam on CSC-like properties (spheroid formation, colony formation, proportion of CD44+ CD133+ cells) in A549 cells (Figs 1I, S1C and S1E). In contrast, RBM39 knockdown enhanced the suppressive effect of Indisulam, leading to a further reduction in the expression of CSC markers, N-cadherin, and vimentin compared to Indisulam alone. Conversely, E-cadherin expression was increased (Fig 1F). RBM39 knockdown inhibits cell proliferation, and its combination with Indisulam further enhances this effect (Figs 1H and S1B). Similarly, RBM39 knockdown significantly enhanced the inhibitory effects of Indisulam on CSC-like properties and colony formation in H460 cells, compared to Indisulam treatment alone (Figs 1J, S1D and S1F). Additionally, RBM39 overexpression significantly promoted the migration and invasion of A549 cells under Indisulam treatment compared to using Indisulam alone (Figs 1K and S1G). In contrast, RBM39 knockdown in combination with Indisulam significantly reduced the migration and invasion of H460 cells (Figs 1L and S1H). We further validated the effect of Indisulam on NSCLC using RBM39 knockout cells. Compared with Indisulam treatment alone, the combination of RBM39-KO and Indisulam did not significantly enhance the suppression of tumor stemness, proliferation, migration, or invasion, further supporting the role of RBM39 as the target protein of Indisulam (S2 Fig). These findings show that high expression of RBM39 attenuates the inhibitory effect of Indisulam on stemness and EMT characteristics of NSCLC cells and enhances the drug resistance of NSCLC cells.

### RBM39 promotes NSCLC cell stemness and EMT

**Fig 2:**
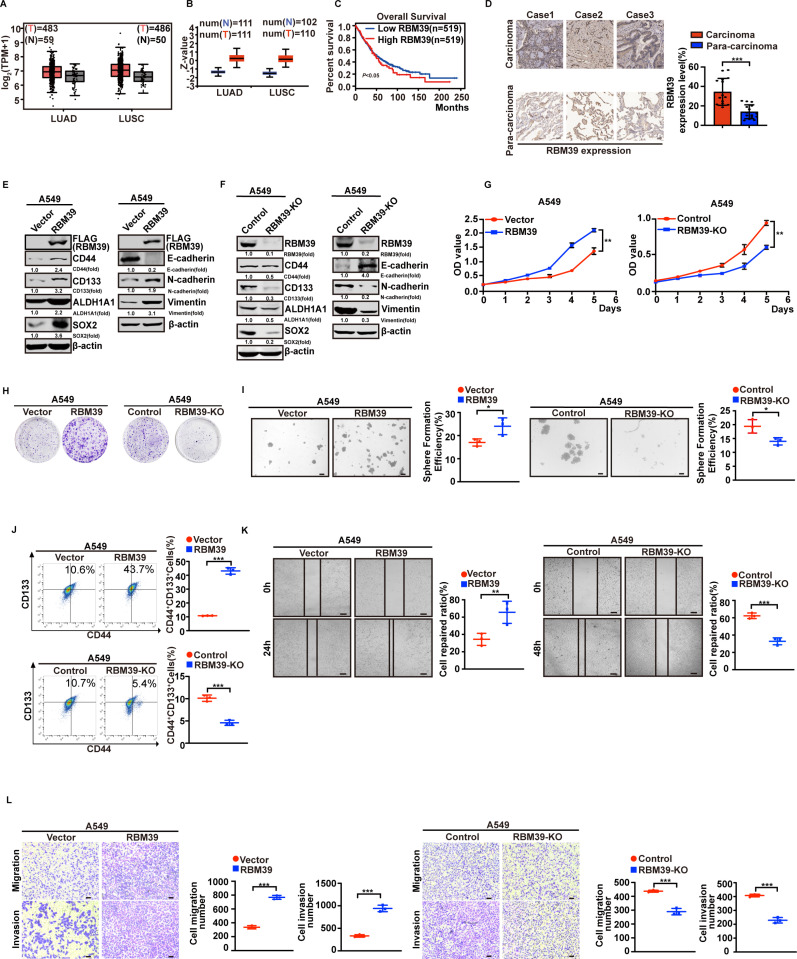
RBM39 promotes NSCLC cell stemness and EMT. (**A–D**) RBM39 is highly expressed in NSCLC. (**A** and **B**) RBM39 mRNA (**A**) and protein (**B**) levels are highly expressed in NSCLC through Gene Expression Profiling Interactive Analysis (GEPIA) and the University of Alabama at Birmingham Cancer Data Analysis Portal (UALCAN). (**C**) Survival analysis was conducted by dividing patients into two groups based on RBM39 expression levels. The high and low RBM39 expression groups were defined using the median expression value as the cutoff (low RBM39 = 519, high RBM39 = 519). These results suggest that high RBM39 expression is associated with a poorer prognosis in NSCLC. (**D**) Immunohistochemistry showed higher RBM39 expression in NSCLC tissues compared with para-carcinoma (*n* = 15). Bars = 50 μm. (**E**) RBM39 overexpression increases the expression of NSCLC-CSC surface markers and EMT-related markers. The protein levels of these markers in the cell lysate were measured by western blotting in A549 cells transfected with pLVX-IRES-Puro-RBM39 or pLVX-IRES-Puro-Vector (as a control). β-actin was used as a control. **F** RBM39 knockout reduces the expression of NSCLC-CSC surface markers and EMT-related markers. A549 cells with or without RBM39 knockout were analyzed by western blotting to detect the protein levels. (**G** and **H**) RBM39 promotes cell proliferation. (**G**) MTT (*n* = 3) was used to assess cell proliferation viability in A549 cells with RBM39 overexpression or knockout. (**H**) Colony formation ability of A549 cells with RBM39 overexpression or knockout was assessed using a colony formation assay. (**I**) Sphere formation assays (*n* = 3) were performed in indicated cell lines to detect the sphere-forming ability. RBM39 overexpression improves sphere-forming ability, and RBM39 knockout reduces sphere-forming ability. Representative images (**I**, left) and sphere number analysis (**I**, right) were shown. Bars = 200 μm. (**J**) The proportion of CD44^+^ CD133^+^ cells in each group was analyzed using flow cytometry. RBM39 overexpression enhances the number of CD133 and CD44 positive cells. RBM39 knockout reduces the number of CD133 and CD44 positive cells. Representative dot plots (**J**, left) and percentage of CD44^+^ CD133^+^ cells (**J**, right) were shown. (**K**) The effect of RBM39 on A549 motile ability was measured by scratch wound healing assay. RBM39 overexpression promotes A549 cell motility. RBM39 knockout inhibits A549 cell motility. Bars = 100 μm. (**L**) Transwell assays were performed to test the migratory and invasive potential of the A549 cells. RBM39 overexpression promotes A549 cell migratory and invasive, RBM39 knockout blocks A549 cell migratory and invasive. Bars = 200 μm. Data calculates the mean ± SD (*n* = 3). *p *< *0.05, **p *< *0.01, ***p *< *0.001. Statistical analysis was calculated using a two-tailed Student *t* test. The underlying data for Fig 2C, 2D, 2G, and 2I–2L can be found in S3 Data. NSCLC, non-small cell lung cancer; CSC, cancer stem cell; EMT, epithelial–mesenchymal transition; MTT, 3-(4,5-Dimethylthiazol-2-yl)-2,5-diphenyltetrazolium bromide assay; RBM39, RNA-binding protein 39.

Through database analysis, we found that RBM39 is significantly expressed in lung cancer at both RNA (Fig 2A) and protein levels (Fig 2B). In addition, high expression of RBM39 is associated with poor prognosis (Fig 2C). Intriguingly, we detected a statistically significant elevation of RBM39 expression in human lung cancer (compared with carcinoma versus para-carcinoma) using an immunohistochemistry assay (Fig 2D). To provide comprehensive data and reliable evidence for the promotion role of RBM39 in CSC-like properties and EMT, we overexpressed RBM39 in H460 and A549 cells by lentiviral infection. Consistent with the above expectations, western blotting showed increased levels of CD44, CD133, ALDH1A1, and SOX2 (Fig 2E). RBM39 overexpression also up-regulated N-cadherin and down-regulated E-cadherin compared to vector groups (Fig 2E). In the 3D spheroid cancer models, RBM39 overexpression enhanced holoclone formation (Fig 2I). Moreover, flow cytometric assessment of surface markers (CD44 and CD133) indicated that the number of CD133^+^ and CD44^+^ cells similarly increased after overexpressing RBM39 (Fig 2J). Consistent with the protein expression data, RBM39 promoted NSCLC cell proliferation (Fig 2G, 2H), migration, and invasion (Fig 2K and 2L). Conversely, RBM39 knockout reduced stemness and mesenchymal markers, increased epithelial markers (Fig 2F), and decreased CD133^+^ and CD44^+^ cells populations (Fig 2J). Furthermore, RBM39 knockout cells inhibited proliferation, spheroidic capacity, migration, and invasion (Fig 2G, 2H, 2I, 2K and 2L). Similar results were observed in the H460 cells (S3 Fig). Collectively, our findings demonstrate that RBM39 promotes CSC-like traits and EMT, thereby facilitating NSCLC progression and metastasis.

### RBM39 is methylated by PRMT6 at arginine 92

**Fig 3:**
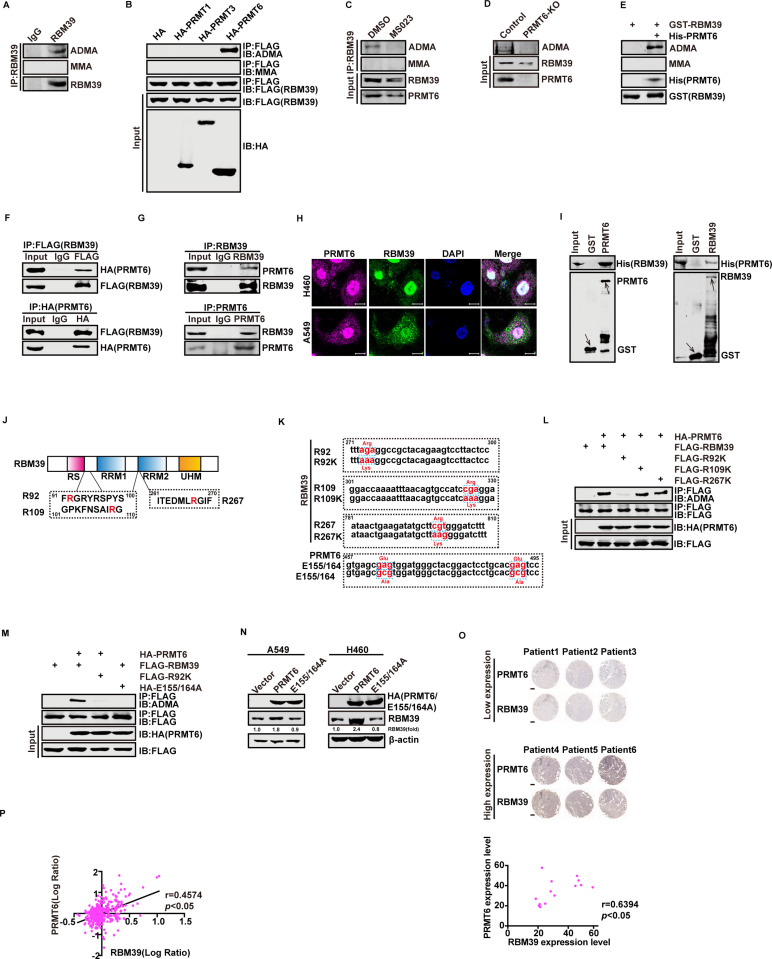
RBM39 is methylated by PRMT6 at arginine 92. **(A**) Endogenous RBM39 undergoes ADMA modification. Endogenous RBM39 immunoprecipitates from H460 cells were immunoblotted with anti-RBM39, MMA, or ADMA antibodies. (**B**) RBM39 is methylated by PRMT6 in vivo. HEK293T cells were co-transfected with FLAG-RBM39 and HA-PRMT1, PRMT3, or PRMT6. Then, total cell lysates were immunoprecipitated with anti-FLAG antibody and detected by western blotting. (**C**) MS023 decreases RBM39 methylation. Endogenous RBM39 was immunoprecipitated in H460 cells with or without MS023 treatment and then blotted with anti-ADMA and anti-MMA antibodies. (**D**) Knockout PRMT6 decreased RBM39 methylation. Endogenous RBM39 immunoprecipitated in H460 cells with PRMT6 knockout and then blotted with anti-ADMA antibody. **(E**) RBM39 is methylated by PRMT6 in vitro. Purified GST-RBM39 was incubated with or without His-PRMT6 in 60 μL of histone methyltransferase (HMT) buffer at 37 °C for 2 hours and followed by western blotting with His, GST, MMA, or ADMA antibodies. (**F** and **G**) PRMT6 interacts with RBM39 in vivo. (**F**) HEK293T cells were co-transfected with FLAG-RBM39 and HA-PRMT6. The co-IP assay was carried out using an anti-HA/FLAG antibody, followed by western blotting with an anti-FLAG/HA antibody. (**G**) Endogenous RBM39 or PRMT6 immunoprecipitated from H460 cells were immunoblotted with anti-RBM39 or anti-PRMT6 antibodies. (**H**) PRMT6 co-localizes with RBM39 in nucleus. H460 and A549 cells stained with PRMT6 antibody (purple) and RBM39 antibody (green). Nucleus, DAPI (blue). Bars = 5 μm. (**I**) PRMT6 interacts with RBM39 in vitro. GST pull-down assays were performed using His-RBM39/His-PRMT6 with GST or GST-PRMT6/GST-RBM39, followed by immunoblotting with anti-His/GST antibody. (**J-M**) PRMT6 methylates RBM39 at arginine 92. (**J**) Schematic representation of RBM39. RS, arginine-serine-rich domain; RRM1 and RRM2, RNA recognition motif domains; UHM, U2AF homology motif domain. Numbers indicate amino acid positions. (**K**) Construction of Mutation Pattern Map of RBM39 methylation site and PRMT6 enzyme activity deletion site. (**L**) HEK293T cells were co-transfected with FLAG-RBM39/R92K/R109K/R267K with HA-PRMT6 and subjected to immunoprecipitation followed by western blotting. (**M**) HEK293T cells were co-transfected with FLAG-RBM39/ FLAG-R92K with HA-PRMT6/HA-E155/164A and subjected to immunoprecipitation followed by western blotting. (**N**) A549 and H460 cells were transfected with empty vector, HA-PRMT6 or HA-E155/164A, and the protein levels of RBM39 were verified by western blotting. (**O**) IHC staining of RBM39 and PRMT6 in pathological sections of NSCLC patients (*n* = 15). A two-tailed Pearson correlation analysis method was used to calculate the correlation between the expression of RBM39 and PRMT6. Representative images were presented. Bars = 500 µm. (**P**) The protein expression levels of RBM39 and PRMT6 were positively correlated in NSCLC patients through the CPTAC database. The linear relationship was determined by a Pearson correlation analysis. The underlying data for Fig 3O and 3P can be found in S3 Data. HMT, histone methyltransferase; MMA, ω-NG-monomethyl arginine; ADMA, ω-NG, NG-asymmetric dimethylarginine; DAPI, 4′,6-diamidino-2-phenylindole; RBM39, RNA-binding protein 39; PRMT6, protein arginine methyltransferase 6.

What maintains the stable high expression of RBM39? Previously, multiple PTMs regulate protein stability, including protein arginine methylation and poly-ubiquitination [35,36]. To determine whether RBM39 is methylated at arginine residues in vivo, we performed a co-immunoprecipitation (co-IP) assay using RBM39 antibody in H460 cells, followed by detection with an ADMA antibody. As shown in Fig 3A, endogenous RBM39 underwent ADMA modification. To identify the specific methylating enzymes, we individually co-transfected HEK293T cells with FLAG-RBM39 and HA-PRMT1, 3, or 6. IP analysis indicated that RBM39 was asymmetrically di-methylated specifically by PRMT6 (Fig 3B). Treatment of H460 cells with MS023, a type I PRMT inhibitor, reduced ADMA-modified RBM39 levels, as confirmed by IP using an RBM39 antibody (Fig 3C). Similarly, PRMT6 knockout (PRMT6-KO) in H460 cells reduced asymmetric di-methylation of RBM39 (Fig 3D). To further validate the results, we performed an in vitro methylation assay. Recombinant GST-RBM39 protein was asymmetric di-methylated but not monomethylated (MMA) following the addition of PRMT6 (Fig 3E). These data confirm that PRMT6 methylates RBM39 both in vivo and in vitro. Next, we investigated whether RBM39 combines with PRMT6. We ectopically expressed HA-PRMT6 and FLAG-RBM39 in HEK293T cells and then performed co-IPs with an anti-HA or anti-FLAG antibody. The results showed that exogenous RBM39 can bind to exogenous PRMT6 in vivo (Fig 3F). This interaction was also confirmed endogenously in H460 cells (Fig 3G). Immunofluorescence staining further showed nuclear co-localization of PRMT6 and RBM39 (Fig 3H). Finally, we performed a GST pull-down assay to clarify the direct interaction between PRMT6 and RBM39, further demonstrating the PRMT6/RBM39 interaction in vitro (Fig 3I). RBM39 contains an N-terminal arginine-serine-rich (RS) domain, two RNA recognition motif domains (RRM1 and RRM2), and a C-terminal U2AF homology motif domain (UHM) (Fig 3J). Analysis of its coding sequence revealed three potential methylation sites: R92, R109, and R267. We generated site-specific mutants (R92K, R109K, R267K) and a catalytically inactive PRMT6 mutant (E155/164A) (Fig 3K) [37]. We then co-transfected HEK293T cells with FLAG-RBM39, R92K, R109K, or R267K mutants with HA-PRMT6. As shown in Fig 3L, RBM39 lost methylation in the R92K mutant group. To further confirm that PRMT6 methylated RBM39 at R92, we co-transfected HEK293T cells with FLAG-RBM39 or R92K mutant and HA-PRMT6 or HA-E155/164A. Methylation occurred only with wild-type RBM39 and PRMT6(Fig 3M), indicating that PRMT6 specifically targets R92. Furthermore, we next evaluated the effect of PRMT6 on RBM39 expression. Overexpression of wild-type PRMT6, but not E155/164A, in A549 and H460 cells increased RBM39 protein levels (Fig 3N). Immunohistochemistry of NSCLC tissue samples showed a positive correlation between PRMT6 and RBM39 expression (Fig 3O). This correlation was confirmed in the CPTAC NSCLC dataset (Fig 3P). Altogether, our results proved that RBM39 can be asymmetric di-methylated by PRMT6 at arginine 92. This methylation increases RBM39 protein levels and is associated with the progression of NSCLC.

### RBM39 methylation by PRMT6 increases RBM39 stability by inhibiting Indisulam-induced ubiquitination

**Fig 4:**
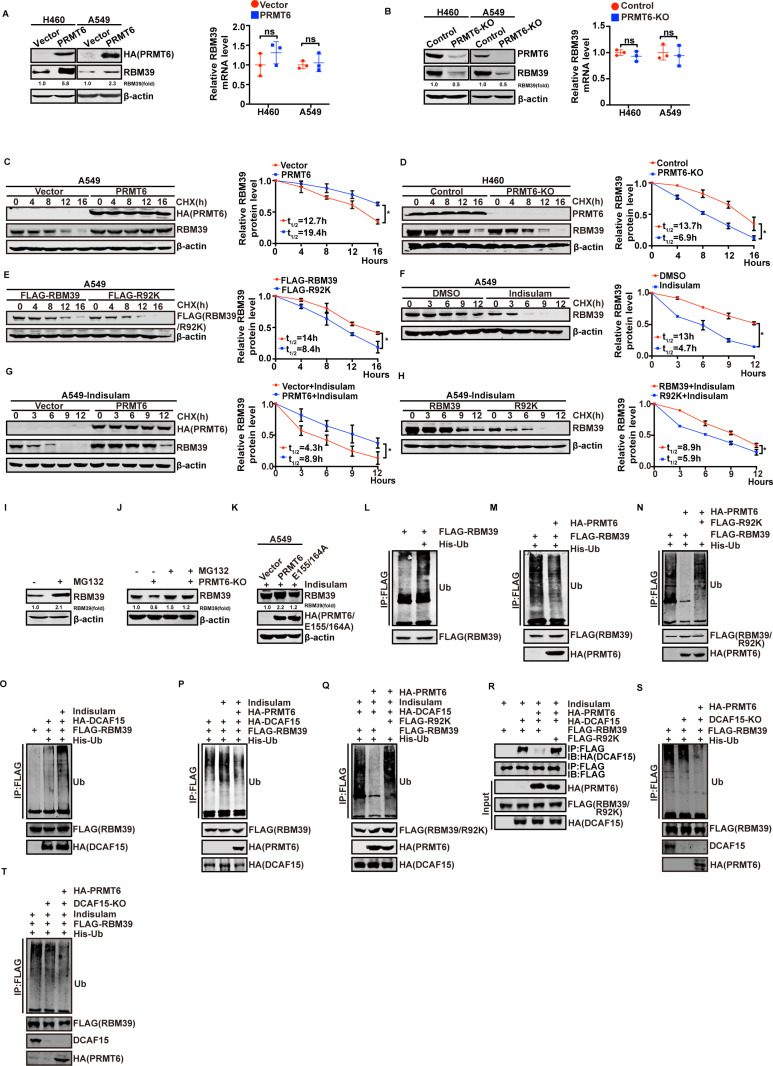
RBM39 methylation by PRMT6 increases RBM39 stability by inhibiting its ubiquitination. (**A** and **B**) PRMT6 increases RBM39 protein levels. Western blotting analysis of RBM39 protein in H460 and A549 cells transfected with empty vector or HA-PRMT6 (**A**, left)/PRMT6-KO (**B**, right). Endogenous RBM39 mRNA levels were measured by quantitative real-time PCR in H460 and A549 cells transfected with empty vector or HA-PRMT6 (**A**, left)/KO-PRMT6 (**B**, right). (*n* = 3, Student *t* test). (**C–E**) PRMT6 increases RBM39 protein stability. (**C**) A549 cells transfected with empty vector or HA-PRMT6 were treated with cycloheximide (CHX, 100 μg/mL) for 0, 4, 8, 12, or 16 hours. RBM39 protein levels were detected by western blotting (*n* = 3). (**D**) Western blotting of endogenous RBM39 expression in empty Control or PRMT6-KO transfected H460 cells when treated with CHX for the indicated times (*n* = 3). (**E**) A549 cells expressing wild-type FLAG-RBM39 or FLAG-R92K mutant were treated with CHX for the indicated times, and RBM39 protein stability was analyzed by western blotting (*n* = 3). (**F-K**) PRMT6 reverses Indisulam-induced inhibition of RBM39 protein stability. (**F**) Endogenous RBM39 expression using Indisulam in A549 cells was detected by western blotting when treated with CHX for 0, 3, 6, 9, or 12 hours (*n* = 3). (**G**) Western blotting detects RBM39 expression in empty vector- or HA-PRMT6-transfected A549 cells using Indisulam when treated with CHX for 0, 3, 6, 9, or 12 hours (*n* = 3). (**H**) The expression of RBM39 was detected by western blotting in stable expression of wild-type RBM39 and mutant R92K of A549 cells using Indisulam when treated with CHX for 0, 3, 6, 9, or 12 hours (*n* = 3). The half-life of RBM39 protein t_**1/2**_ measurement was performed as described previously. (**I**) The RBM39 protein level is elevated in the presence of MG132. H460 cells were treated with MG132 for 6 hours, and the protein level of RBM39 was measured by western blotting. (**J**) KO-PRMT6 does not decrease the RBM39 protein level in the presence of MG132. H460 cells were transfected with an empty vector or KO-PRMT6 and incubated with or without MG132 for another 6 hours. The protein level of RBM39 was detected by western blotting. (**K**) PRMT6 reverses the suppression of Indisulam on RBM39 protein. A549 cells were transfected HA-PRMT6 or HA-E155/164A with Indisulam treatment. The protein level of RBM39 was measured by western blotting. (**L–N**) PRMT6-mediated methylation of RBM39 at arginine 92 inhibits Indisulam-induced ubiquitination. (**L**) HEK293T cells were co-transfected with FLAG-RBM39 with or without His-ubiquitin and immunoprecipitated with an anti-FLAG antibody. The RBM39 ubiquitination was measured by western blotting with an anti-multiubiquitin antibody. (**M**) HEK293T cells were co-transfected with FLAG-RBM39, His-ubiquitin with or without HA-PRMT6, and immunoprecipitated with anti-FLAG antibody. The RBM39 ubiquitination was measured by western blotting with an anti-multiubiquitin antibody. (**N**) HEK293T cells were co-transfected with His-ubiquitin, FLAG-RBM39, or FLAG-R92K with or without HA-PRMT6, then immunoprecipitated with anti-FLAG antibody. The RBM39 ubiquitination was measured by western blotting with an anti-multiubiquitin antibody. (**O**) HEK293T cells were co-transfected with FLAG-RBM39 with or without His-ubiquitin and HA-DCAF15 and Indisulam, then immunoprecipitated with anti-FLAG antibody. The RBM39 ubiquitination was measured by western blotting with an anti-multiubiquitin antibody. (**P**) HEK293T cells were co-transfected with FLAG-RBM39, HA-DCAF15, His-ubiquitin with or without HA-PRMT6, and Indisulam and immunoprecipitated with anti-FLAG antibody. The RBM39 ubiquitination was measured by western blotting with an anti-multiubiquitin antibody. (**Q**) HEK293T cells were co-transfected with His-ubiquitin, HA-DCAF15, and HA-PRMT6 with FLAG-RBM39 or FLAG-R92K and immunoprecipitated with anti-FLAG antibody. The RBM39 ubiquitination was measured by western blotting. (**R**) The methylation of RBM39 by PRMT6 affects the binding of RBM39 to DCAF15. HEK293T cells using Indisulam were co-transfected with FLAG-RBM39/R92K and HA-DCAF15 with or without HA-PRMT6. The immunoprecipitation with anti-FLAG antibody, followed by western blotting. (**S**) DCAF15 was knocked out in A549 cells, which were co-transfected with His-ubiquitin and, with or without HA-PRMT6, followed by immunoprecipitation using an anti-RBM39 antibody. RBM39 ubiquitination was measured by Western blotting using an anti-multiubiquitin antibody. (**T**) DCAF15 was knocked out in A549 cells, which were then transfected with His-ubiquitin, with or without HA-PRMT6, followed by treatment with Indisulam and immunoprecipitation using an anti-RBM39 antibody. The RBM39 ubiquitination was measured by western blotting with an anti-multiubiquitin antibody. Data calculates the mean ± SD (*n* = 3). *p *< *0.05, **p *< *0.01, ***p *< *0.001. Statistical analysis was calculated using a two-tailed Student *t* test. The underlying data for Fig 4A–4H can be found in S3 Data. CHX, cycloheximide; RBM39, RNA-binding protein 39; PRMT6, protein arginine methyltransferase 6.

To clarify if PRMT6-mediated RBM39 methylation can regulate RBM39 expression, we first tested the effect of PRMT6 overexpression on RBM39 protein and mRNA levels by western blotting and quantitative real-time PCR (qRT-PCR), respectively. As shown in Fig 4A, PRMT6 overexpression increased RBM39 protein levels in both H460 and A549 cells, while mRNA levels remained unchanged. To further confirm PRMT6 function, we knocked out PRMT6 expression in H460 and A549 cells using the CRISPR/Cas9 system. PRMT6 deletion reduced RBM39 protein levels but had minimal effect on mRNA levels (Fig 4B). Thus, these results suggest that PRMT6 promotes RBM39 protein expression at the post-transcriptional level. We next examined whether PRMT6-mediated methylation stabilizes RBM39 protein. As shown in Fig 4C–4E, PRMT6 overexpression clearly increased the half-life of RBM39 from 12.7 to 19.4 hours (Fig 4C), whereas knocking out PRMT6 significantly decreased the half-life of RBM39 from 13.7 to 6.9 hours (Fig 4D). Moreover, the R92K mutant protein declined more rapidly than wild-type RBM39 protein (Fig 4E). Thus, we conclude that PRMT6-mediated methylation of RBM39 can increase RBM39 protein levels by maintaining its protein stability. To evaluate the effect of PRMT6 in the presence of Indisulam, we performed protein half-life assays. Indisulam reduced RBM39 half-life from 13 to 4.7 hours (Fig 4F). whereas PRMT6 overexpression rescued the half-life from 4.3 to 8.9 hours (Fig 4G), suggesting that PRMT6 counteracts Indisulam-induced destabilization. Finally, we compared wild-type and R92K RBM39 in Indisulam-treated A549 cells. The R92K mutant decayed more rapidly, with a half-life of 5.9 hours compared to 8.9 hours for wild-type RBM39 (Fig 4H).

To investigate how RBM39 methylation enhances its protein levels, we hypothesized that methylation stabilizes RBM39 by inhibiting its ubiquitination, which typically promotes proteasome-mediated degradation. We incubated H460 cells with MG132, a proteasomal degradation inhibitor, and assessed RBM39 protein levels by western blotting (Fig 4I). Indeed, MG132 treatment resulted in a marked increase in RBM39 levels, while PRMT6-KO did not reduce the accumulation of RBM39, indicating that PRMT6-mediated methylation increases RBM39 stability in a proteasome-dependent manner (Fig 4J). We next co-transfected PRMT6 or its inactive mutant E155/164A in the presence of Indisulam. PRMT6 restored RBM39 protein levels, whereas E155/164A did not (Fig 4K). To determine whether RBM39 methylation inhibits its ubiquitination and degradation, we used HEK293T cells expressing FLAG-RBM39 with or without His-ubiquitin, then detected RBM39 ubiquitination using an anti-multiubiquitin antibody. As shown in Fig 4L, the RBM39 protein could be ubiquitinated in vivo. In addition, RBM39 ubiquitination was decreased in HEK293T cells transfected with HA-PRMT6 (Fig 4M). Moreover, the ubiquitination level of wild-type RBM39 was markedly lower than that of R92K in HEK293T cells transfected with HA-PRMT6 (Fig 4N), suggesting that RBM39 methylation by PRMT6 at the R92 site suppresses its ubiquitination. Previous studies have reported that RBM39 is ubiquitinated by ubiquitin E3 ligase DCAF15 when using Indisulam [9], and the result has been verified (Fig 4O). We then investigated whether RBM39 methylation at R92 may inhibit ubiquitination induced by Indisulam. As shown in Fig 4P and 4Q, DCAF15 ubiquitinated RBM39 in the presence of the Indisulam, and PRMT6 attenuated wild-type RBM39 ubiquitination but had no effect on R92K. We also used other aryl sulfonamide, such as E7820, to further validate the DCAF15-dependent degradation mechanism and the inhibitory role of PRMT6 in the ubiquitination process. The results showed that PRMT6 continued to suppress the E7820-induced ubiquitination process (S4 Fig). co-IP results further confirmed that PRMT6 inhibited the binding of RBM39 to DCAF15 in the presence of Indisulam (Fig 4R). To further demonstrate that PRMT6-mediated methylation of RBM39 inhibits its ubiquitination independently of DCAF15, we knocked out DCAF15 in A549 cells and transfected with PRMT6, with or without Indisulam treatment. In all conditions, PRMT6 consistently suppressed the ubiquitination of RBM39 (Fig 4S and 4T). Our findings suggest that RBM39 methylation by PRMT6 at R92 inhibits DCAF15-mediated ubiquitination and maintains RBM39 protein stability.

### PRMT6 alleviates the suppression of metastasis and tumor cell growth caused by RBM39 knockout

**Fig 5:**
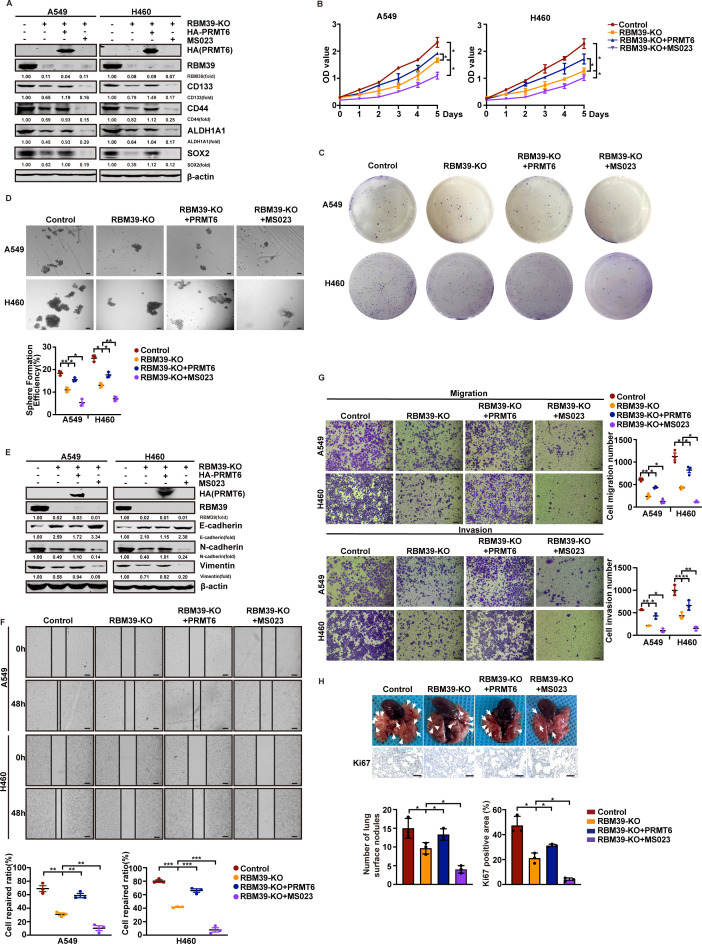
PRMT6 alleviates the suppression of metastasis and tumor cell growth caused by RBM39 knockout. **(A**) A549 and H460 cells with empty vector, RBM39-KO, RBM39-KO+PRMT6, or RBM39-KO+MS023, respectively, and the protein levels of stem-related markers were detected by western blotting. (**B** and **C**) MTT assays and colony formation assays were performed to test the cell growth ability in the cells above. The combination of RBM39 knockout and MS023 treatment in A549 and H460 cells significantly enhanced the inhibitory effects on cell proliferation (**B**) and colony formation ability (**C**). (**D**) Sphere formation assays were conducted to assess the tumor sphere-forming ability of the indicated cell lines. The combination of MS023 and RBM39 knockout significantly reduced the number of tumor spheres formed, whereas PRMT6 overexpression reduced the inhibitory effect of RBM39 knockout on tumor sphere formation. Bars = 200 μm. (**E**) Western blotting was used to assess the protein levels of EMT markers indicated cells. (**F**) The scratch wound healing assay was used to assess the motility ability of the aforementioned. Bars = 100 μm. A549 and H460 cells with MS023 treatment elevate the inhibition of NSCLC cell motility induced by RBM39 knockout, whereas PRMT6 overexpression diminished this inhibitory effect. (**G**) Transwell assays were performed to test the migratory and invasive potential of the cells above. Bars = 200 μm. The combination of RBM39 knockout and MS023 further inhibited migration and invasion compared to RBM39 knockout alone in A549 and H460 cells. In contrast, PRMT6 overexpression mitigated the inhibitory effects of RBM39 knockout on migration and invasion. (**H**) The empty control, RBM39-KO and RBM39-KO+PRMT6 overexpression stably transfected A549 cells were injected into nude mice via the tail vein. PBS or MS023 treatment after tail vein injection by A549 cells for 4 days, tumor number was measured, and all mice were sacrificed after 60 days. Nodules on the lung surface were analyzed. Visible lung tumors were quantitatively analyzed. Representative images and immunohistochemical staining of Ki67 (Bars = 200 µm) from indicated groups. The lung tumor was quantitatively analyzed by Ki67 immunohistochemical staining. Data calculate the mean ± SD (*n* = 3). *p *< *0.05, **p *< *0.01, ***p *< *0.001. Statistical analysis was calculated using a two-tailed Student *t* test. The underlying data for Fig 5B, 5D, 5F, 5G, and 5H can be found in S3 Data. NSCLC, non–small cell lung cancer; RBM39, RNA-binding protein 39; PRMT6, protein arginine methyltransferase 6; MTT, 3-(4,5-Dimethylthiazol-2-yl)-2,5-diphenyltetrazolium bromide assay.

RBM39 knockout inhibits the proliferation and metastasis of NSCLC cells, while PRMT6-mediated methylation of RBM39 promotes these processes. Considering the crucial role of PRMT6 in regulating RBM39, we next investigated the functions of PRMT6 in both in vitro and in vivo after RBM39 knockout. As shown in Fig 5A, RBM39 knockout decreased the protein levels of stemness-related markers, while PRMT6 overexpression mitigated these changes. The combination of MS023 and RBM39 knockout further enhanced the reduction in protein expression levels induced by RBM39 knockout. MTT and colony formation assay showed that PRMT6 overexpression alleviated the inhibitory effects of RBM39 knockout on tumor cell proliferation. However, MS023 further enhanced this inhibitory effect (Fig 5B and 5C). The sphere formation assay confirmed that PRMT6 overexpression reduced the inhibition of tumor sphere formation caused by RBM39 knockout, while the combination of MS023 and RBM39 knockout led to the formation of even fewer tumor spheres (Fig 5D). Next, we evaluated the role of the PRMT6-RBM39 axis in tumor metastasis. RBM39 knockout in A549 and H460 cells led to a significant decrease in N-cadherin and vimentin levels, and a notable increase in E-cadherin protein levels. Co-expression of PRMT6 relieved the RBM39-KO induced changes in these metastasis marker protein levels, while the combination of MS023 and RBM39-KO further enhanced the inhibition compared to RBM39-KO alone (Fig 5E). Next, we conducted wound healing and Transwell assays with A549 and H460 cells. The results showed that RBM39 knockout inhibited both the migration and invasion potential of these cells. However, the co-expression of PRMT6 diminished the effects of RBM39 knockout on cell migration and invasion. Additionally, the combination of RBM39 knockout and MS023 treatment further enhanced the inhibition of migration and invasion (Fig 5F and 5G). We next investigated the impact of PRMT6-RBM39 axis metastasis in vivo. A549 cells stably expressing empty vector, RBM39-KO, or RBM39-KO+PRMT6 overexpression were injected into 4-week-old Balb/c nude mice via the tail vein. Two months later, the nude mice were sacrificed, and the number of formed lung tumors were examined as a way to analyze the metastasis of the A549 cells. As shown in Fig 5H, mice injected with MS023 in combination with RBM39 knockout cells had the fewest lung tumors compared to the other three groups. PRMT6 overexpression attenuated the inhibitory effect induced by RBM39 knockout. The Ki67 results were consistent with these findings: the combination of MS023 and RBM39 knockout resulted in the lowest malignancy in mouse lung tissues, whereas PRMT6 overexpression increased the malignancy of lung tissues compared to RBM39 knockout alone. The results indicate that although PRMT6-mediated RBM39 methylation promoted the growth and metastasis of NSCLC cells, PRMT6 may also play a pro-tumor role through other target proteins when RBM39 is knocked out. Therefore, MS023 is required to inhibit PRMT6 to more effectively reduce the malignancy of NSCLC.

### RBM39 methylation reverses the inhibitory effect of Indisulam on NSCLC cells

**Fig 6:**
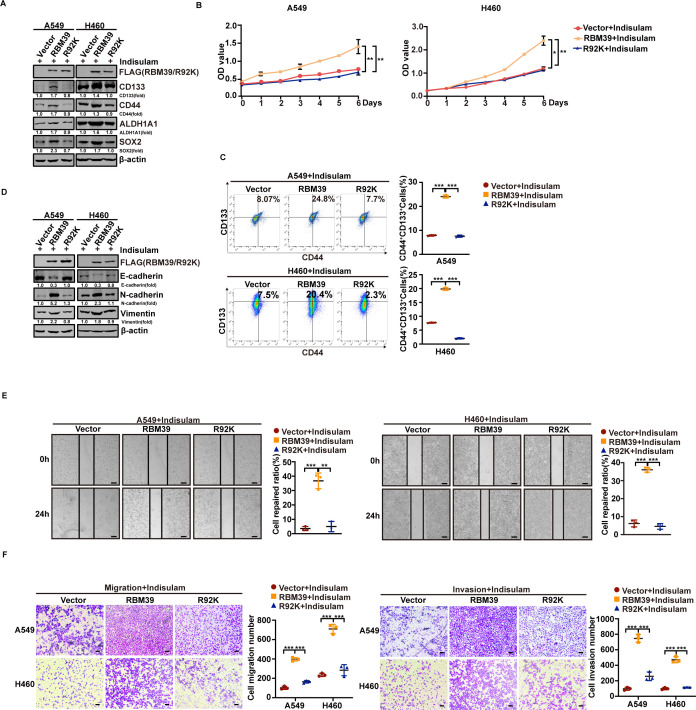
Methylation of RBM39 reverses the inhibitory of Indisulam on NSCLC. (**A**) A549 and H460 cells transfected with empty vector, RBM39, or R92K, respectively, were treated with Indisulam, and the protein levels of stem-related markers were analyzed by western blotting. (**B**) MTT assays were performed to test the cell growth ability in the cells above treated with Indisulam. (**C**) The proportion of CD44^+^ CD133^+^ cells populations in indicated groups was quantified by flow cytometry. (**D)** Western blotting was used to detect N-cadherin, vimentin, and E-cadherin levels in A549 and H460 cells transfected with the indicated constructs and treated with Indisulam. (**E**) Wound healing assays were performed to evaluate the migratory ability of transfected cells after Indisulam exposure. Bars = 100 μm. (**F**) Transwell assays assessed the migratory and invasive capacities of the indicated cells above, Bars = 200 μm. Data calculates the mean ± SD (*n* = 3). *p < 0.05, **p < 0.01, ***p < 0.001. Statistical analysis was calculated using the one-way ANOVA. The underlying data for Fig 6B, 6C, 6E, and 6F can be found in S3 Data. NSCLC, non–small cell lung cancer; RBM39, RNA-binding protein 39; MTT, 3-(4,5-Dimethylthiazol-2-yl)-2,5-diphenyltetrazolium bromide assay.

RBM39 protein is a substrate of PRMT6, which methylates it at R92 to enhance its stability. Our findings suggested that PRMT6-mediated methylation of RBM39 promotes tumorigenicity in tumor cells (S5 Fig). Therefore, we hypothesized that this methylation confers resistance to Indisulam-induced cytotoxicity. A549 and H460 cells were transfected with PRMT6, E155/164A, RBM39, and R92K and treated with Indisulam. As shown in Figs 6A and S6A, in the presence of Indisulam, PRMT6 or RBM39 raised the levels of the stem cell markers CD133, CD44, ALDH1A1, and SOX2, whereas E155/164A or R92K had no effects on these protein levels when compared with the vector control. Thus, as measured by MTT assays (Figs 6B and S6B) and flow cytometry (Figs 6C and S6C), both PRMT6 and RBM39 maintained cancer cell growth and CSC-like properties under Indisulam treatment, unlike E155/164A or R92K. These findings indicate that RBM39 methylation mitigates the suppressive effect of Indisulam on NSCLC stemness. We next assessed whether methylation of RBM39 affects Indisulam-mediated inhibition of EMT. In cells overexpressing PRMT6 or RBM39, E-cadherin levels decreased while N-cadherin and vimentin levels increased, relative to controls; no such changes were observed in R92K or E155/164A cells (Figs 6D and S6D). Overexpression of PRMT6 or RBM39 significantly promoted cell migration and invasion in the presence of Indisulam. However, R92K or E155/164A overexpression did not influence tumor cell migration and invasion rates (Figs 6E, 6F, S6E and S6F). Collectively, our data suggest PRMT6-mediated RBM39 methylation reverses Indisulam-inhibited metastasis in NSCLC cells. Together, these results suggest that PRMT6-mediated RBM39 methylation antagonizes Indisulam-induced inhibition of metastasis and supports NSCLC progression.

### RBM39 methylation reverses Indisulam-induced alterations of alternative splicing

**Fig 7:**
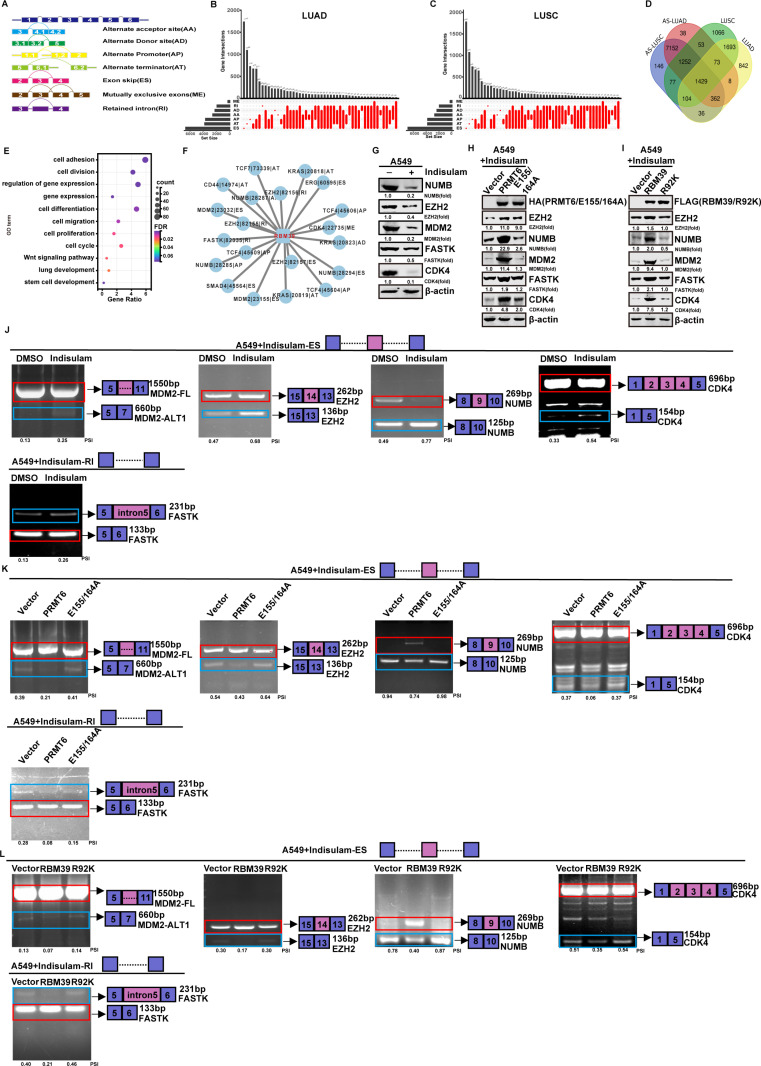
Methylation of RBM39 reverses Indisulam-induced alterations of alternative splicing. (**A**) The schema diagram illustrates several distinct types of alternative splicing (AS) events. (**B** and **C**) The alternative splicing events (SEs) take place in LUAD and LUSC. (**D**) The different genes in LUAD and LUSC are related to SEs. (**E**) The bubble plots illustrate that 1429 genes are collapsed in various Gene Ontology. (**F**) The interaction between RBM39 and SEs is illustrated in the network. (**G-I**) Western blotting assessment of expression of indicated molecules that undergo aberrant splicing. (**G**) Western blotting assessment of expression of indicated proteins that undergo aberrant splicing in A549 cells by Indisulam-induced for 48 hours. (**H**) Western blotting assessment of expression of indicated molecules that undergo aberrant splicing in A549 cells transfected with HA-PRMT6 or HA-E155/164A with Indisulam treatment. (**I**) Western blotting assessment of expression of indicated molecules that undergo aberrant splicing in A549 with stable expression of FLAG-RBM39 or FLAG-R92K with Indisulam treatment. (**J**) Reverse transcription PCR validates the mis-splicing events induced by Indisulam in A549 cells. PCR products were loaded into 1%–2% agarose gel with ethidium bromide for electrophoresis. Red color indicated the predicted molecular weight of PCR products. Blue color indicated the mis-spliced PCR products. (**K**) Reverse transcription PCR validates Indisulam-induced mis-splicing events reversed by transfection of PRMT6 in A549 cells. (**L**) Reverse transcription PCR validates Indisulam-induced mis-splicing events reversed by expression of RBM39 in A549 cells. Data calculates the mean ± SD (*n* = 3). *p *< *0.05, **p *< *0.01, ***p *< *0.001. Statistical analysis was calculated using the one-way ANOVA. The underlying data for Fig 7B and 7C can be found in GitHub (https://github.com/zhangtongjia123/my-data-project/blob/main/RBM39-README.md). The underlying data for Fig 7E and 7F can be found in S3 Data. LUAD, Lung adenocarcinoma; LUSC, Lung squamous cell carcinoma.

Aberrant alternative splicing events (SEs) are frequently observed in NSCLC, often resulting from misregulated gene splicing, alterations in splicing regulators, or disruptions in splicing control mechanisms. As a result, dysregulated alternative RNA splicing plays a fundamental role in lung cancer development [38]. A schematic diagram illustrates the main types of alternative SEs (Fig 7A) [39]. Various types of alternative SEs are observed in both LUAD and LUSC. A single alternative SE can affect multiple genes, and a single gene can exhibit different splicing patterns (Fig 7B and 7C). We used the TCGA database and applied limma analysis to identify differentially expressed genes between cancer tissues and adjacent normal tissues. We further examined whether these differentially expressed genes overlapped with genes involved in SEs. The results showed that 1,429 genes underwent alternative SEs in both types of lung cancer, exhibiting both changes in expression levels and alterations in splicing patterns (Fig 7D). These results suggest a potential link between differential gene expression and AS mechanisms in lung cancer, which may contribute to tumorigenesis and cancer progression. To gain deeper insights into the biological significance of these SEs in lung cancer, we performed Gene Ontology (GO) enrichment analysis on the 1,429 genes associated with AS events. The analysis revealed that these genes are enriched in several critical biological functions, including cell cycle regulation, cell proliferation, cell migration, and stem cell development (Fig 7E). RBM39, one of the 1429 genes, participates in a wide range of AS of pre-mRNA of many oncogenes. Therefore, we further investigated the interaction between RBM39 and SEs. Using Cytoscape software, we constructed an interaction network based on the correlation between RBM39 expression levels in lung cancer and the proportion of SEs occurring in cancer-related genes. This network revealed alternative SEs associated with RBM39, suggesting its potential regulatory role in splicing mechanisms in lung cancer (Fig 7F). Does methylation of the Indisulam target protein RBM39 influence its inhibitory effect on RBM39-mediated AS? Western blotting and RT-PCR were used to detect the protein expression and splicing patterns of some oncogenes (*NUMB, EZH2, MDM2, CDK4, FASTK*). As shown in Fig 7G and 7J, Indisulam inhibited protein levels of these oncogenes and promoted their mis-splicing. Expression of wild-type PRMT6 and RBM39 reversed the inhibitory effect of Indisulam on the protein levels of these oncogenes and reduced their mis-splicing, while the E155/164A and R92K mutants had no significant effect (Fig 7H, 7I, 7K, and 7L). We also investigated the AS patterns of downstream genes in the absence of Indisulam treatment. As shown in S7A and S7B Fig, the wild-type PRMT6 and RBM39 still increased the protein levels. However, RT-PCR analysis showed that, in the absence of Indisulam treatment, no AS isoforms were observed for the *MDM2*, *EZH2*, *CDK4*, and *FASTK* genes in the control, wild-type RBM39 and PRMT6, or mutant groups. For NUMB, the AS isoforms did not show a significant increase in either the control or mutant group, compared to the PRMT6 and RBM39 overexpression groups. (S7C and S7D Fig). The results indicate that RBM39 methylation reverses Indisulam-induced alterations of AS, thereby promoting protein expression of oncogenes.

### Indisulam combined with MS023 can more strongly inhibit malignant proliferation and metastasis of NSCLC

**Fig 8:**
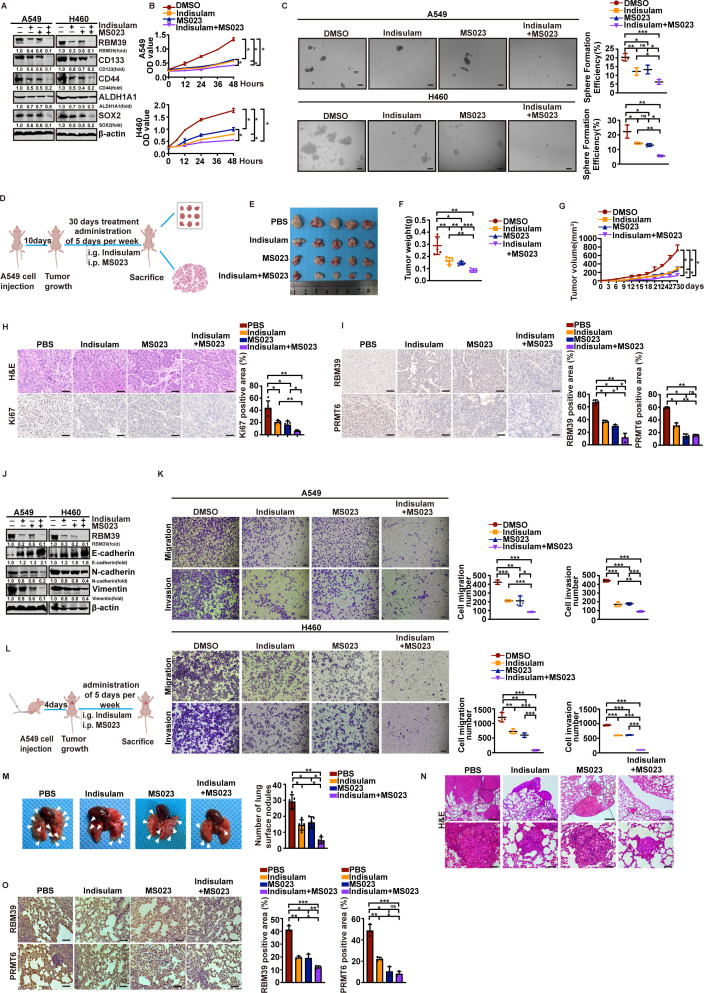
Indisulam combined with MS023 more strongly inhibits malignant proliferation and metastasis of NSCLC. (**A–L**) Indisulam combined with MS023 more strongly inhibits the stemness and proliferation of NSCLC cells. (**A**) The protein levels of stemness markers were measured by western blotting in A549 and H460 cells treated with DMSO, Indisulam, MS023, or their combination. (**B**) Cell proliferation of A549 and H460 cells under indicated treatment was evaluated by MTT assays. (**C**) Sphere formation assays evaluated self-renewal capacity in the indicated group. Bars = 200 μm. (**D**) The experimental protocol of Indisulam was combined with MS023 to treat malignant proliferation of mouse NSCLC. A549 xenografts developed tumors in BALB/c nude mice. The mice were randomly assigned to treatment with PBS, Indisulam, MS023, or combination (5 mice per group). Indisulam was administered by oral gavage daily at 25 mg/kg, and MS023 was intraperitoneally injected at 50 mg/kg daily. Error bars indicate SEM. After 30 days, tumor size (**E**), tumor weight (**F**), and tumor volume (**G**) were measured and analyzed. (**H**) Representative images of H&E staining (top panels, Bars = 200 µm) and immunohistochemical staining of Ki67 (bottom panels, Bars = 200 µm) from indicated groups. The A549 xenograft tumor was quantitatively analyzed by Ki67 immunohistochemical staining after treatment with PBS, Indisulam, MS023, or Indisulam+MS023. (**I**) Representative IHC images of RBM39 and PRMT6 expression in the indicated groups of tumor tissues. (**J–O**) Indisulam plus MS023 reduces NSCLC metastasis in vitro and in vivo. (**J**) EMT-related proteins were analyzed by western blotting in A549 and H460 cells treated as above. (**K**) Transwell assays were used to examine the invasion and migration of the cells above. Bars = 200 μm. (**L**) The experimental protocol of Indisulam was combined with MS023 in the treatment of metastasis in mouse NSCLC. PBS, Indisulam, MS023, or Indisulam+MS023 treatment after tail vein injection by A549 cells for 4 days, tumor number was measured, and all mice were sacrificed after 60 days. Nodules on the lung surface were analyzed. (**M–O**) The lung lesions and representative lung tissue sections are shown from the nude mice treated with PBS, Indisulam, MS023, or Indisulam+MS023. (**M**) Arrows indicate metastatic focus of lung cancer cells on the surface of the lung. (**N**) Representative images of H&E staining show the metastatic nodules on the lung surface. Bars = 200 µm. (**O**) The expression of RBM39 and PRMT6 positive cells ratio of lung tissue sections in indicated mice using IHC assay. Bars = 200 µm. Data calculates the mean ± SD (*n* = 3). *p *< *0.05, **p *< *0.01, ***p *< *0.001. Statistical analysis was calculated using the one-way ANOVA. The underlying data for Fig 8B, 8C, 8E–8I, 8K and 8M–8O can be found in S3 Data. IHC, immunohistochemistry; NSCLC, non–small cell lung cancer.

Since methylation of RBM39 can reverse the effects of Indisulam on NSCLC, is inhibiting RBM39 methylation also a potential strategy for NSCLC treatment? MS023, a known PRMT6 inhibitor, was used to test whether it could enhance Indisulam’s therapeutic effect by suppressing PRMT6-mediated RBM39 methylation. As expected, the combination of Indisulam and MS023 significantly decreased RBM39 protein levels and the expression of stem cell markers CD133, CD44, ALDH1A1, and SOX2 (Fig 8A). Similarly, co-treatment more effectively suppressed NSCLC cell proliferation than either agent alone (Figs 8B and S8A). Sphere formation assays and flow cytometry showed that this combination also diminished cancer stem-like characteristics (Figs 8C and S8B). Additionally, we next evaluated the in vivo efficacy of this combination using a nude mouse NSCLC model treated with PBS, Indisulam, MS023, or both drugs (Fig 8D). Tumor growth was slower in the combination group compared to single-agent treatments (Fig 8E—8G). Histological (H&E) and immunohistochemical staining (Fig 8H) showed a lower percentage of Ki67-positive cells and reduced malignant features in tumors from the combination group.

To further evaluate RBM39 and PRMT6 expression patterns, immunohistochemistry of tumor sections indicated significant down-regulation of both proteins in the combination group relative to the DMSO, Indisulam, or MS023 groups (Fig 8I). These results indicate that co-treatment effectively inhibits malignant proliferation of NSCLC in vitro and in vivo. We then tested whether MS023 enhances Indisulam’s anti-metastatic activity. As shown in Fig 8J, the combination more significantly decreased the expression of mesenchymal markers (N-cadherin, vimentin) and increased epithelial marker E-cadherin. Cell invasion and migration were more significantly inhibited than with Indisulam alone (Figs 8K and S8D). These results indicate that Indisulam combined with MS023 treatment can significantly inhibit metastasis of NSCLC cells. Given the apparent sensitizing effect of MS023 on Indisulam in NSCLC cells, we then used a lung metastasis model (Fig 8L), we found that the combination group had fewer metastatic lesions than the PBS or single-treatment groups (Fig 8M). H&E staining confirmed fewer tumor foci in the co-treated group (Fig 8N). Furthermore, Immunohistochemistry further showed lower expression of PRMT6 and RBM39 compared to single treatments (Fig 8O). As shown in Fig S8C and S8E, no significant differences in ALT, AST, CREA, and UREA levels were detected among groups, indicating no apparent liver or kidney toxicity. In conclusion, Indisulam combined with MS023 effectively inhibits NSCLC tumor growth and metastasis in vitro and in vivo, providing a new therapeutic option for treating NSCLC.

## Discussion

NSCLC is a common malignancy associated with high incidence and mortality. Surgical resection remains the only curative option for patients with early-stage disease. However, most cases are diagnosed at an advanced stage [40–42]. First-line pembrolizumab plus chemotherapy for NSCLC has revolutionary improvements, nearly 40% of patients exhibit primary resistance and poor prognosis [43–45]. These findings highlight the need for effective second-line treatment options.

**Fig 9:**
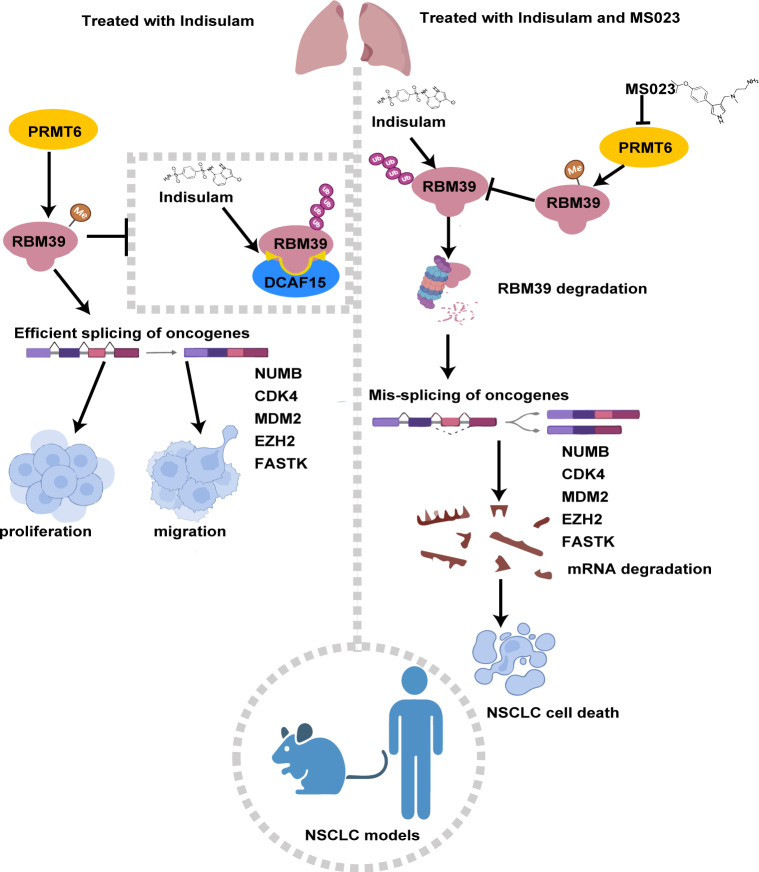
Model of methylation RBM39 by PRMT6 enhances Indisulam resistance in NSCLC. PRMT6 methylates RBM39 at R92 in NSCLC cells. Methylation of RBM39 improves the stability of RBM39 protein via decelerating Indisulam-induced ubiquitination and proteasomal degradation. The methylated RBM39 reverses the mis-splicing of oncogenes caused by Indisulam and increases the expression level of oncogenes, thus promoting the malignant proliferation and metastasis of cancer cells and enhancing the resistance of Indisulam. The combination of Indisulam with MS023, a PRMT6 inhibitor, suppresses RBM39 methylation, reduces its protein stability, and enhances Indisulam sensitivity in NSCLC cells. Therefore, our study provides a new drug target and treatment option for the NSCLC.

Indisulam has been proposed as a potential second-line therapy for NSCLC [7]. Indisulam-induced cytotoxicity and mis-splicing events have been attributed to RBM39 depletion[46]. Moreover, Indisulam resistance limits its clinical efficacy in advanced NSCLC [7]. Our data suggest that aberrant RBM39 methylation may contribute to Indisulam resistance and represent a therapeutic target. In this study, PRMT6 was found to methylate RBM39 at R92, which interferes with DCAF15 binding and prevents Indisulam-induced RBM39 degradation, thereby stabilizing the protein. RBM39 methylation reverses Indisulam-induced mis-splicing, up-regulates oncogene expression, and contributes to tumor progression and drug resistance in NSCLC cells. These findings provide a mechanistic basis for rational drug combinations in NSCLC. In combination with the PRMT6 inhibitor MS023 enhances Indisulam activity by blocking RBM39 methylation and promoting oncogene mis-splicing. A summary model is shown in Fig 9.

PRMT6 is a type I PRMT involved in diverse biological and pathological processes. Its tumor-promoting function is mediated by methylation of histone and non-histone substrates, influencing AS, gene expression, cell proliferation, and drug resistance. Therefore, PRMT6 has emerged as a therapeutic target in several cancer types[47–52]. PRMT6 interacts with p16 and methylates it at R22, R131, and R138, which disrupts its interaction with CDK4 and promotes cell cycle progression. [53,54]. It also methylates p21 at R156; facilitating its phosphorylation and resulting in cytoplasmic accumulation of the protein. Cytoplasmic p21 contributes to increased resistance to cytotoxic agents in cancer cells [55]. PRMT6 also contributes to sorafenib in hepatocellular carcinoma (HCC). It methylates CRAF at R100, altering ERK-mediated nuclear translocation of PKM2 and regulating PKM2-dependent glycolysis. Thus, the PRMT6-ERK-PKM2 regulatory axis is implicated in tumorigenesis and sorafenib resistance in HCC [56]. Collectively, PRMT6 plays a crucial role in mediating drug resistance. Additionally, PRMT6 regulates AS of pre-mRNA. PRMT6-mediated methylation of PTEN at R159 modulates the PI3K-AKT pathway and global alternative pre-mRNA splicing, affecting multiple physiological processes [37]. However, the mechanism by which PRMT6 regulates AS remains unclear. Our study identified RBM39 as a novel substrate of PRMT6. PRMT6-mediated methylation of RBM39 sustains oncogene AS, thereby up-regulating oncogene expression and promoting proliferation, metastasis, and drug resistance in NSCLC cells.

PRMT6-mediated methylation stabilizes RBM39 and counteracts the effects of Indisulam on its ubiquitination. RBM39 methylation can reverse the inhibition of NSCLC cell proliferation, migration, and invasion induced by Indisulam. Protein arginine methylation is implicated in several cancers, suggesting its potential as a therapeutic target [57]. identifying a demethylase could slow cancer progression, metastasis, and therapy resistance. Jumonji domain-containing 6 (JMJD6) is the first and currently the only known arginine demethylase [58]. JMJD6 demethylates some PRMT1 targets [59,60]. However, in contrast, our study shows that JMJD6 promotes PRMT6-mediated methylation of RBM39, leading to increased RBM39 expression (S9A and S9B Fig). These results illustrate that the targets of PRMTs and JMJD6 do not precisely overlap. JMJD6 can’t demethylate the PRMT6 target protein RBM39. The RBM39-JMJD6 interaction is independent of enzymatic activity in clear cell renal cell carcinoma [61]. Based on these findings, we propose that JMJD6 increases RBM39 protein levels independently of its enzymatic activity, thereby reducing the sensitivity of NSCLC cells to Indisulam. The precise mechanism by which JMJD6 regulates RBM39 and its role in Indisulam resistance remains to be elucidated. Our study highlights the role of RBM39 arginine methylation in Indisulam resistance and suggests that RBM39 demethylation could represent a promising therapeutic strategy. Accordingly, the PRMT6 inhibitor MS023 blocks RBM39 methylation, promotes Indisulam-induced ubiquitination, accelerates RBM39 degradation, and thereby reduces Indisulam resistance. While the combination of MS023 and Indisulam demonstrates significant anti-tumor effects, we acknowledge the potential limitations of using this broad-spectrum inhibitor. MS023 may also inhibit other Type I PRMTs, including PRMT1 and PRMT4, which play roles in RNA splicing, transcriptional regulation, and cancer progression [62–64]. Consequently, the observed phenotypic changes may not be entirely attributable to the inhibition of PRMT6. While our experimental conditions suggest that PRMT6 inhibition is the dominant effect, MS023, which exhibits higher inhibitory activity toward PRMT6 and reduces RBM39 methylation levels, may also affect other PRMTs. Future studies should consider using more selective PRMT6 inhibitors or CRISPR/Cas9-based gene knockout techniques to eliminate potential interference from other PRMTs [65]. In summary, we propose a new therapeutic option for Indisulam combined with MS023 in treating NSCLC.

RBM39 is a critical RBP involved in transcriptional co-regulation and RNA splicing, and plays an essential role in tumor-associated mRNA and protein expression [28]. RBM39 is up-regulated as a splicing factor in a variety of cancers, including NSCLC, liver cancer, and gastric cancer, and its loss is lethal to cancer cells. Preclinical studies suggest that aryl sulfonamide-induced RBM39 degradation is a promising cancer therapy, causing widespread splicing errors and strongly inhibiting cancer cell proliferation in vitro and in vivo [19,20,26]. Our results show that Indisulam induces aberrant splicing of pro-oncogenic genes in NSCLC. Fig 7G–7L illustrates that overexpression of RBM39 and PRMT6 can reverse the aberrant splicing induced by Indisulam. However, western blot results showed that overexpression of PRMT6 and RBM39 significantly increased the expression of *EZH2*, *NUMB*, and *MDM2* genes, while PCR results revealed there were no significant differences between the main bands, and the differences observed only existed in the aberrant splice variants. We believe that the inconsistency between protein differences and splicing differences can be attributed to two factors: 1) Primer Design for Specific Splicing Events: During primer design, we focused on detecting specific exon or intron changes, which could result in the amplification of multiple splice isoforms within the main band. This may lead to a lack of clear trends in the main band, as it could represent a mixture of various splicing variants rather than a single isoform. 2) Antibody Specificity Issues: The antibodies used in western blot may not differentiate between different splice isoforms or post-translationally modified protein forms. In future studies, we aim to address these issues by sequencing DNA gels and developing isoform-specific antibodies to enhance our results. These strategies will improve our ability to align mRNA SEs with protein-level changes, thereby providing a more accurate understanding of the underlying mechanisms. Additionally, we examined the splicing patterns upon overexpressing RBM39 and PRMT6 without Indisulam treatment. The results revealed that, compared to the control group, overexpression of both wild-type and mutant forms did not induce changes in splicing patterns or generate splice variants. This suggests that aberrant splicing occurs only when RBM39 levels are reduced or knocked out, impairing splice site recognition and leading to mis-splicing. On the other hand, overexpression of RBM39 and PRMT6 increases the protein level and stability of RBM39 but does not induce AS. Finally, we observed that wild-type RBM39 and PRMT6 can reverse the aberrant splicing induced by Indisulam, whereas the methylation-deficient mutant (R92K) and the enzymatically inactive mutant (E155/164A), although expressed at similar levels to the wild-type proteins through FLAG/HA tag antibody in cells, fail to reverse the mis-splicing caused by Indisulam. This further confirms that RBM39 methylation is the key factor in reversing Indisulam-induced aberrant splicing. These findings underscore the critical role of RBM39 methylation in regulating splicing fidelity and its potential implications for therapeutic resistance.

Since RBM39 is a target of Indisulam, which induces its ubiquitination and subsequent degradation [19], it holds significant potential for clinical applications and drug development. However, as a monotherapy, Indisulam shows limited efficacy in NSCLC patients, with most experiencing only short-term benefits [7]. Clinical studies have shown only modest anti-tumor effects, possibly due to the persistently high expression of RBM39 in NSCLC. Our study found that methylation of RBM39 blocks Indisulam-driven ubiquitination, stabilizes RBM39 protein, and counteracts the drug’s anti-tumor effects. We propose that RBM39 methylation is a key driver of Indisulam resistance in NSCLC cells, which may explain its limited clinical efficacy. However, Indisulam also arrests the cell cycle at the G1 phase and strongly inhibits proliferation[66], which further suppresses stemness, migration, and invasion. This complicates determining whether the suppression of tumor cell migration, invasion, and stemness is solely due to RBM39 inhibition or is also influenced by the reduced proliferative capacity caused by Indisulam. Future studies should explore new experimental methods to distinguish the effects of proliferation from those on migration, invasion, and stemness, to clarify the specific mechanisms involved. Consequently, we used MS023 to inhibit PRMT6-mediated methylation of RBM39 and promote Indisulam-induced ubiquitination. These findings suggest a new combinational treatment strategy for NSCLC.

In conclusion, PRMT6-mediated RBM39 methylation reverses Indisulam-induced oncogene mis-splicing and diminishes its anti-tumor activity. Inhibiting RBM39 methylation increases NSCLC sensitivity to Indisulam. Therefore, combining MS023 with Indisulam may produce a stronger anti-tumor effect and represent a promising strategy for treating advanced NSCLC.

## Materials and methods

### Ethics statement

The study for mice is compliant with all relevant ethical regulations for animal experiments. All experiments and facilities were approved by the Committee for Ethics of Animal Experiments and were conducted in conformity to the Guidelines for Animal Experiments, Peking University Health Science Center (LA2020230).

### Cell culture, transfection, drug treatment, and lentivirus infection

HEK293T, A549, H460, and PC9 were obtained from ATCC and cultured in Dulbecco’s modified Eagle medium supplemented with 10% fetal bovine serum (FBS) at 37 °C with 5% CO_2_. Cells were transfected using TurboFect transfection reagent (Thermo Scientific, R0533) according to the manufacturer’s protocol and harvested 48 hours after transfection to detect transfection efficiency. pLVX-IRES-Puro-RBM39, pLVX-IRES-Puro-R92K and pLVX-IRES-Puro-PRMT6 were transfected in A549 and H460 cells to acquire the stable expression. For lentiviral transduction, the lentivirus plasmid pLL3.7-shRBM39 was transfected into H460 cells. The lentivirus plasmid pLL3.7-shRBM39 expresses shRNA targets RBM39 mRNA (RBM39 sh-1: 5′-GCTTCGAGTGCTAGTTCATTT-3′ and RBM39 sh-2: 5′-AAACATGTTAGAGAGTTGG-3′), All cells were selected with puromycin for 1 week and then analyzed for overexpression and knockdown efficiency. For the design of sgRNA in ZhangFeng library, sgRNA targets RBM39 5′-GCTTGAGGCTCCTTACAAGA-3′ and sgRNA targets PRMT6 5′-GCGAGTGCTACTCGGACGTTT-3′ which was inserted in CRISPR vector pSpCas9(BB)−2A-puro. Cells were cultured for one month, and the knockout efficiency was detected. Cell lines were detected every three months. All cells used for experiments were maintained for less than 20 passages. Cells were treated with drug (vehicle control 0.1% DMSO, A549 2μM Indisulam, H460 2μM Indisulam, and HEK293T 2μM Indisulam).

### Western blotting and antibodies

Cells were lysed in RIPA lysis buffer (Thermo Scientific) containing protease inhibitor cocktail (Sigma), and the BCA protein assay kit (Pierce) was applied to measure the protein concentration. Protein lysates were separated by SDS-PAGE and were transferred to nitrocellulose membranes. The primary antibodies used were as follows: anti-RBM39 (Proteintech, 21339-1-AP), anti-PRMT6 (Proteintech, 15395-1-AP), anti-CD44 (BioLegend, 397502), CD133 (BioLegend, 397902), anti-ALDH1A1 (BioLegend, 861901), anti-SOX2 (BioLegend, 656102), anti-Vimentin (Santa Cruz, sc-373717), anti-E-cadherin (Santa Cruz, sc-8426), anti-N-cadherin (Santa Cruz, sc-8424), anti-Flag (Sigma-Aldrich, F1804), anti-HA (BioLegend,614852), anti-Mono-Methyl Arginine (Cell Signaling Technology, 8015), anti-Asymmetric Di-Methyl Arginine (Cell Signaling Technology, 13522), and anti-β-actin (ABclonal, AC026). Secondary antibodies included anti-mouse IgG antibody DyLight 800 conjugated (EarthOx, E032810) and anti-rabbit IgG antibody DyLight 800 conjugated (EarthOx, E032820). The Odyssey infrared imaging system (LI-COR Bioscience, Lincoln, NE) was performed to acquire the infrared fluorescence image.

### Quantitative real-time PCR (qRT-PCR)

Total cell RNA was extracted using the RNA simple Total RNA Kit, and then reverse transcription was performed utilizing the Revertaid First Strand cDNA synthesis kit. Then, quantitative PCR was performed using Maxima SYBR Green qPCR Master Mix according to the manufacturer’s instructions. The primers’ sequences used were listed in S1 Table. β-actin was applied as an internal control to normalize mRNA expression. Data were analyzed using the ΔΔCt.

### Immunoprecipitation

Cells were harvested and washed with ice-cold PBS. Then, cells were lysed in IP lysis buffer (25 mM Tris-HCl (pH 7.4), 150 mM NaCl, 1% Nonidet P-40, 1 mM EDTA, 5% glycerol, and 1% protease inhibitor cocktail) at 4 °C for 1 hour. and proteins were incubated with antibodies and protein A-Sepharose (GE Healthcare) at 4 °C overnight. The samples were washed with IP lysis buffer three times. The samples were eluted with 2 × SDS loading buffer and boiled for 10 min at 95 °C. Western blotting was performed to detect precipitated proteins.

### GST pull-down

Control GST and GST-tagged proteins were purified from Escherichia coli strain BL21 (DE3). The collected proteins were incubated with glutathione-sepharose beads (GE Healthcare) on a rocking platform overnight at 4 °C. The following day, the samples were incubated with His-tagged proteins for 4 hours at 4 °C. After incubation, beads were washed three times with ice-cold elution buffer, boiled together with SDS-PAGE loading buffer, and detected by western blotting with the indicated antibodies.

### In vitro methylation

The purified GST-RBM39 and His-PRMT6 were incubated with HMT buffer (25 mM Tris-HCl pH 8.8, 25 mM NaCl, 2 μM SAM). The reactions were incubated at 37 °C for 2 hours.

### In vivo ubiquitination

Cells were transfected with plasmids for 36 hours and treated with 10 μM MG132 for 6 hours before harvesting. The cells were lysed with immunoprecipitation (IP) lysis buffer. Then, samples were immunoprecipitated and incubated with indicated antibody and protein A-sepharose (GE Healthcare) on a rocking platform overnight at 4 °C. The next day, the samples were washed three times and subjected to western blotting with the indicated antibodies.

### Protein half-life assay

Each dish was added with the cycloheximide (CHX) for 0, 3, 6, 9, and 12 hours separately at a final concentration of 100 μg/mL. Then, we collected and lysed cells. The endogenous RBM39 levels were detected by western blotting and normalized to β-actin using Image J software. The half-life of RBM39 protein t_1/2_ measurement was performed as described previously [67].

### MTT

Approximately 2,000 cells were seeded in 96-well plates. After incubation at a different time, 15 μL MTT solution (5 mg/mL) was added to each well and incubated for approximately 4 hours at 37 °C. The medium was removed, and 200 μL DMSO was added to each well to dissolve the formazan crystals. The absorbance at 490 nm was read using the microplate reader. The MTT assay was performed on three biological replicates, and each replicate was measured at least three times.

### Cell migration and invasion

5 × 10^4^ cells were seeded in each chamber cultured with serum-free Dulbecco’s Modified Eagle Medium (DMEM) medium in the Transwell inserts (Corning) containing 8 μm permeable pores and allowed to migrate into lower chambers filled with DMEM supplemented with 10% FBS. For the invasion assay, each upper chamber was precoated with Matrigel. The cells were then added to chambers. Twenty-four hours later, the metastatic cells were fixed with 4% paraformaldehyde and stained with 0.1% crystal violet. Then, the cells were photographed by microscopy (Leica) and counted using Image J software.

### Colony formation

About 3 × 10^3^ were seeded in 6-cm plates and cultured with 3 mL of DMEM medium, including 10% FBS. The culture medium was refreshed every 3 days. After incubation for 2 weeks, the colonies were washed with PBS, fixed with 4% paraformaldehyde, and stained with 0.1% crystal violet. Cell samples were then photographed, and stained colonies were counted.

### Immunohistochemistry analysis

Lung cancer samples from patients were purchased from Outdo Biotech. IHC of RBM39 and PRMT6 expression were performed using RBM39 and PRMT6 antibodies (1:500).

### Immunofluorescence staining

Transfected cells were cultured in 3.5 cm confocal dishes and washed with PBS at room temperature three times. Then, cells were fixed with 4% paraformaldehyde for 15 min and permeabilized (using 0.2% Triton X-100 in PBS) for 15 min at 37 °C. After washing with PBS three times, cells were blocked for 1 hour with 1% bovine serum albumin in PBS at room temperature and incubated with primary antibodies overnight at 4 °C. The following day, cells were incubated with the secondary antibodies conjugated with AlexaFlour488 (anti-rabbit IgG) and AlexaFlour549 (anti-mouse IgG). Images were photographed using a ZEISS fluorescence microscope.

### Flow cytometry

A549 and H460 cells were grown to 80% confluence and were treated with Indisulam overnight. In our study, Cell surface CD44 and CD133 were labeled using an anti-CD44-APC antibody and an anti-CD133-PE antibody. Isotype controls were used as negative controls. Data were analyzed using FlowJo V.10.0.

### Sphere formation assay

Cells (5,000 cells/mL) were cultured in ultralow adhesion plates in DMEM/F12 supplemented with 20 ng/mL EGF, 10 ng/mL bFGF, B27 (1:50), and 10 ng/mL LIF. After the cells were cultured for two weeks, tumor spheres with a diameter of >75 μm were counted.

### Validation of alternative splicing events by reverse transcription PCR

RNA extracted from wild-type and treated cells was subjected to reverse transcription, and splicing patterns were assayed by reverse transcription PCR (RT-PCR). PCR primers were designed to amplify two or more splicing isoforms of different sizes. The primer pairs are listed in S1 Table. Then, the amplified products were analyzed on a 1% agarose gel.

### Database and computational data analysis

RNA level and protein level of RBM39 analysis through Gene Expression Profiling Interactive Analysis (GEPIA, gepia.cancer-pku.cn) and the University of Alabama at Birmingham cancer data analysis Portal (UALCAN, https://ualcan.path.uab.edu/). The AUC values of cell lines were downloaded from the Cancer Therapeutics Response Portal (CTRP, https://portals.broadinstitute.org). The cell lines’ gene expression was downloaded from the Cancer Cell Line Encyclopedia (CCLE, https://portals.broadinstitute.org/ccle/data) data portal. The protein expression levels of RBM39 and PRMT6 in lung cancer patients were analyzed through the Clinical Proteomic Tumor Analysis Consortium (CPTAC, https://cptac-data-portal.georgetown.edu) database. The splicing events were downloaded from the TCGA Spliceseq (https://bioinformatics.mdanderson.org/). The RNA-Seq data for LUSC, LUAD, and normal lung tissue were downloaded from the TCGA database via the Genomic Data Commons Data Portal (https://portal.gdc.cancer.gov). Database for Annotation, Visualization, and Integrated Discovery (DAVID, http://david.abcc.ncifcrf.gov/) was used to explore the KEGG pathway enrichment analysis. Cytoscape visualized the regulatory network. Then, we analyzed the differentially expressed genes (DEGs) and alternative splicing events by R.

### Animals

All experiments followed the guidelines of the Care and Use of Laboratory Animals of the Health Science Center of Peking University. The mice were housed and maintained under 12 hours dark/light cycles, and free access to food and water was provided. Six- to eight-week-old virgin BALB/c nude mice were purchased from the Peking University Health Science Animal Center. To establish a subcutaneous tumorigenesis model, 5 × 10^6^ A549 cells were injected into the flanks of 6-week-old BALB/c nude mice (five mice per experimental group). The mice were randomly assigned to treatment with vehicle, Indisulam, or combination. Indisulam was administered by oral gavage at 25 mg/kg and MS023 by intraperitoneal injection at 50 mg/kg. Tumor size was measured, and all mice were sacrificed after four weeks. For the metastasis analysis, 3 × 10^6^ cells were injected into the tail vein of 6-week-old BALB/c nude mice (five mice per group). Then, four days after injection, mice from each group were randomly divided into control (DMSO), Indisulam, and Indisulam+MS023 groups (five mice per group). The drugs were administered as above. Mice were euthanized at week 8, and lung tissue samples were harvested for future analysis.

### Statistical analysis

Statistical analysis was performed with GraphPad Prism 9 and SPSS 24.0. All independent experiments were repeated at least three times. All statistical results are represented as mean ± SD (standard deviation). Statistical analysis was calculated using the Student *t* test. * *p* < 0.05, ** *p* < 0.01, *** *p* < 0.001. P values of less than 0.05 were considered statistically significant.

## Supporting information

S1 Fig RBM39 promotes Indisulam resistance in NSCLC cells. **A** and **B** The MTT (*n* = 3) assay was conducted to confirm that RBM39 promotes cell proliferation. (**A**) MTT assay showed that A549 cells with RBM39 overexpression enhanced the proliferation of A549 cells. (**B**) MTT assay showed that H460 cells with RBM39 knockdown inhibited cell proliferation. (**C** and **D**) Colony formation assay was utilized to test the cell colony formation ability. (**C**) A549 cells with RBM39 overexpression treated with Indisulam promoted colony formation ability. (**D**) H460 cells with RBM39 knockdown treated with Indisulam inhibited colony formation ability. (**E** and **F**) The proportion of CD44^+^CD133^+^ cells in indicated groups was analyzed using flow cytometry. A549 cells with RBM39 overexpression treated with Indisulam elevated the amounts of CD44^+^CD133^+^ cells (**E**). H460 cells with RBM39 knockdown treated with Indisulam diminished the numbers of CD44^+^CD133^+^ cells (**F**). (**G** and **H**) The scratch wound healing assay was used to determine the motility ability in the cell mentioned above. Bars = 100 μm. A549 cells with RBM39 overexpression reversed Indisulam-induced suppression of NSCLC cell motility (**G**). H460 cells with RBM39 knockdown enhanced the inhibition of NSCLC cell motility induced by Indisulam (**H**). Data calculates the mean ± SD (*n* = 3). *p *< *0.05, **p *< *0.01, ***p *< *0.001. Statistical analysis was calculated using a two-tailed Student t test. The underlying data for S1A-B, S1E-F, and S1G–S1H Fig can be found in S3 Data.
(TIF)

S2 Fig RBM39 knockout does not enhance the inhibitory effects of Indisulam. (**A–D**) RBM39 knockout does not enhance inhibitory effects of Indisulam on stemness and proliferation. (**A**) The combination of RBM39 knockout and Indisulam did not enhance the suppression of CSC marker protein expression compared to Indisulam treatment alone. (**B** and **C**) MTT and colony formation assays were performed to assess cell proliferation. The combination of RBM39 knockout and Indisulam treatment in A549 and H460 cells did not enhance the inhibitory effects on cell proliferation (**B**) and colony formation ability (**C**). (**D**) Sphere formation assays were conducted to assess the tumor sphere-forming ability of the indicated cell lines. The combination of RBM39 knockout and Indisulam treatment did not enhance the inhibition of sphere formation. Bars = 200 μm. (**E–G**) RBM39 knockout does not improve inhibitory effects of Indisulam on migration and invasion. (**E**) RBM39 knockout does not increase the inhibition of EMT marker protein levels by Indisulam. Western blotting was used to assess the protein levels of EMT markers indicated cells. (**F**) The scratch wound healing assay was used to assess the motility ability of the aforementioned. Bars = 100 μm. A549 and H460 cells with RBM39 knockout did not elevate the inhibition of NSCLC cell motility induced by Indisulam. **G** Transwell assays were performed to test the migratory and invasive potential of the cells above. Bars = 200 μm. RBM39 knockout combined with Indisulam did not further inhibit migration and invasion compared to Indisulam alone in A549 and H460 cells. Data calculates the mean ± SD (*n* = 3). *p *< *0.05, **p *< *0.01, ***p *< *0.001. Statistical analysis was calculated using the one-way ANOVA. The underlying data for S2B, S2D, S2F, and S2G Fig can be found in S3 Data.
(TIF)

S3 Fig RBM39 promotes NSCLC cell stemness and EMT. (**A**) Western blotting showed the protein levels of CSC surface markers and EMT-related markers in H460 cells transfected with pLVX-IRES-Puro-RBM39 or pLVX-IRES-Puro-Vector (as a control). β-actin was used as a control, respectively. (**B**) RBM39 knockout affects the expression of EMT-related markers and CSC surface markers in H460 cells. Western blotting was performed on H460 cells with or without RBM39 knockout to detect the protein levels. (**C**) MTT (*n* = 3) was used to test the cell proliferation viability on H460 with RBM39 overexpression or knockout. (**D**) colony formation assay was used to test colony-forming abilities on H460 with RBM39 overexpression or knockout. (**E**) Sphere formation assays (*n* = 3) were performed in indicated cell lines to detect the sphere-forming ability. RBM39 overexpression improves sphere-forming ability, and RBM39 knockout reduces sphere-forming ability. Representative images (**E**, left) and sphere number analysis (**E,** right) were shown. Bars = 200 μm. (**F**) The proportion of CD44^+^ CD133^+^ cells in each group was analyzed using flow cytometry. RBM39 overexpression enhances the number of CD133 and CD44 positive cells. RBM39 knockout reduces the number of CD133 and CD44 positive cells. Representative dot plots (**F**, left) and percentage of CD44^+^ CD133^+^ cells (**F,** right) were shown. (**G**) The effect of RBM39 on H460 motile ability was measured by scratch wound healing assay. RBM39 overexpression promotes H460 cells motility. RBM39 knockout inhibits H460 cells motility. Bars = 100 μm. (**H**) Transwell assays were performed to test the migratory and invasive potential of the H460. RBM39 overexpression promotes H460 cells migratory and invasive. RBM39 knockout blocks H460 cells migratory and invasive. Bars = 200 μm. Data calculates the mean ± SD (*n* = 3). *p *< *0.05, **p *< *0.01, ***p *< *0.001. Statistical analysis was calculated using a two-tailed Student t test. The underlying data for S3C and S3E–S3H Fig can be found in S3 Data.
(TIF)

S4 Fig PRMT6 suppresses the E7820-induced RBM39 ubiquitination process. (**A**) HEK293T cells were co-transfected with FLAG-RBM39 with or without His-ubiquitin and HA-DCAF15 and E7820, then immunoprecipitated with anti-FLAG antibody. The RBM39 ubiquitination was measured by western blotting with an anti-multiubiquitin antibody. (**B**) HEK293T cells were co-transfected with FLAG-RBM39, HA-DCAF15, His-ubiquitin with or without HA-PRMT6, and E7820 and immunoprecipitated with anti-FLAG antibody. The RBM39 ubiquitination was measured by western blotting with an anti-multiubiquitin antibody.
(TIF)

S5 Fig RBM39 methylation promotes metastasis and growth of NSCLC cells. (**A** and **B**) A549 and H460 cells transfected with empty vector, PRMT6, or E155/164A, or empty vector, RBM39, or R92K, respectively, and the protein levels of stem-related markers were detected by western blotting. (**C** and **D**) MTT assays were performed to test the cell growth ability in the cells above. (**E** and **F**) Sphere formation assays were performed in indicated cell lines. Bars = 200 μm. Cells with RBM39 and PRMT6 overexpression increased the numbers of sphere-forming. (**G** and **H**) A549 and H460 cells were transfected with empty vector, PRMT6 or E155/164A, RBM39, or R92K. The protein levels of N-cadherin, vimentin, and E-cadherin in the indicated cells above were detected by western blotting. (**I** and **J**) The scratch wound healing assay measured the motile ability of the indicated cells above. Bars = 100 μm. (**K** and **L**) Transwell assays were performed to test the migratory and invasive potential of the indicated cells above, Bars = 200 μm. Data calculate the mean ± SD (*n* = 3). *p < 0.05, **p < 0.01, ***p < 0.001. Statistical analysis was calculated using the one-way ANOVA. The underlying data for S5C–S5F and S5I–S5L Fig can be found in S3 Data.
(TIF)

S6 Fig Methylation of RBM39 reverses the inhibition of Indisulam on NSCLC. (**A**) A549 and H460 cells transfected with empty vector, PRMT6, or E155/164A, respectively, were treated with Indisulam, and the protein levels of stem-related markers were detected by western blotting. (**B**) MTT assays were performed to test the cell growth ability in the cells above treated with Indisulam. (**C**) The proportion of CD44^+^ CD133^+^ cells in indicated groups was analyzed using flow cytometry. (**D**) A549 and H460 cells were transfected with empty vector, PRMT6 or E155/164A with Indisulam treatment. The protein levels of N-cadherin, vimentin, and E-cadherin in the indicated cells above were detected by western blotting. (**E**) The scratch wound healing assay measured the motile ability of the indicated cells above. Bars = 100 μm. (**F**) Transwell assays were performed to test the migratory and invasive potential of the indicated cells above, Bars = 200 μm. Data calculates the mean ± SD (*n* = 3). *p < 0.05, **p < 0.01, ***p < 0.001. Statistical analysis was calculated using the one-way ANOVA. The underlying data for S6B–S6C and S6E–S6F Fig can be found in S3 Data.
(TIF)

S7 Fig RBM39 methylation does not reverse alternative splicing patterns without Indisulam. (**A** and **B**) Western blotting assessment of the expression of indicated molecules that undergo aberrant splicing. (**A**) Western blotting assessment of expression of indicated molecules that undergo aberrant splicing in A549 cells transfected with HA-PRMT6 or HA-E155/164A. (**B**) Western blotting assessment of the expression of indicated molecules that undergo aberrant splicing in A549 with stable expression of FLAG-RBM39 or FLAG-R92K. (**C**) Reverse transcription PCR validates mis-splicing events induced by transfection of PRMT6 or E155/E164A in A549 cells. PCR products were loaded into 1%–2% agarose gel with ethidium bromide for electrophoresis. Red color indicated the predicted molecular weight of PCR products. Blue color indicated the mis-spliced PCR products. (**D**) Reverse transcription PCR validates mis-splicing events induced by expression of RBM39 or R92K in A549 cells.
(TIF)

S8 Fig Indisulam combined with MS023 more strongly inhibits the malignant proliferation and metastasis of NSCLC. (**A**) Colony formation assay was performed to assess the clonogenic ability of A549 and H460 cells treated with DMSO, Indisulam, MS023, or Indisulam plus MS023. (**B**) Flow cytometry was used to quantify the CD44^+^ CD133^+^ cancer stem-like cell populations in the indicated treatment group. Bars = 200 μm. (**C**) Serum levels of ALT, AST, UREA, and CREA were measured in nude mice bearing subcutaneous xenografts under different treatment conditions. (**D**) Scratch wound healing assay was used to evaluate the migratory ability of A549 and H460 cells after treatment with DMSO, Indisulam, MS023, or their combination. Bars = 100 μm. (**E**) Serum levels of ALT, AST, UREA, and CREA were also assessed in a metastatic mouse model following the same treatments. *p < 0.05, **p *< *0.01, ***p *< *0.001. Statistical analysis was calculated using the one-way ANOVA. The underlying data for S8B–S8E Fig can be found in S3 Data.
(TIF)

S9 Fig JMJD6 enhances the methylation of RBM39 in NSCLC. (**A**) JMJD6 overexpression increases the protein level of RBM39. (**B**) JMJD6 overexpression increases ADMA levels in vivo. FLAG-RBM39 and HA-PRMT6/JMJD6 were individually co-transfected into HEK293T cells. Total cell lysates were then immunoprecipitated using anti-FLAG antibodies and analyzed by western blotting.
(TIF)

S1 Table Key Resource Table. (DOCX)

S1 Data PCR quantification data for Fig 7J–7L. (XLSX)

S2 Data Western blot quantification data for Figs 1C–1F, 2E, 2F, 3N, 4A, 4B, 4I–4K, 5A, 5E, 6A, 6D, 7G–7I, 8A, 8J, S2A, S3A, S3B, S5A, S5B, S5G, S5H, S6A, S6D, S7A, S7B, and S9A. (XLSX)

S3 Data Underlying data for Figs 1–8 and S1–S8. (XLSX)

S1 Raw Images Original scanned images for Figs. (PDF)

## Abbreviations

ADMA: ω-NG, NG-asymmetric dimethylarginine
co-IP: co-immunoprecipitation
CSC: cancer stem cell
CTRP: Cancer Therapeutics Response Portal
DEGs,: differentially expressed genes;
GST: glutathione S-transferase
HCC,: hepatocellular carcinoma
IP: immunoprecipitation
LCLC: large cell lung carcinoma
LUAD: lung adenocarcinoma
LUSC: lung squamous cell carcinoma
MMA: ω-NG-monomethyl arginine
MTT: 3-(4,5-dimethylthiazol-2-yl)-2,5-diphenyltetrazolium bromide assay
NSCLC: non-small cell lung cancer
PRMT6: protein arginine methyltransferase 6
qRT-PCR: quantitative real-time PCR
RBM39: RNA-binding protein 39
RIPA: radioimmunoprecipitation assay
SDMA: ω-NG, NG-symmetric dimethylarginine
SDS: sodium dodecyl sulfate
SDS-PAGE: sodium dodecyl sulfate-polyacrylamide gel electrophoreses
shRNA: small hairpin RNA
TCGA: The Cancer Genome Atlas
