# Supplementary figures and images for: Methylation of RBM39 by PRMT6 enhances resistance to Indisulam in non-small cell lung cancer by promoting alternative splicing of proto-oncogenes
Source: PLoS Biol. 2025 Jun 4;23(6):e3002846. doi: 10.1371/journal.pbio.3002846 (PMC12142651; doi:10.1371/journal.pbio.3002846)

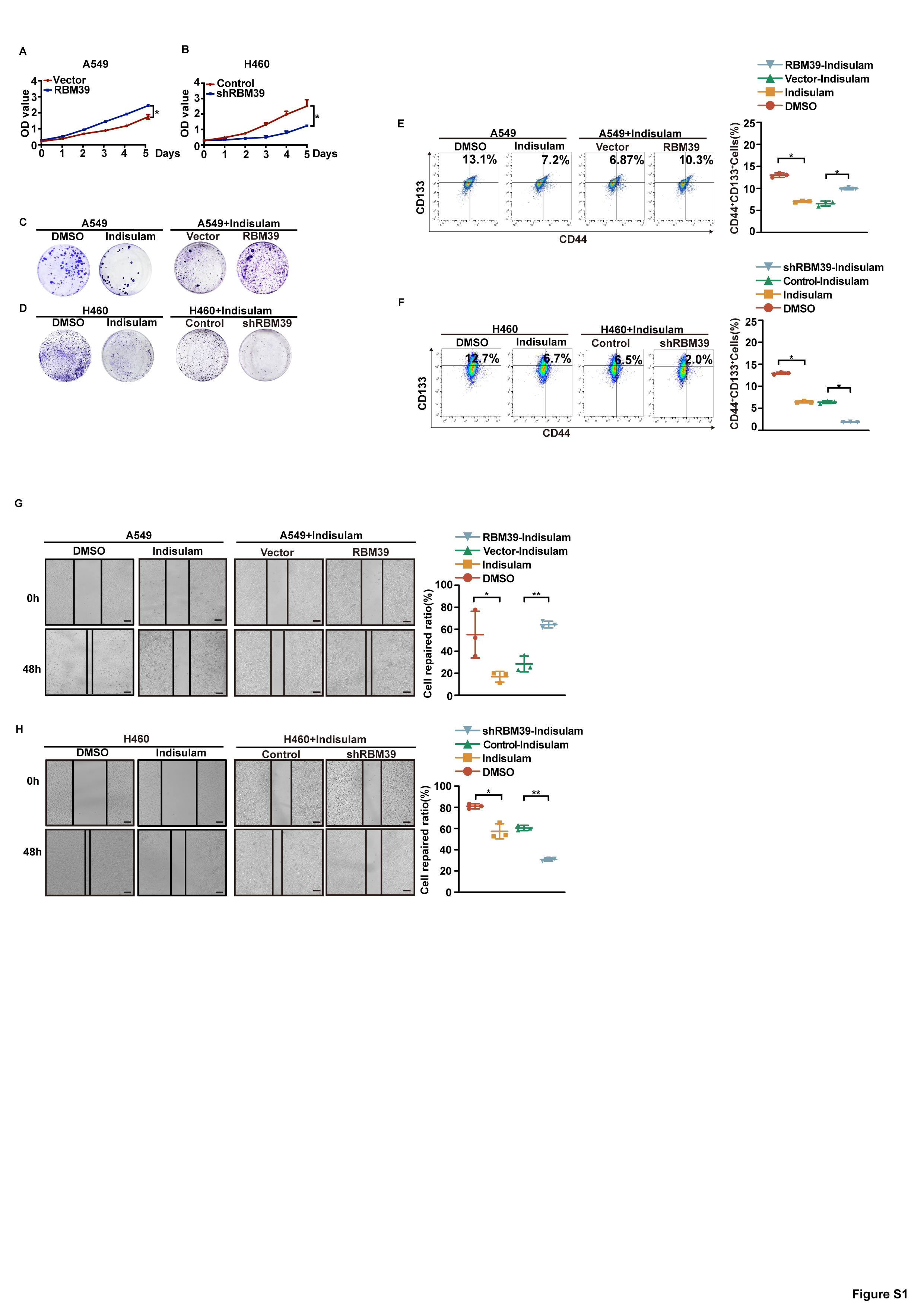

Supplement: S1 Fig — A and B The MTT (n = 3) assay was conducted to confirm that RBM39 promotes cell proliferation. (A) MTT assay showed that A549 cells with RBM39 overexpression enhanced the proliferation of A549 cells. (B) MTT assay showed that H460 cells with RBM39 knockdown inhibited cell proliferation. (C and D) Colony formation assay was utilized to test the cell colony formation ability. (C) A549 cells with RBM39 overexpression treated with Indisulam promoted colony formation ability. (D) H460 cells with RBM39 knockdown treated with Indisulam inhibited colony formation ability. (E and F) The proportion of CD44+CD133+ cells in indicated groups was analyzed using flow cytometry. A549 cells with RBM39 overexpression treated with Indisulam elevated the amounts of CD44+CD133+ cells (E). H460 cells with RBM39 knockdown treated with Indisulam diminished the numbers of CD44+CD133+ cells (F). (G and H) The scratch wound healing assay was used to determine the motility ability in the cell mentioned above. Bars = 100 μm. A549 cells with RBM39 overexpression reversed Indisulam-induced suppression of NSCLC cell motility (G). H460 cells with RBM39 knockdown enhanced the inhibition of NSCLC cell motility induced by Indisulam (H). Data calculates the mean ± SD (n = 3). *p < 0.05, **p < 0.01, ***p < 0.001. Statistical analysis was calculated using a two-tailed Student t test. The underlying data for S1A-B, S1E-F, and S1G–S1H Fig can be found in S3 Data. (TIF) [file pbio.3002846.s001.tif]

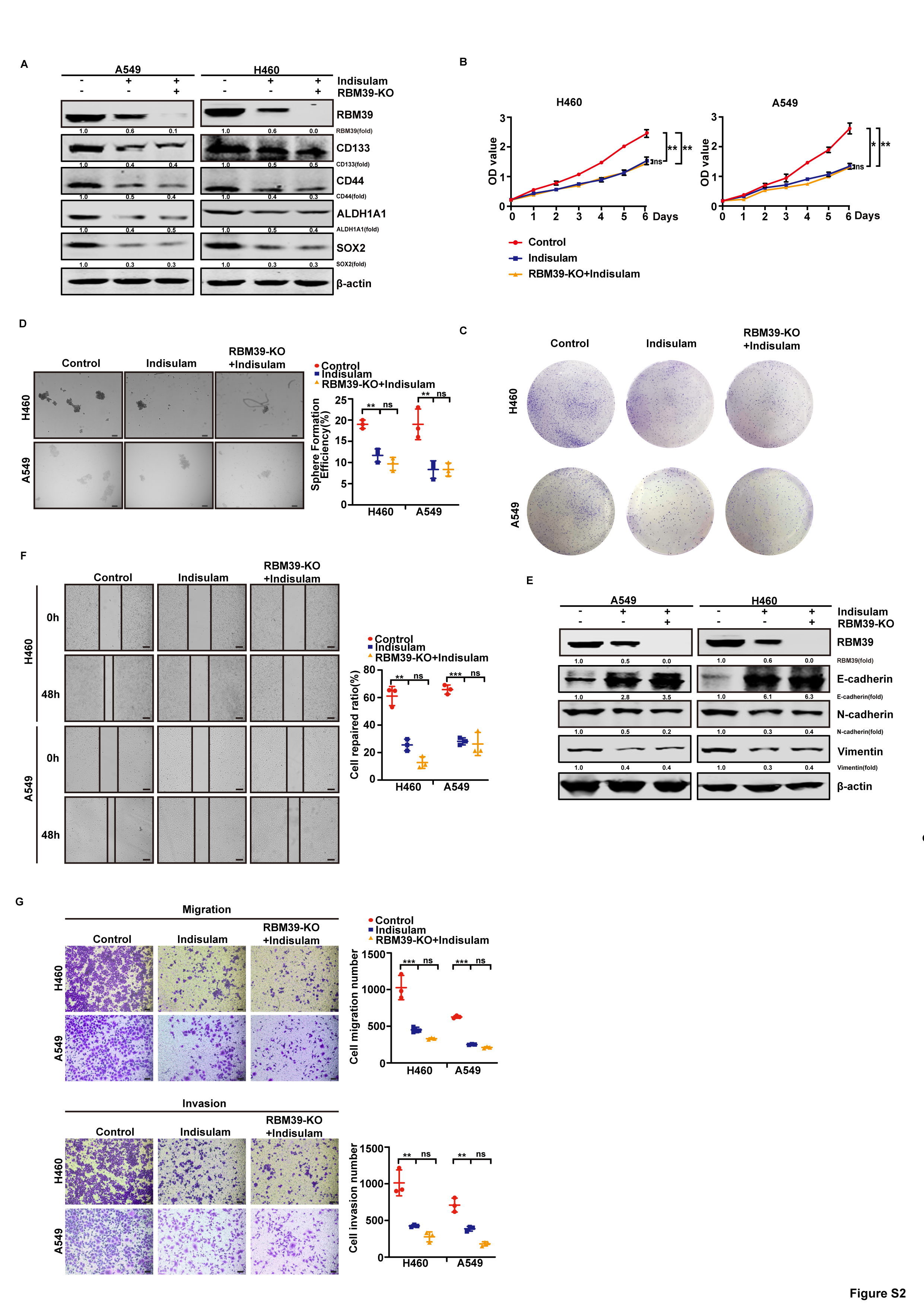

Supplement: S2 Fig — (A–D) RBM39 knockout does not enhance inhibitory effects of Indisulam on stemness and proliferation. (A) The combination of RBM39 knockout and Indisulam did not enhance the suppression of CSC marker protein expression compared to Indisulam treatment alone. (B and C) MTT and colony formation assays were performed to assess cell proliferation. The combination of RBM39 knockout and Indisulam treatment in A549 and H460 cells did not enhance the inhibitory effects on cell proliferation (B) and colony formation ability (C). (D) Sphere formation assays were conducted to assess the tumor sphere-forming ability of the indicated cell lines. The combination of RBM39 knockout and Indisulam treatment did not enhance the inhibition of sphere formation. Bars = 200 μm. (E–G) RBM39 knockout does not improve inhibitory effects of Indisulam on migration and invasion. (E) RBM39 knockout does not increase the inhibition of EMT marker protein levels by Indisulam. Western blotting was used to assess the protein levels of EMT markers indicated cells. (F) The scratch wound healing assay was used to assess the motility ability of the aforementioned. Bars = 100 μm. A549 and H460 cells with RBM39 knockout did not elevate the inhibition of NSCLC cell motility induced by Indisulam. G Transwell assays were performed to test the migratory and invasive potential of the cells above. Bars = 200 μm. RBM39 knockout combined with Indisulam did not further inhibit migration and invasion compared to Indisulam alone in A549 and H460 cells. Data calculates the mean ± SD (n = 3). *p < 0.05, **p < 0.01, ***p < 0.001. Statistical analysis was calculated using the one-way ANOVA. The underlying data for S2B, S2D, S2F, and S2G Fig can be found in S3 Data. (TIF) [file pbio.3002846.s002.tif]

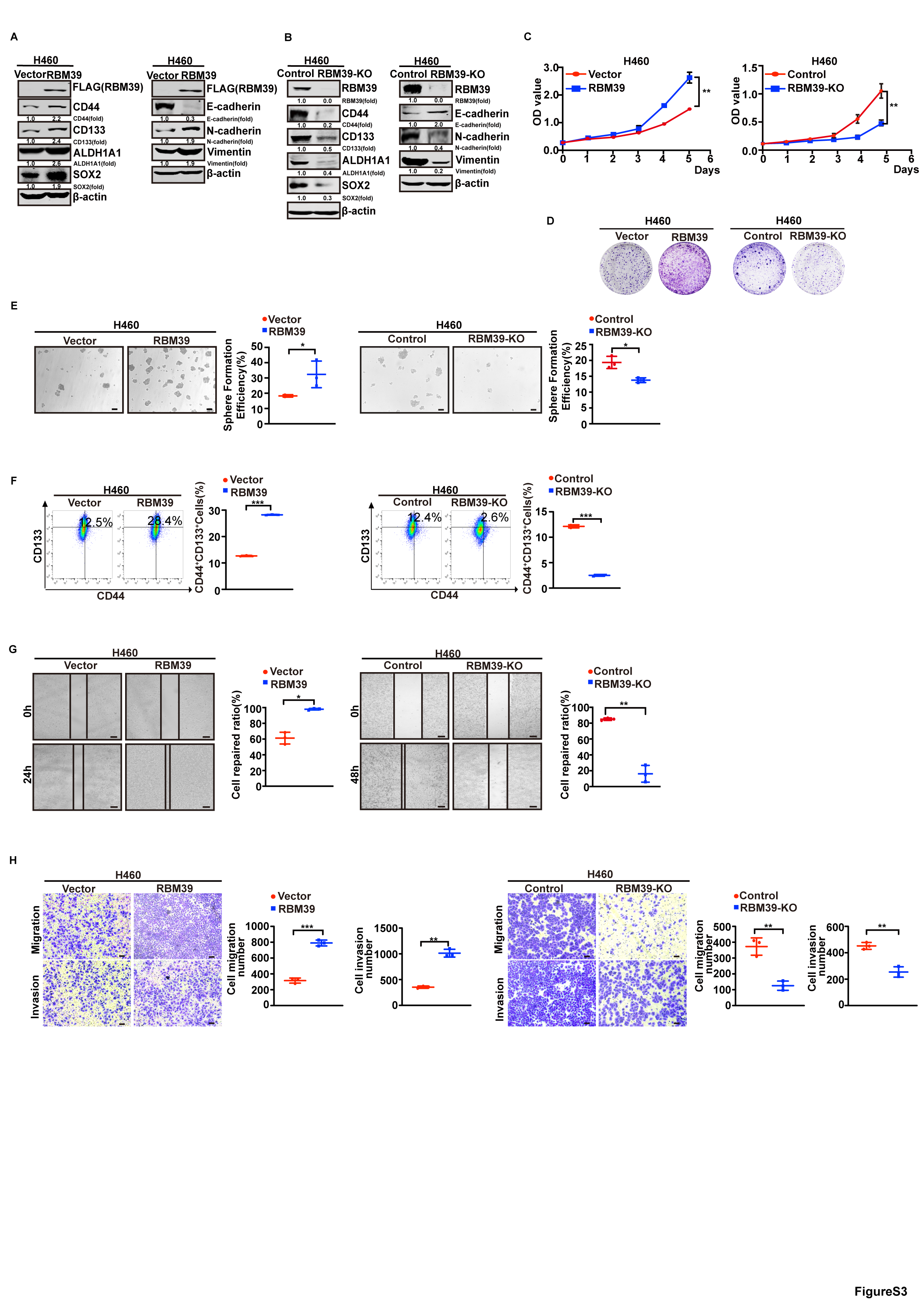

Supplement: S3 Fig — (A) Western blotting showed the protein levels of CSC surface markers and EMT-related markers in H460 cells transfected with pLVX-IRES-Puro-RBM39 or pLVX-IRES-Puro-Vector (as a control). β-actin was used as a control, respectively. (B) RBM39 knockout affects the expression of EMT-related markers and CSC surface markers in H460 cells. Western blotting was performed on H460 cells with or without RBM39 knockout to detect the protein levels. (C) MTT (n = 3) was used to test the cell proliferation viability on H460 with RBM39 overexpression or knockout. (D) colony formation assay was used to test colony-forming abilities on H460 with RBM39 overexpression or knockout. (E) Sphere formation assays (n = 3) were performed in indicated cell lines to detect the sphere-forming ability. RBM39 overexpression improves sphere-forming ability, and RBM39 knockout reduces sphere-forming ability. Representative images (E, left) and sphere number analysis (E, right) were shown. Bars = 200 μm. (F) The proportion of CD44+ CD133+ cells in each group was analyzed using flow cytometry. RBM39 overexpression enhances the number of CD133 and CD44 positive cells. RBM39 knockout reduces the number of CD133 and CD44 positive cells. Representative dot plots (F, left) and percentage of CD44+ CD133+ cells (F, right) were shown. (G) The effect of RBM39 on H460 motile ability was measured by scratch wound healing assay. RBM39 overexpression promotes H460 cells motility. RBM39 knockout inhibits H460 cells motility. Bars = 100 μm. (H) Transwell assays were performed to test the migratory and invasive potential of the H460. RBM39 overexpression promotes H460 cells migratory and invasive. RBM39 knockout blocks H460 cells migratory and invasive. Bars = 200 μm. Data calculates the mean ± SD (n = 3). *p < 0.05, **p < 0.01, ***p < 0.001. Statistical analysis was calculated using a two-tailed Student t test. The underlying data for S3C and S3E–S3H Fig can be found in S3 Data. (TIF) [file pbio.3002846.s003.tif]

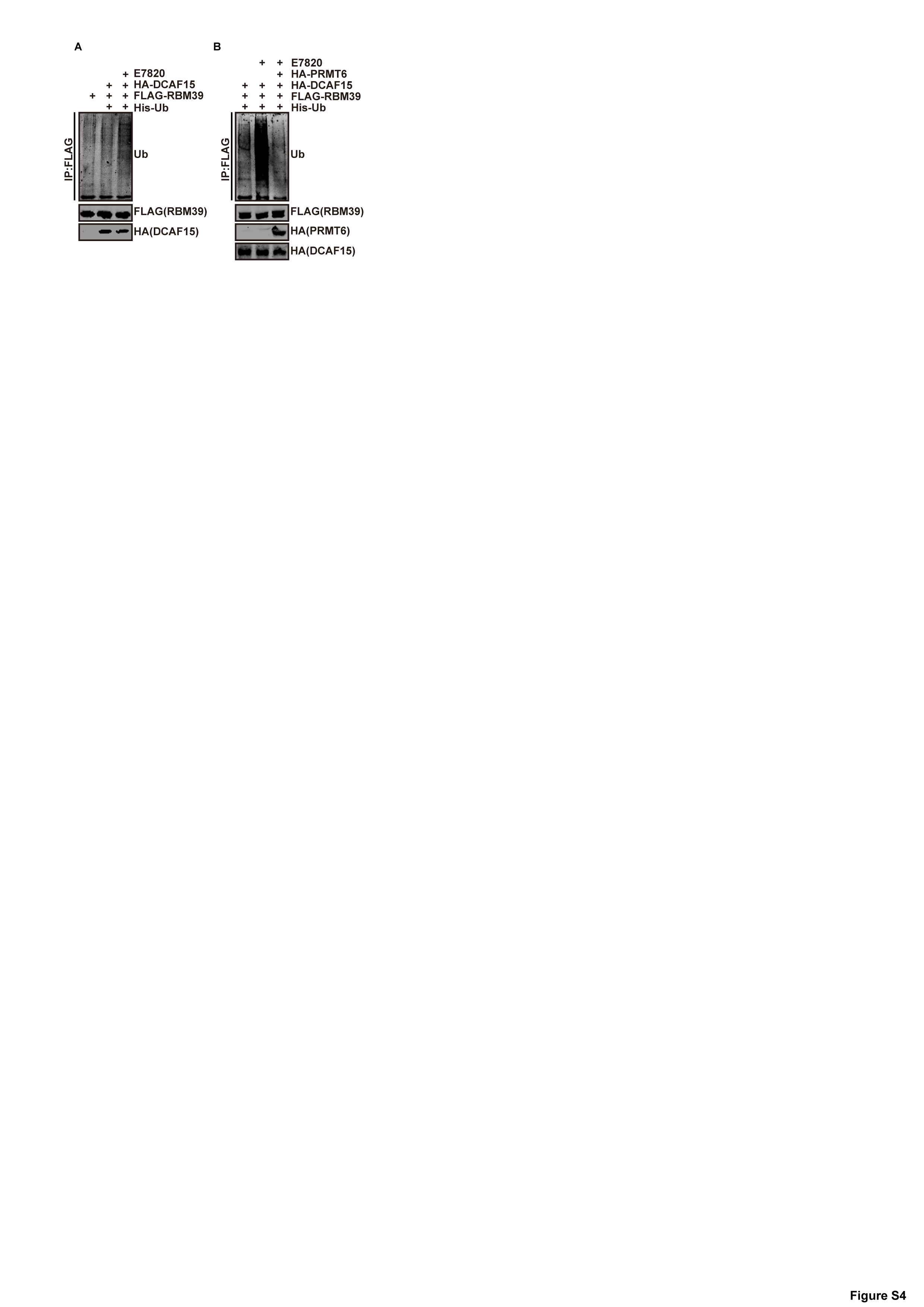

Supplement: S4 Fig — (A) HEK293T cells were co-transfected with FLAG-RBM39 with or without His-ubiquitin and HA-DCAF15 and E7820, then immunoprecipitated with anti-FLAG antibody. The RBM39 ubiquitination was measured by western blotting with an anti-multiubiquitin antibody. (B) HEK293T cells were co-transfected with FLAG-RBM39, HA-DCAF15, His-ubiquitin with or without HA-PRMT6, and E7820 and immunoprecipitated with anti-FLAG antibody. The RBM39 ubiquitination was measured by western blotting with an anti-multiubiquitin antibody. (TIF) [file pbio.3002846.s004.tif]

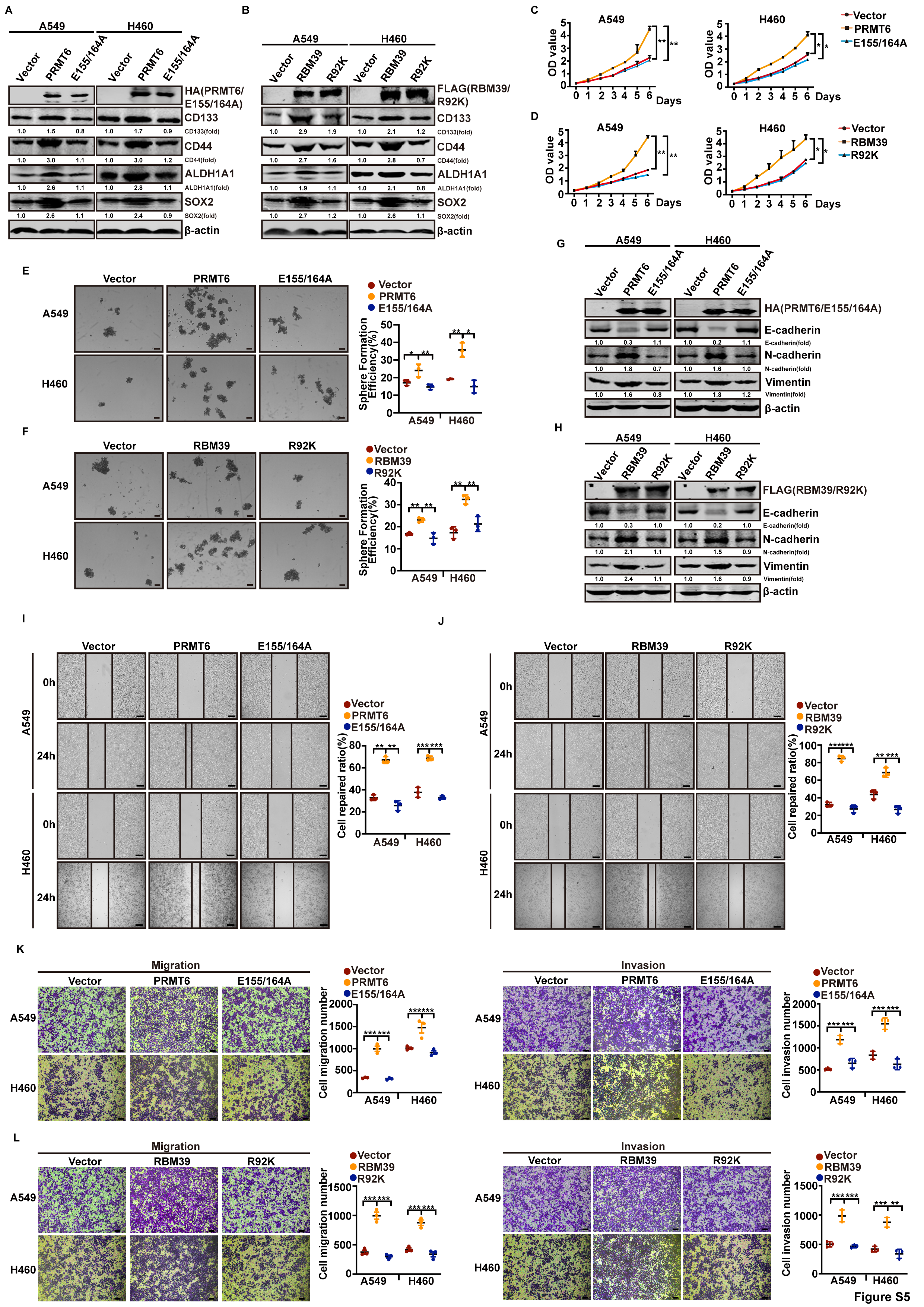

Supplement: S5 Fig — (A and B) A549 and H460 cells transfected with empty vector, PRMT6, or E155/164A, or empty vector, RBM39, or R92K, respectively, and the protein levels of stem-related markers were detected by western blotting. (C and D) MTT assays were performed to test the cell growth ability in the cells above. (E and F) Sphere formation assays were performed in indicated cell lines. Bars = 200 μm. Cells with RBM39 and PRMT6 overexpression increased the numbers of sphere-forming. (G and H) A549 and H460 cells were transfected with empty vector, PRMT6 or E155/164A, RBM39, or R92K. The protein levels of N-cadherin, vimentin, and E-cadherin in the indicated cells above were detected by western blotting. (I and J) The scratch wound healing assay measured the motile ability of the indicated cells above. Bars = 100 μm. (K and L) Transwell assays were performed to test the migratory and invasive potential of the indicated cells above, Bars = 200 μm. Data calculate the mean ± SD (n = 3). *p < 0.05, **p < 0.01, ***p < 0.001. Statistical analysis was calculated using the one-way ANOVA. The underlying data for S5C–S5F and S5I–S5L Fig can be found in S3 Data. (TIF) [file pbio.3002846.s005.tif]

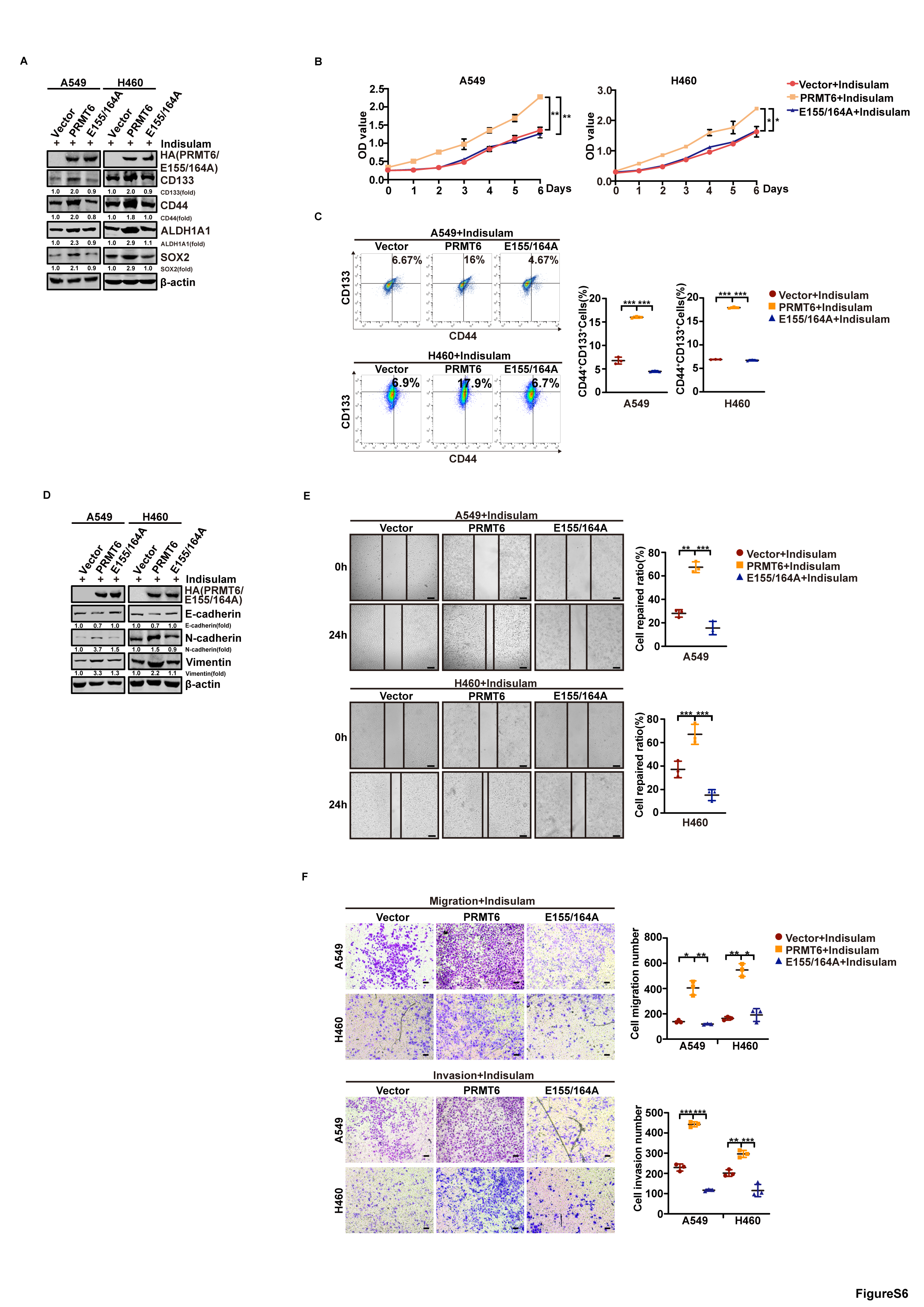

Supplement: S6 Fig — (A) A549 and H460 cells transfected with empty vector, PRMT6, or E155/164A, respectively, were treated with Indisulam, and the protein levels of stem-related markers were detected by western blotting. (B) MTT assays were performed to test the cell growth ability in the cells above treated with Indisulam. (C) The proportion of CD44+ CD133+ cells in indicated groups was analyzed using flow cytometry. (D) A549 and H460 cells were transfected with empty vector, PRMT6 or E155/164A with Indisulam treatment. The protein levels of N-cadherin, vimentin, and E-cadherin in the indicated cells above were detected by western blotting. (E) The scratch wound healing assay measured the motile ability of the indicated cells above. Bars = 100 μm. (F) Transwell assays were performed to test the migratory and invasive potential of the indicated cells above, Bars = 200 μm. Data calculates the mean ± SD (n = 3). *p < 0.05, **p < 0.01, ***p < 0.001. Statistical analysis was calculated using the one-way ANOVA. The underlying data for S6B–S6C and S6E–S6F Fig can be found in S3 Data. (TIF) [file pbio.3002846.s006.tif]

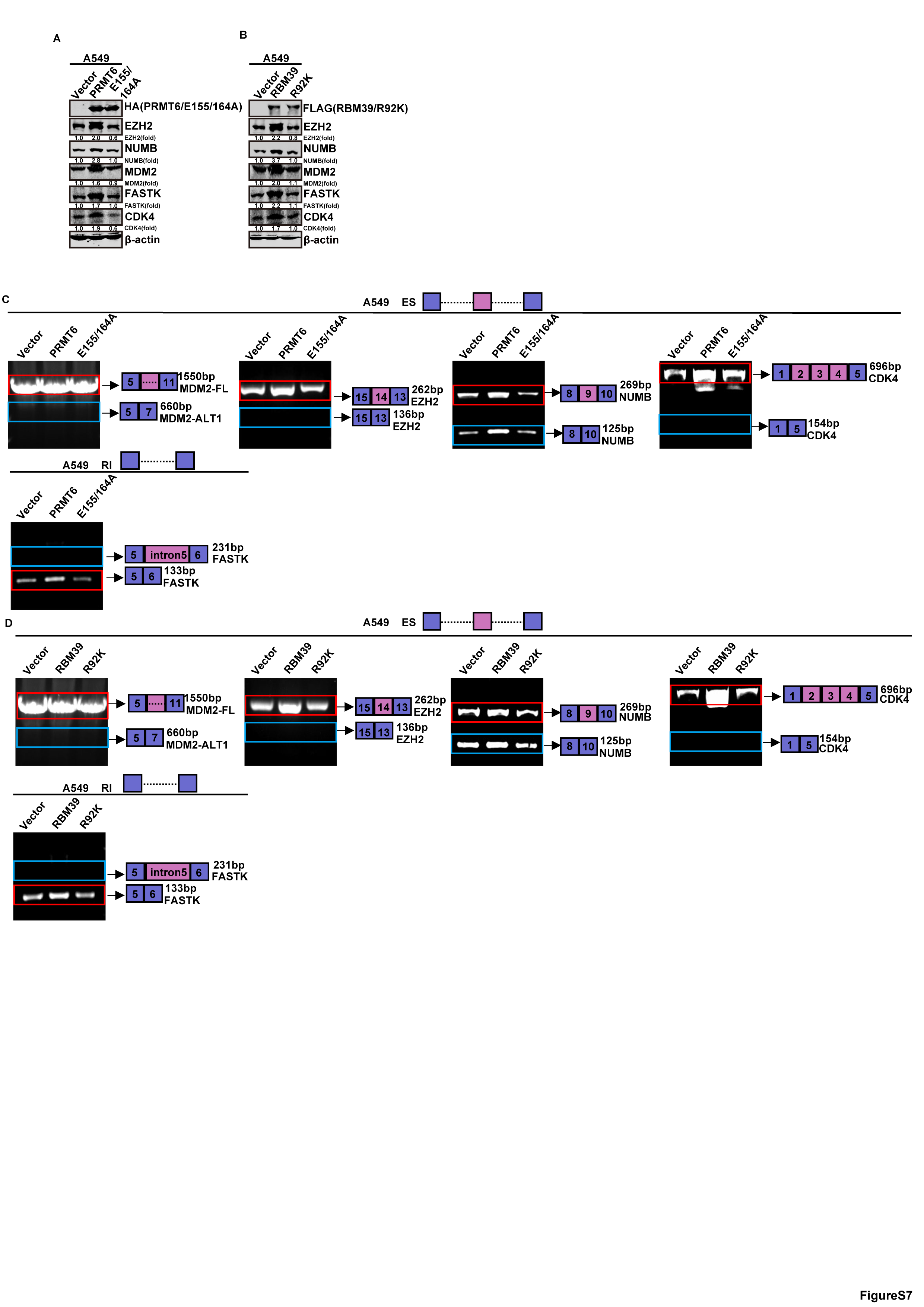

Supplement: S7 Fig — (A and B) Western blotting assessment of the expression of indicated molecules that undergo aberrant splicing. (A) Western blotting assessment of expression of indicated molecules that undergo aberrant splicing in A549 cells transfected with HA-PRMT6 or HA-E155/164A. (B) Western blotting assessment of the expression of indicated molecules that undergo aberrant splicing in A549 with stable expression of FLAG-RBM39 or FLAG-R92K. (C) Reverse transcription PCR validates mis-splicing events induced by transfection of PRMT6 or E155/E164A in A549 cells. PCR products were loaded into 1%–2% agarose gel with ethidium bromide for electrophoresis. Red color indicated the predicted molecular weight of PCR products. Blue color indicated the mis-spliced PCR products. (D) Reverse transcription PCR validates mis-splicing events induced by expression of RBM39 or R92K in A549 cells. (TIF) [file pbio.3002846.s007.tif]

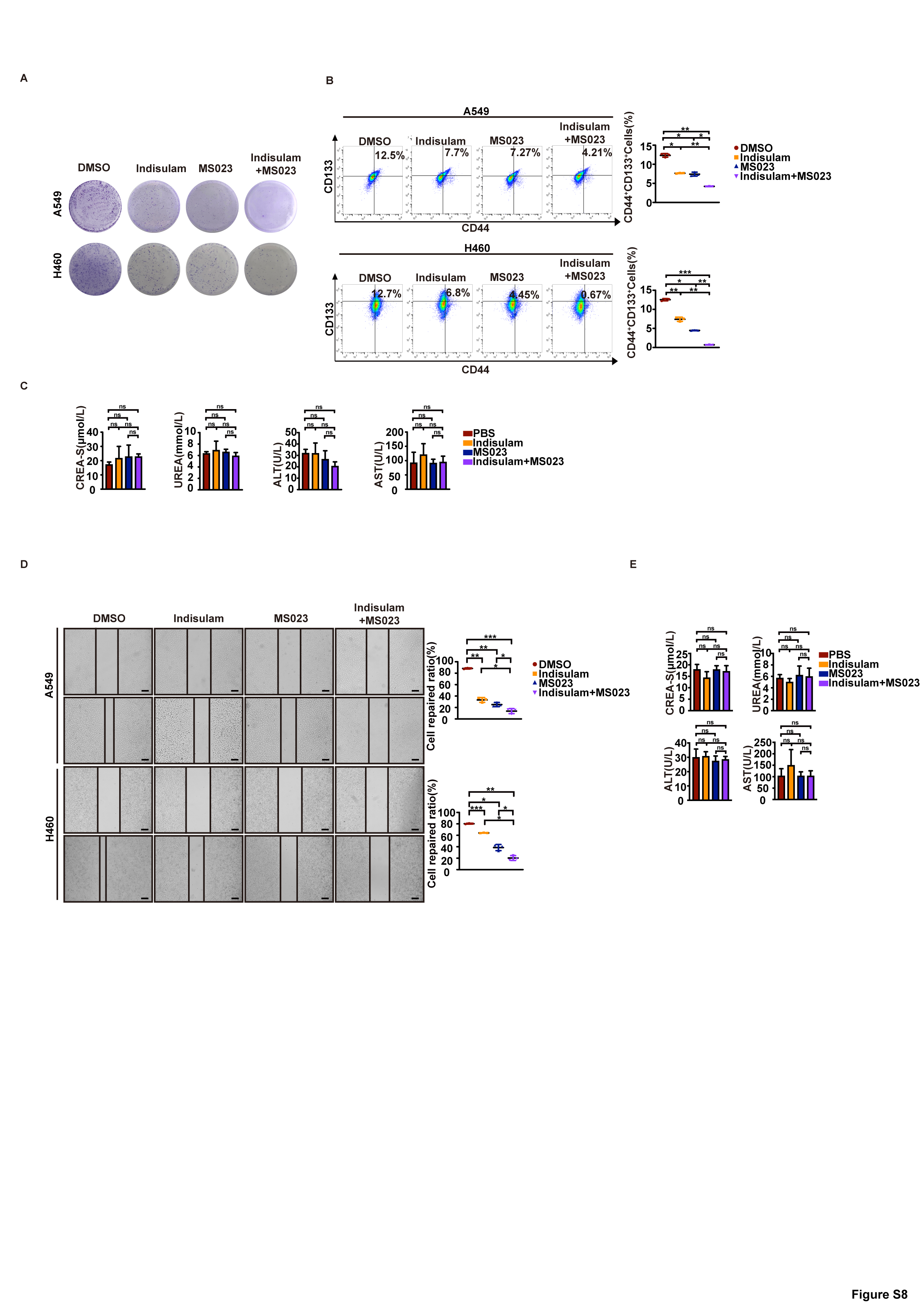

Supplement: S8 Fig — (A) Colony formation assay was performed to assess the clonogenic ability of A549 and H460 cells treated with DMSO, Indisulam, MS023, or Indisulam plus MS023. (B) Flow cytometry was used to quantify the CD44+ CD133+ cancer stem-like cell populations in the indicated treatment group. Bars = 200 μm. (C) Serum levels of ALT, AST, UREA, and CREA were measured in nude mice bearing subcutaneous xenografts under different treatment conditions. (D) Scratch wound healing assay was used to evaluate the migratory ability of A549 and H460 cells after treatment with DMSO, Indisulam, MS023, or their combination. Bars = 100 μm. (E) Serum levels of ALT, AST, UREA, and CREA were also assessed in a metastatic mouse model following the same treatments. *p < 0.05, **p < 0.01, ***p < 0.001. Statistical analysis was calculated using the one-way ANOVA. The underlying data for S8B–S8E Fig can be found in S3 Data. (TIF) [file pbio.3002846.s008.tif]

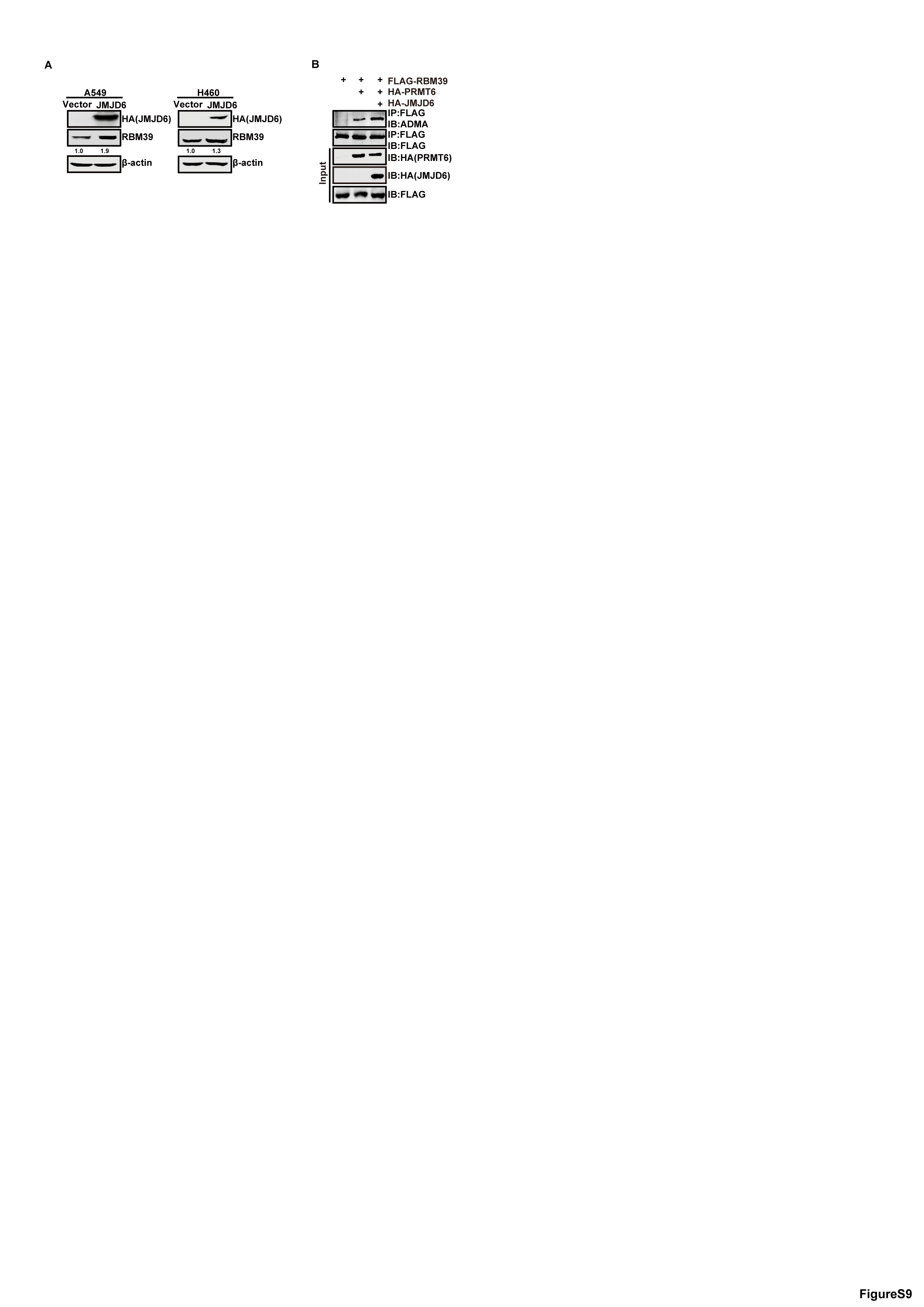

Supplement: S9 Fig — (A) JMJD6 overexpression increases the protein level of RBM39. (B) JMJD6 overexpression increases ADMA levels in vivo. FLAG-RBM39 and HA-PRMT6/JMJD6 were individually co-transfected into HEK293T cells. Total cell lysates were then immunoprecipitated using anti-FLAG antibodies and analyzed by western blotting. (TIF) [file pbio.3002846.s009.tif]
