# Supplementary material for: Methylation of RBM39 by PRMT6 enhances resistance to Indisulam in non-small cell lung cancer by promoting alternative splicing of proto-oncogenes
Source: PLoS Biol. 2025 Jun 4;23(6):e3002846. doi: 10.1371/journal.pbio.3002846 (PMC12142651; doi:10.1371/journal.pbio.3002846)
Supplement: S1 Table — (DOCX) [file pbio.3002846.s010.docx]

**SUPPLEMENTARY INFORMATION**

**Methylation of RBM39 by PRMT6 enhances resistance to Indisulam in non-small cell lung cancer by promoting alternative splicing of proto-oncogenes**

Tongjia Zhang^1^, Shujie Wang^1^, Yue Zhou^1^, Zitao Jiao^1^, Kejia Lu^1^, Xinyi Liu^1^, Hui Li^1^, Wei Jiang^1^, Xiaowei Zhang^1^, *

^1^Department of Biochemistry and Molecular Biology, School of Basic Medical Sciences, Beijing Key Laboratory of Protein Posttranslational Modifications and Cell Function, Peking University Health Science Center, Beijing 100191, P. R. China.

***Corresponding author:** Xiaowei Zhang, Department of Biochemistry and Molecular Biology, Peking University Health Science Center, Haidian District, Xueyuan Road 38, Beijing 100191, P. R. China. Telephone: +8610 8280 2418. Email: [xiaoweizhang@bjmu.edu.cn](mailto:xiaoweizhang@bjmu.edu.cn).

**Supplementary tables**

**Supplementary Table 1. Key Resource Table**

| REAGENT or RESOURCE | SOURCE | IDENTIFIER |
| --- | --- | --- |
| **Antibodies** | | |
| RBM39 (1:2000) | Proteintech | Cat# 21339-1-AP |
| PRMT6 (1:2000) | Proteintech | Cat# 15395-1-AP |
| CD44 (1:1000) | BioLegend | Cat# 397502 |
| CD133 (1:1000) | BioLegend | Cat# 397902 |
| ALDH1A1 (1:1000) | BioLegend | Cat# 861901 |
| SOX2 (1:1000) | BioLegend | Cat# 656102 |
| Vimentin (1:1000) | Santa Cruz | Cat# sc-373717 |
| E-cadherin (1:1000) | Santa Cruz | Cat# sc-8426 |
| N-cadherin (1:1000) | Santa Cruz | Cat# sc-8424 |
| FLAG (1:3000) | Sigma-Aldrich | Cat# F1804 |
| HA (1:1000) | BioLegend | Cat# 614852 |
| Mono-Methyl Arginine (1:1000) | CST | Cat# 8015 |
| Asymmetric Di-Methyl Arginine (1:1000) | CST | Cat# 13522 |
| β-actin (1:10000) | ABclonal | Cat# AC026 |
| DyLight 800 Conjugated anti-Rabbit IgG  (1:3000) | EarthOx | Cat# E032820 |
| DyLight 800 Conjugated anti-Mouse IgG  (1;3000) | EarthOx | Cat# E032810 |
| Goat Anti-Mouse IgG (H+L), Dylight549  (1;3000) | EarthOx | Cat# E032310 |
| Goat Anti- Rabbit IgG (H+L), Dylight488  (1;3000) | EarthOx | Cat# E032210 |
| APC anti-human CD44(1:20 | BioLegend | Cat# 338806 |
| PE anti-human CD133(1:20) | BioLegend | Cat# 397904 |
| Ubiquitin | MBL | Cat# D058-3 |
| Anti-Ki67 (1:100) | Affinity Biosciences | Cat# AF0198 |
| His (1:1000) | Proteintech | Cat# 66005-1-Ig |
| GST (1:1000) | Proteintech | Cat# 10000-0-AP |
| DCAF15 (1:1000) | Proteintech | Cat# 29362-1-AP |
| **Bacterial and Virus strains** | | |
| Trelief 5α Chemically Competent Cell | TSINGKE | Cat# TSC-C01 |
| BL21(DE3) Chemically Competent Cell | TSINGKE | Cat# TSC-E01 |
| **Chemicals, peptides, and recombinant proteins** | | |
| Fetal Bovine Serum | TransGen Biotech | Cat# FS301-02 |
| DMEM, high glucose | EallBio | Cat# 03.1002c |
| DMEM/F-12 | EallBio | Cat# 03.2001C-PS |
| RPMI1640 | EallBio | Cat# 03.4007C |
| Ham’s F-12K | EallBio | Cat# 03.6003C |
| Penicillin-Streptomycin | EallBio | Cat# 03.12001A |
| Trypsin-EDTA 2.5% | macgene | Cat# CC012.100 |
| Puromycin, dihydrochloride | macgene | Cat# MA009 |
| Polybrene | macgene | Cat# MC032 |
| TurboFect transfection reagent | Thermo Scientific | Cat# R0533 |
| MG132 | Innochem | Cat# 133407-82-6 |
| SAM | Sigma | Cat# A7007 |
| IPTG | Yuanye Bio-Technology | Cat# S11086 |
| MTT | Sigma | Cat# 475989 |
| Indisulam | MedChemExpress | Cat# HY-13650 |
| MS023 | MedChemExpress | Cat# HY-19615 |
| Protease Inhibitor Cocktail | Bimake | Cat# B14002 |
| DMSO | GPC | Cat# AW068 |
| Cycloheximide | Sigma | Cat# C7698 |
| Protein A sepharose CL-4B | Cytiva | Cat# 17078001 |
| Glutathione Sepharos4B | pharmacia | Cat# 17-0756-01 |
| HisPur Cobalt Resin | Invitrogen | Cat# 89964 |
| TRIS | Biorigin | Cat# Bn20006 |
| Glycine | Biorigin | Cat# BN20001 |
| TEMED | Biosharp | Cat# T8090 |
| Acr-Bis | Servicebio | Cat# G2004 |
| Ammonium persulphate | MREDA | Cat# M214390 |
| SDS | Servicebio | Cat# GC204005 |
| NP-40 | Applygen | Cat# A1008 |
| Agarose | biowest | Cat# 111860 |
| Tween-20 | Solarbio | Cat# T8220 |
| Prestained Protein Marker III | Servicebio | Cat# G2058 |
| N-Ethylmaleimide | EKASION | Cat# E3876-5G |
| AceQ Universal SYBR qPCR Master Mix | Vazyme | Cat# Q511-02 |
| HiScript II QRT SuperMix for qPCR | Vazyme | Cat# R223-01 |
| DL2000 Plus DNA Marker | Vazyme | Cat# MD101-02 |
| T4 DNA Ligase | Thermo | Cat# EL0011 |
| EGF | Gibco | Cat# PHG0311L |
| bFGF | Gibco | Cat# 13256-029 |
| B-27 | Gibco | Cat# 17504044 |
| RIPA | Thermo | Cat#89900 |
| LIF | Bioss | Cat# Bs-1058R |
| **Critical commercial assays** | | |
| RNAsimple Total RNA Kit | TIANGEN | Cat# DP419 |
| EndoFree Plasmid Midi Kit | CWBIO | Cat# CW21055 |
| Gel Extraction Kit | CWBIO | Cat# CW2302M |
| Pierce BCA Protein Assay Kit | Invitrogen | Cat# 23225 |
| RevertAid First Strand cDNA Synthesis Kit | Thermo | Cat# K1622 |
| Phusion High-Fidelity DNA Polymerase | Thermo | Cat# F-530L |
| **Deposited data** | | |
| **Experimental Models: Cell Lines** | | |
| HEK293T | ATCC | Cat# CRL-3216 |
| A549 | ATCC | Cat# CRM-CCL-185 |
| H460 | ATCC | Cat# HTB-177 |
| **Experimental models: Organisms** | | |
| Lung cancer samples | Outdo Biotech | Cat# HLugA030PG04-2 |
| Mouse | Peking University Health Science Center | N/A |
| **Oligonucleotides** | | |
| Human RBM39-qRT-PCR  Forward: CAATGCTTGAGGCTCCTTACA  Reverse:  TCCGTTCCTTACTTTTGCTTCTC | TSINGKE | N/A |
| Human β-actin -qRT-PCR  Forward: CAAATGCTTCTAGGCGGACT  Reverse:  TGCTGTCACCTTCACCGTTC | TSINGKE | N/A |
| Human EZH2-AS  Forward: CACACTGCAGAAAGATACAGCT  Reverse:  CAAGTAAGACAGAGGTCAGGGT | TSINGKE | N/A |
| Human MDM2-outside-AS  Forward: CTGGGGAGTCTTGAGGGACC  Reverse:  CAGGTTGTCTAAATTCCTAG | TSINGKE | N/A |
| Human MDM2-interna-AS  Forward:  CGCGAAAACCCCGGGCAGGCAAATGTGCA  Reverse:  CTCTTATAGACAGGTCAACTAG | TSINGKE | N/A |
| Human Numb-AS  Forward:  ACCTTGGCCATGTAGAAGTTGAT  Reverse:  AGTGAGGGAATGTCTGCTGC | TSINGKE | N/A |
| Human CDK4-AS  Forward:  GTGTATGGGGCCGTAGGAAC  Reverse:  CCAACACTCCACATGTCCAC | TSINGKE | N/A |
| Human FASTK-AS  Forward:  CATCTTGATGTCACTGTGCCA  Reverse:  CAGCAGGGAGAGGTAGCG | TSINGKE | N/A |
| shRBM39-1: GCTTCGAGTGCTAGTTCATTT | TSINGKE | N/A |
| shRBM39-2: AAACATGTTAGAGAGTTGG | TSINGKE | N/A |
| sgRNA targeting RBM39:  GCTTGAGGCTCCTTACAAGA | TSINGKE | N/A |
| sgRNA targeting PRMT6:  GCGAGTGCTACTCGGACGTTT | TSINGKE | N/A |
| **Recombinant DNA** | | |
| pLVX-IRES-Puro-RBM39 | This study | N/A |
| pLVX-IRES-Puro-R92K | This study | N/A |
| pLVX-IRES-Puro-PRMT6 | This study | N/A |
| pCDNA3-HA-PRMT6 | This study | N/A |
| pCDNA3-HA-E155/164A | This study | N/A |
| pCDNA3-HA-PRMT1 | This study | N/A |
| pCDNA3-HA-PRMT3 | This study | N/A |
| CMV10-3XFLAG-RBM39 | This study | N/A |
| CMV10-3XFLAG-R92K | This study | N/A |
| CMV10-3XFLAG-R109K | This study | N/A |
| CMV10-3XFLAG-R267K | This study | N/A |
| pSpCas9(BB)−2A-puro-RBM39 | This study | N/A |
| pSpCas9(BB)−2A-puro-PRMT6 | This study | N/A |
| pET-28(a)-RBM39 | This study | N/A |
| pET-28(a)-PRMT6 | This study | N/A |
| pGEX-4T-1-RBM39 | This study | N/A |
| pGEX-4T-1-PRMT6 | This study | N/A |
| **Software and algorithms** | | |
| Odyssey | LI-COR Bioscience | https://www.licor.com |
| Image J 1.54i | Wayne Rasband | http://imagej.net/software/imagej |
| ZEN | ZEISS | http://zeiss.com.cn |
| FlowJo V.10.0. | BD | https://www.flowjo.com |
| R version 4.3.3 | R Core Team | https://www.rproject.org |
