# Supplementary material for: Methylation of RBM39 by PRMT6 enhances resistance to Indisulam in non-small cell lung cancer by promoting alternative splicing of proto-oncogenes
Source: PLoS Biol. 2025 Jun 4;23(6):e3002846. doi: 10.1371/journal.pbio.3002846 (PMC12142651; doi:10.1371/journal.pbio.3002846)
Supplement: S1 Raw Images — (PDF) [file pbio.3002846.s014.pdf]

Figure1

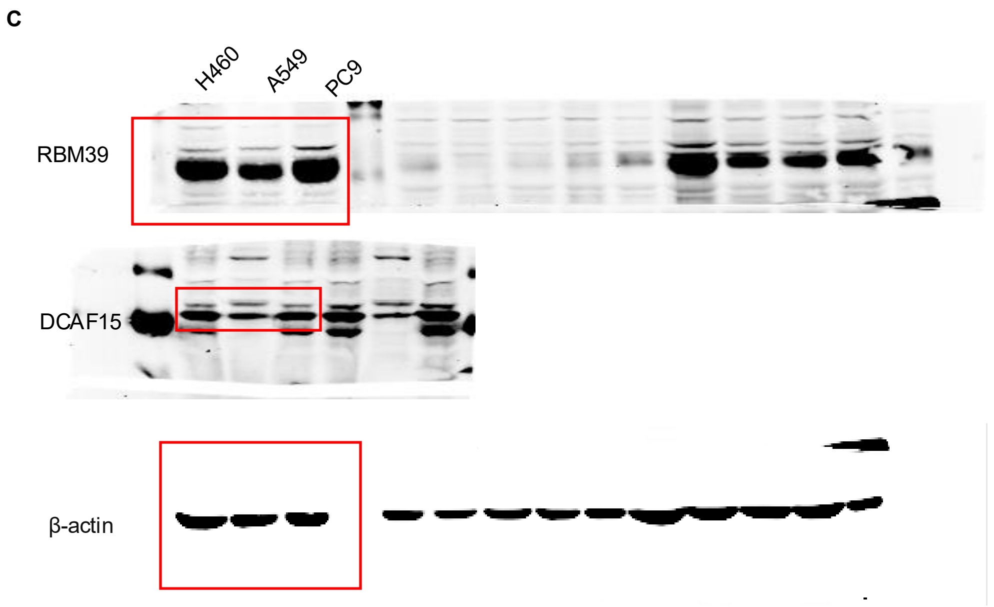

Figure1

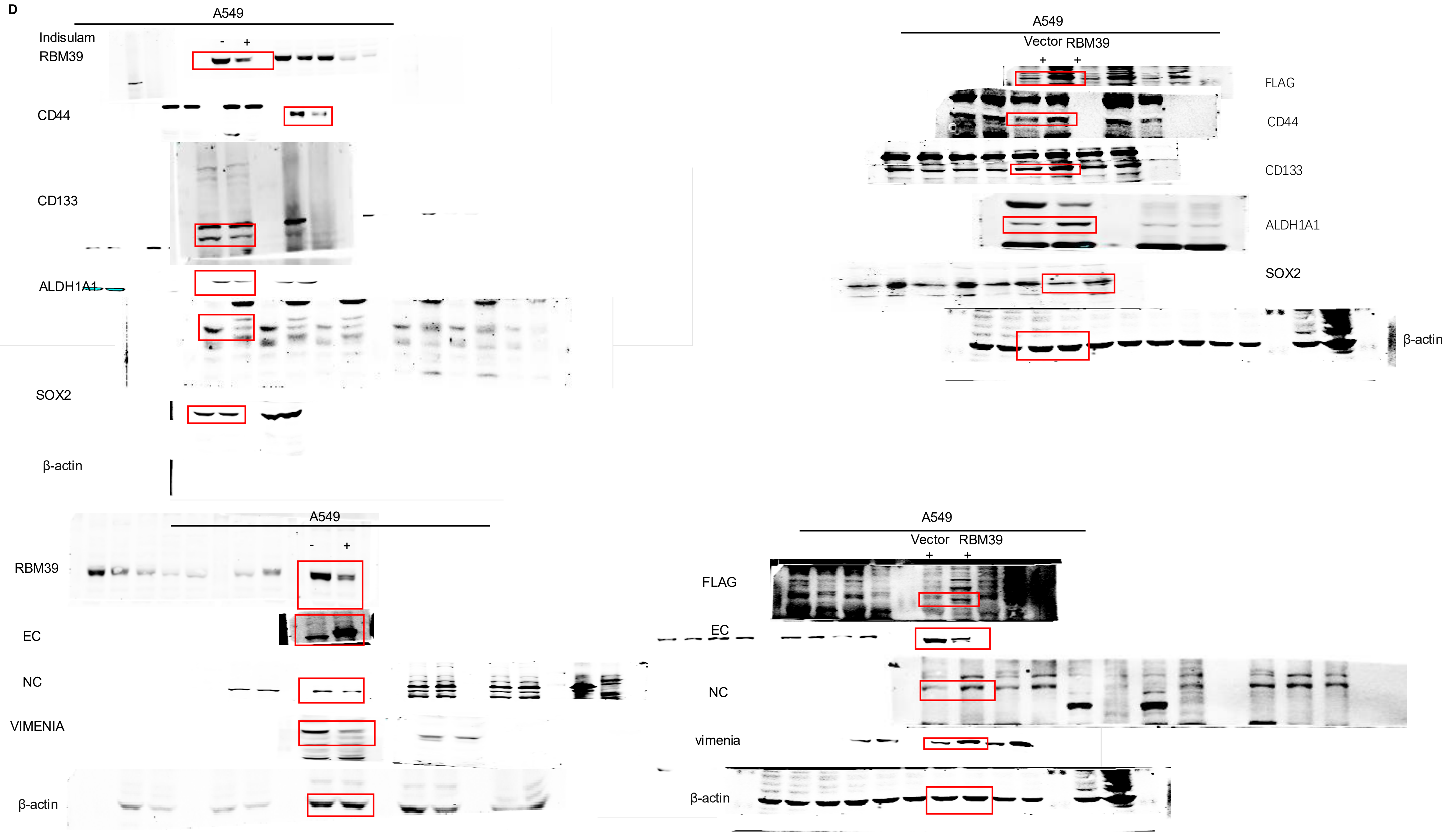
D
A549
Indisulam
RBM39
- +
CD44
CD133
ALDH1A1
SOX2
β-actin
A549
Vector RBM39
+ +
FLAG
CD44
CD133
ALDH1A1
SOX2
β-actin
A549
- +
RBM39
EC
NC
VIMENIA
β-actin
A549
Vector RBM39
+ +
FLAG
EC
NC
vimenia
β-actin

Figure1

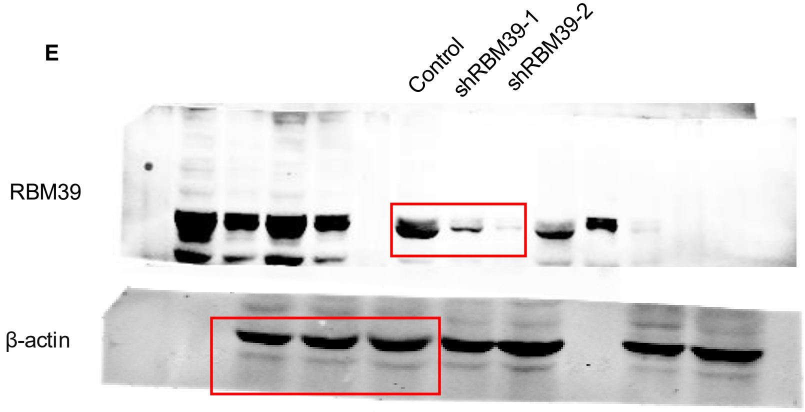

Figure1

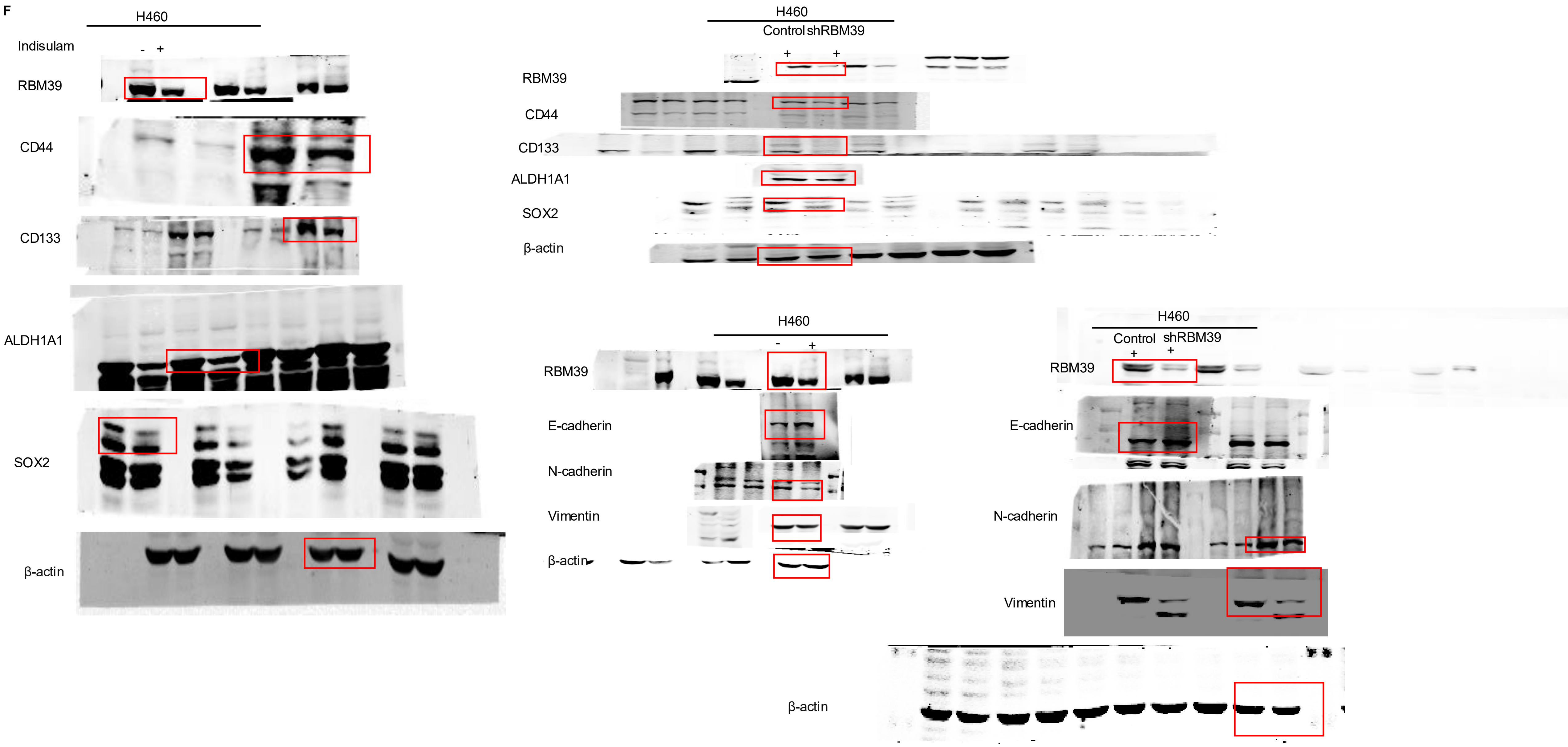
F
H460
Indisulam
-
+
RBM39
CD44
CD133
ALDH1A1
SOX2
β-actin
H460
Control shRBM39
+
+
RBM39
CD44
CD133
ALDH1A1
SOX2
β-actin
H460
-
+
RBM39
E-cadherin
N-cadherin
Vimentin
β-actin
H460
Control
shRBM39
+
+
RBM39
E-cadherin
N-cadherin
Vimentin
β-actin

Figure2

E

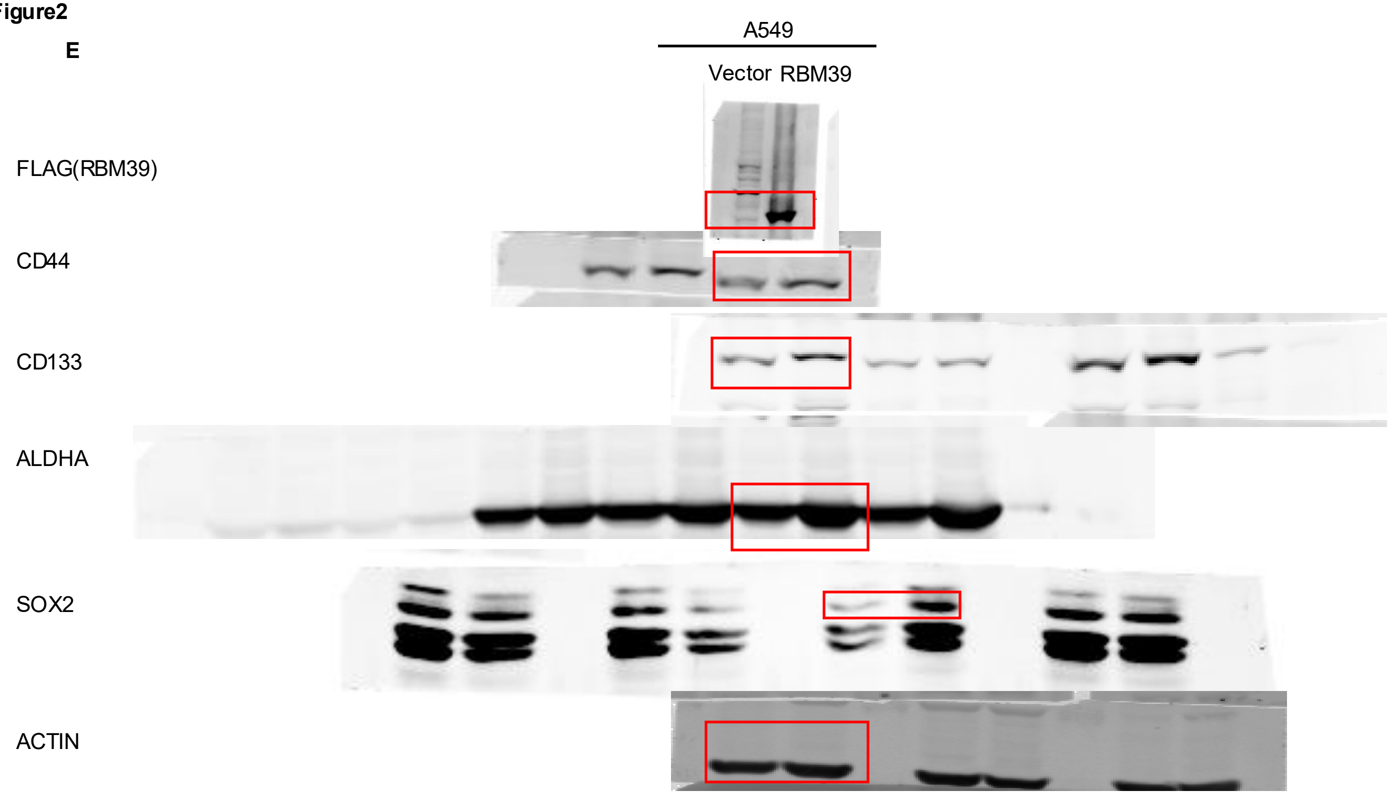
A549
Vector RBM39
FLAG(RBM39)
CD44
CD133
ALDHA
SOX2
ACTIN

F

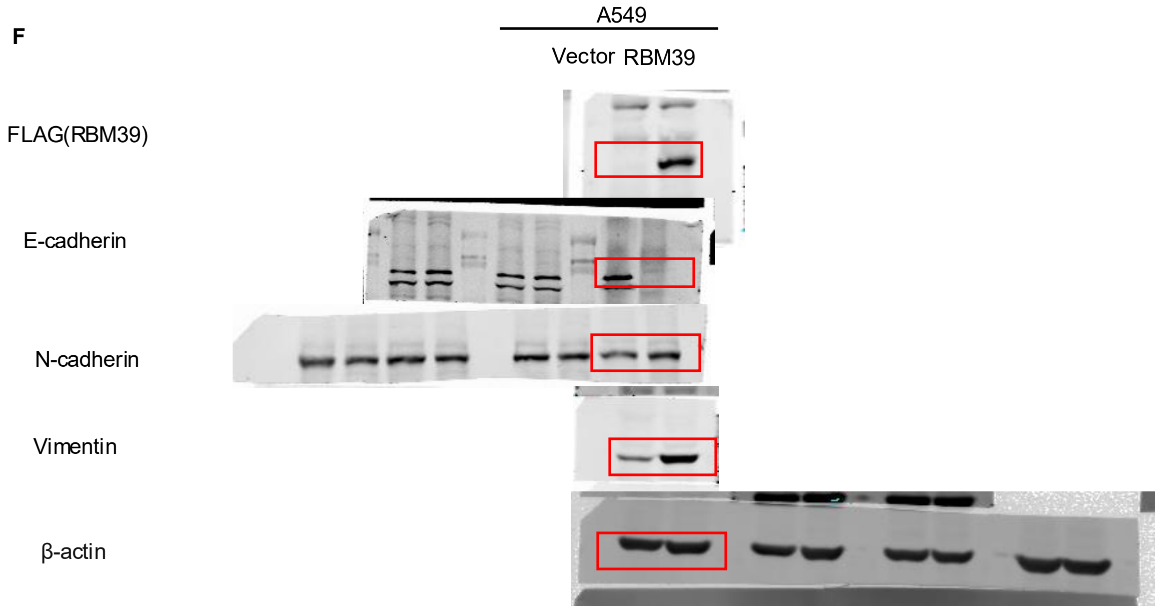
A549
Vector RBM39
FLAG(RBM39)
E-cadherin
N-cadherin
Vimentin
β-actin

Figure2

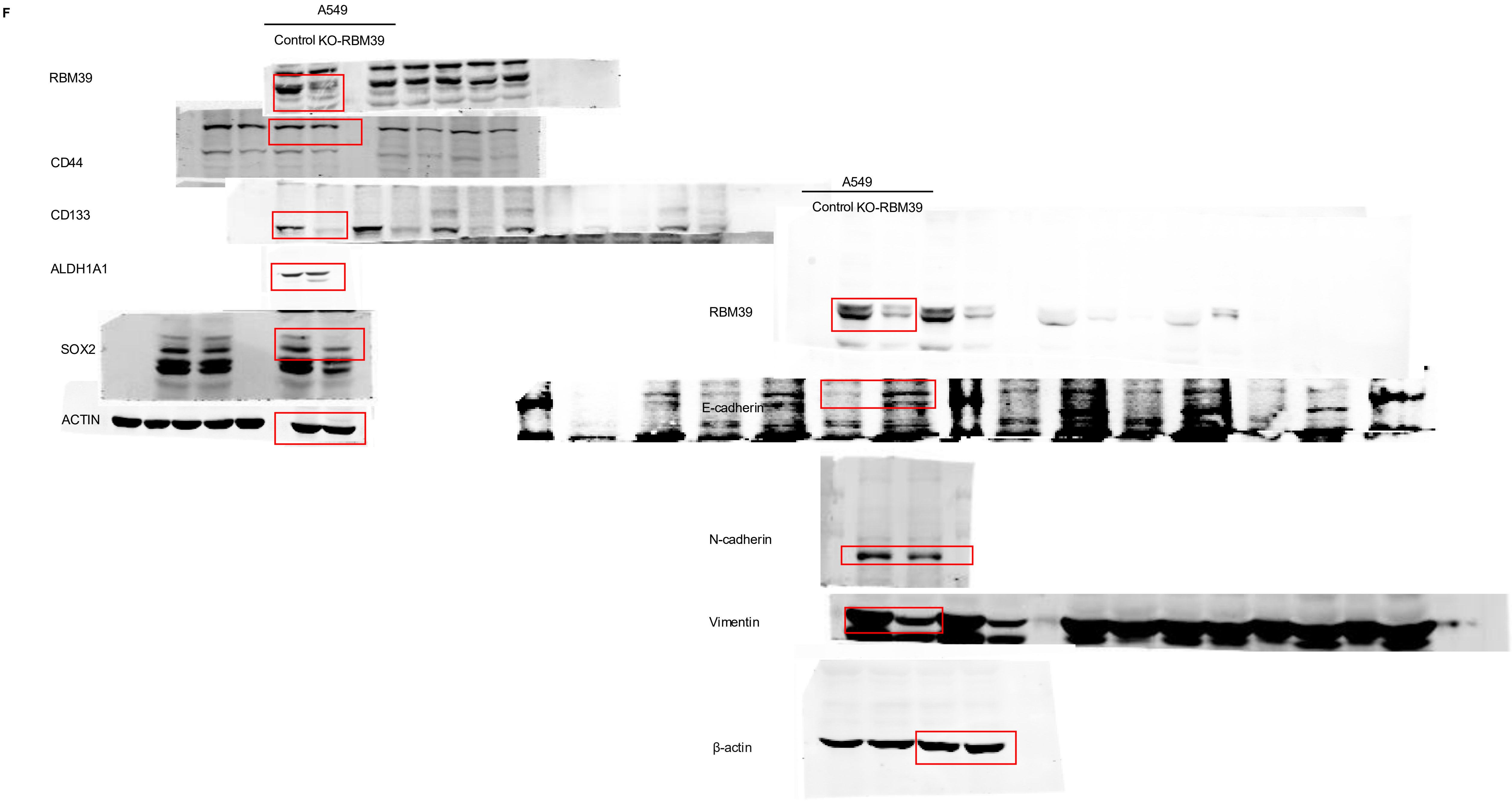
F
A549
Control KO-RBM39
RBM39
CD44
CD133
ALDH1A1
SOX2
ACTIN
A549
Control KO-RBM39
RBM39
E-cadherin
N-cadherin
Vimentin
β-actin

Figure3

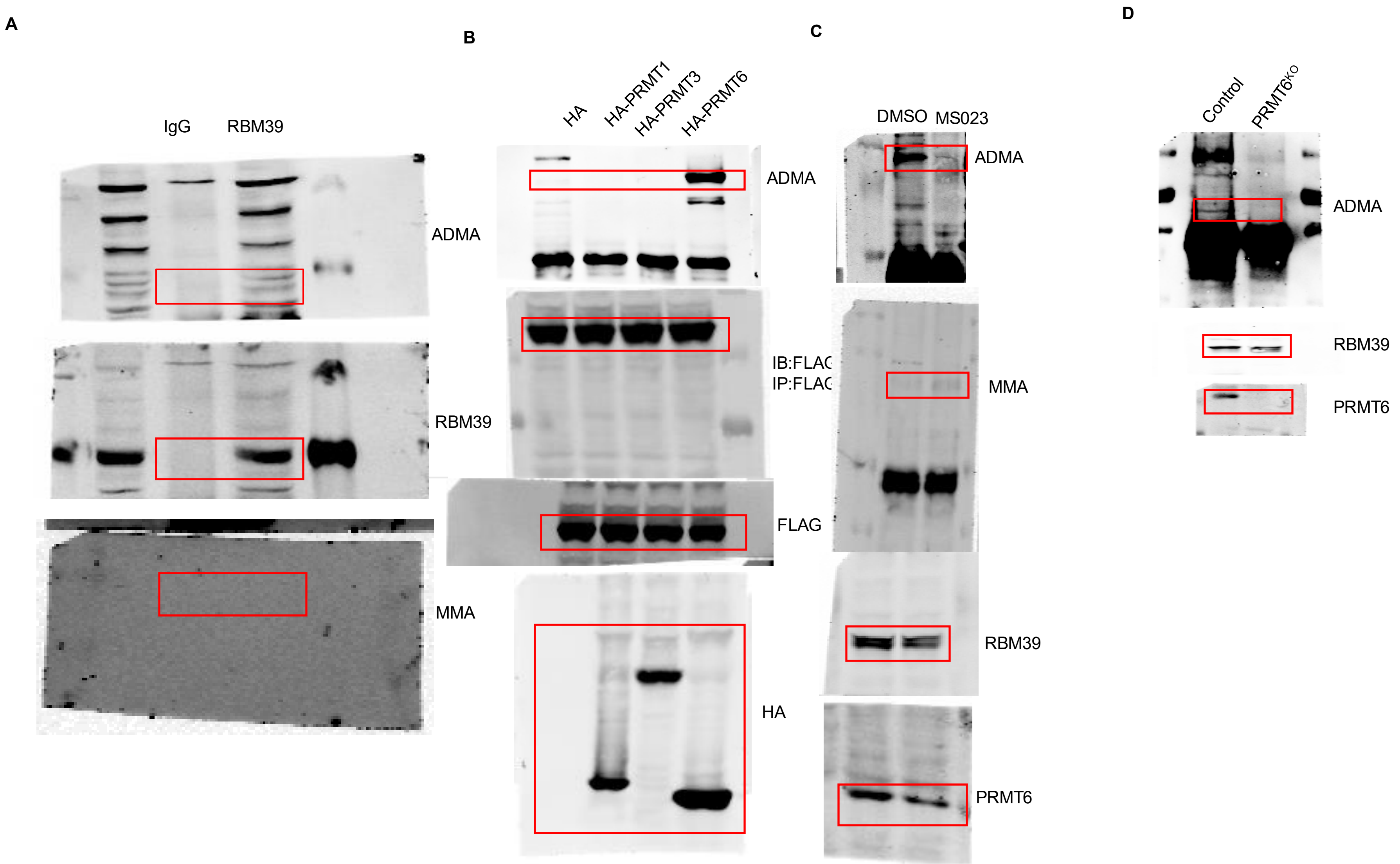
A
IgG
RBM39
ADMA
RBM39
MMA
B
HA
HA-PRMT1
HA-PRMT3
HA-PRMT6
ADMA
IB:FLAG
IP:FLAG
FLAG
HA
C
DMSO
MS023
ADMA
MMA
RBM39
PRMT6
D
Control
PRMT6KO
ADMA
RBM39
PRMT6

Figure3

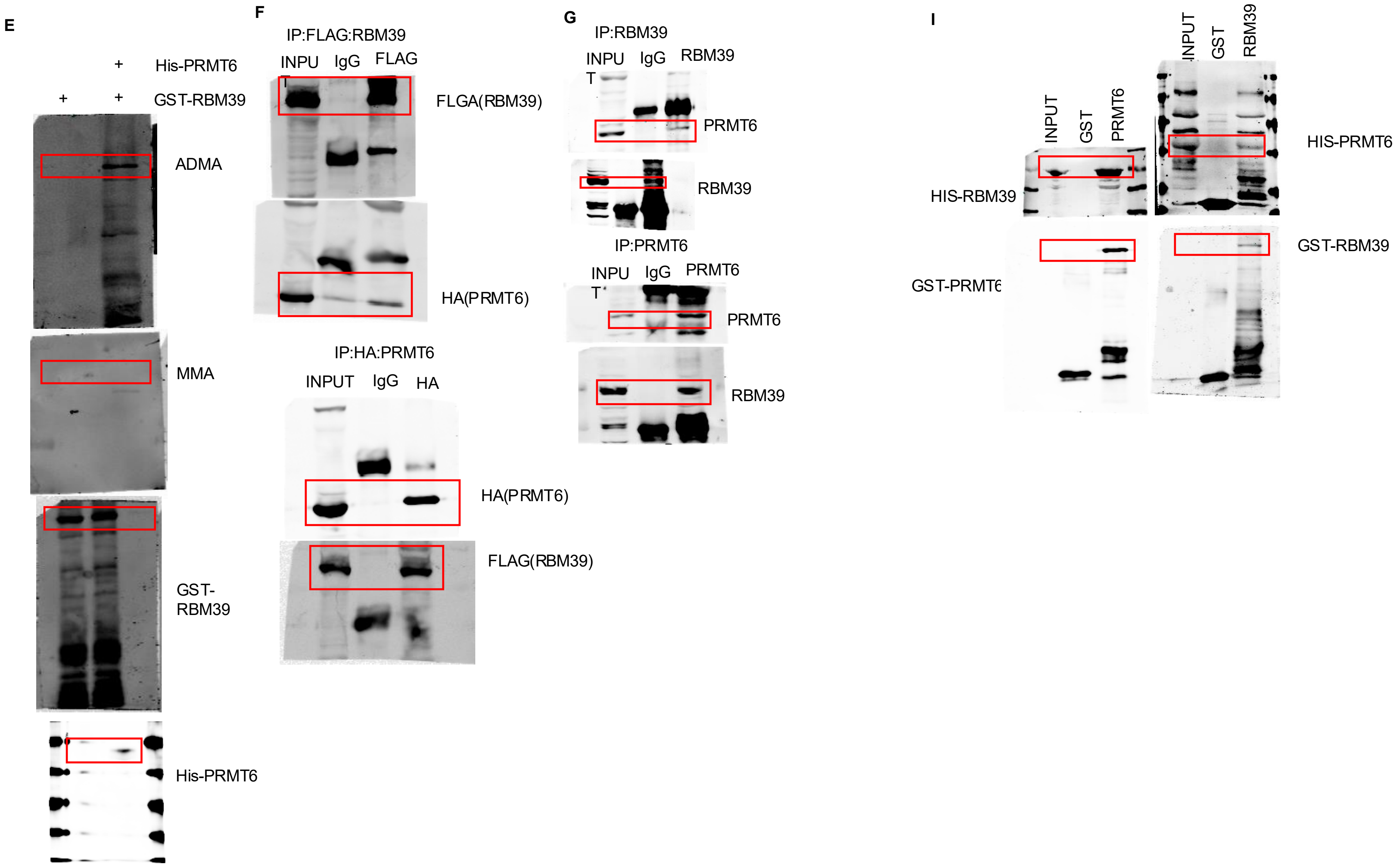
E
+
His-PRMT6
+
+
GST-RBM39
ADMA
MMA
GST-
RBM39
His-PRMT6
F
IP:FLAG:RBM39
INPU
T
IgG
FLAG
FLGA(RBM39)
HA(PRMT6)
IP:HA:PRMT6
INPUT
IgG
HA
HA(PRMT6)
FLAG(RBM39)
G
IP:RBM39
INPU
T
IgG
RBM39
PRMT6
RBM39
IP:PRMT6
INPU
T
IgG
PRMT6
PRMT6
RBM39
I
INPUT
GST
PRMT6
HIS-RBM39
GST-PRMT6
INPUT
GST
RBM39
HIS-PRMT6
GST-RBM39

Figure3

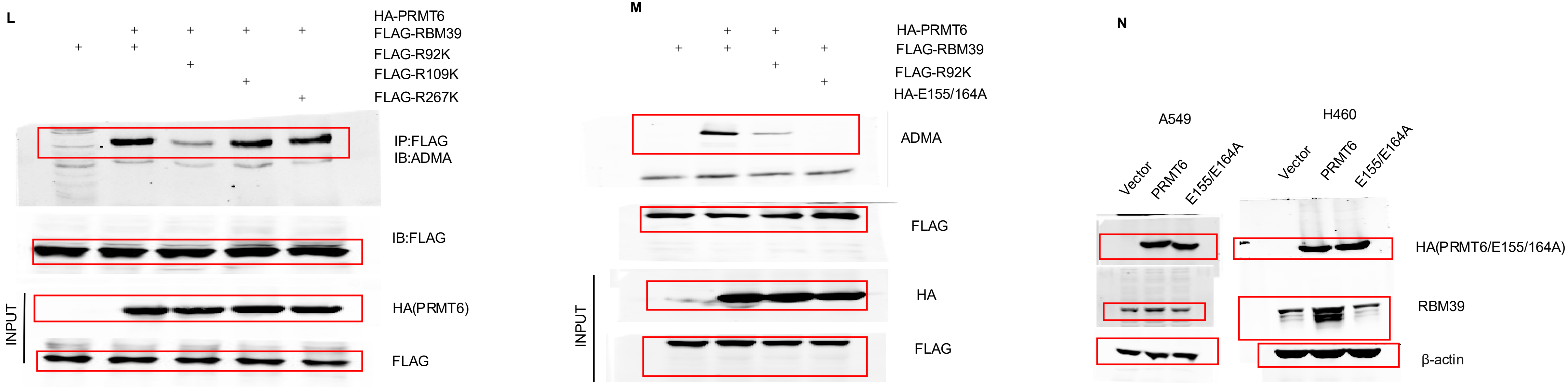
L
HA-PRMT6
FLAG-RBM39
FLAG-R92K
FLAG-R109K
FLAG-R267K
IP:FLAG
IB:ADMA
IB:FLAG
INPUT
HA(PRMT6)
FLAG
M
HA-PRMT6
FLAG-RBM39
FLAG-R92K
HA-E155/164A
ADMA
FLAG
INPUT
HA
FLAG
N
A549
H460
Vector
PRMT6
E155/E164A
Vector
PRMT6
E155/E164A
HA(PRMT6/E155/164A)
RBM39
β-actin

Figure4

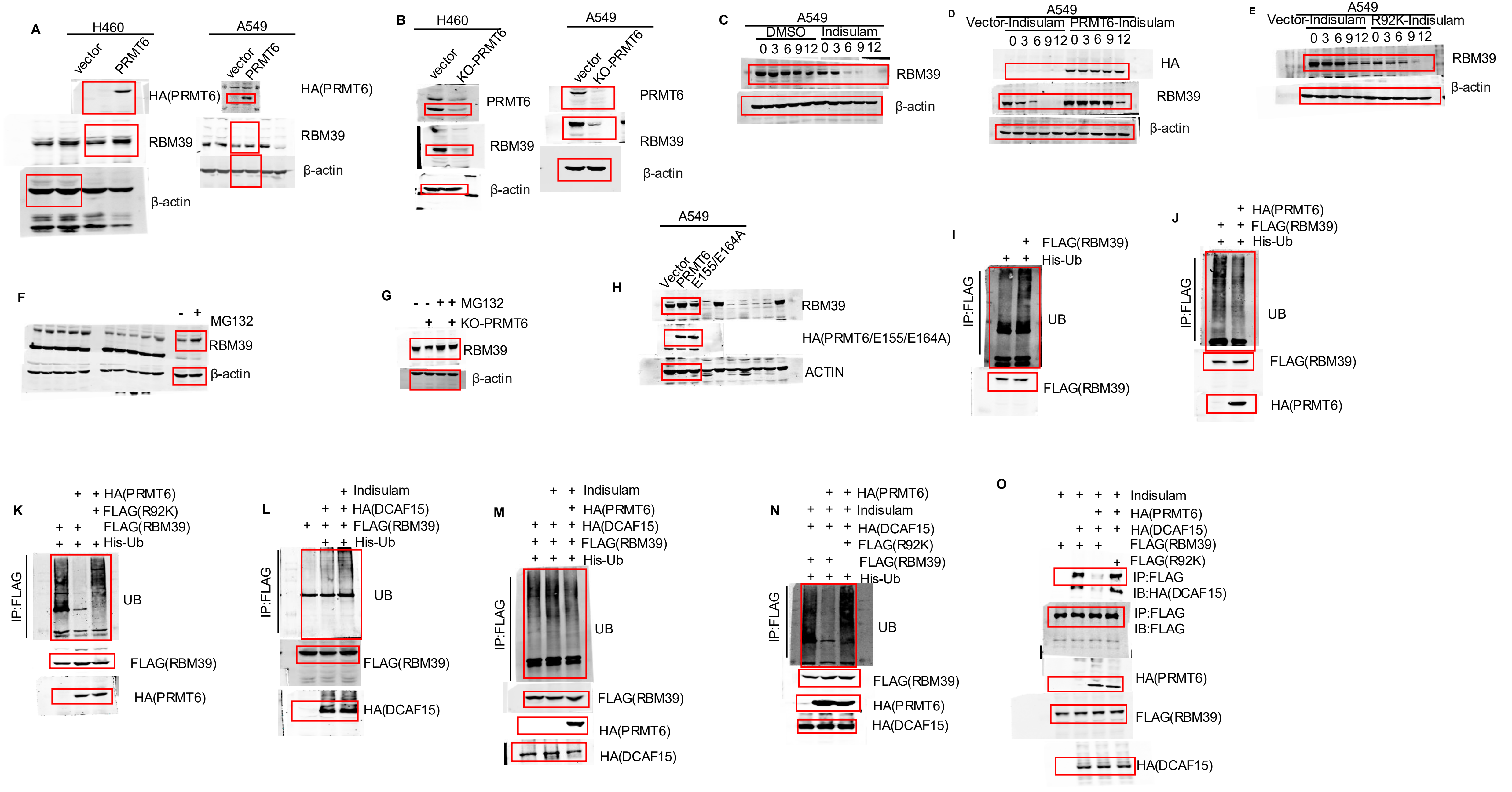
A
H460
A549
vector
PRMT6
HA(PRMT6)
RBM39
β-actin
B
KO-PRMT6
PRMT6
C
DMSO
Indisulam
0 3 6 9 12
D
Vector-Indisulam
PRMT6-Indisulam
HA
E
R92K-Indisulam
F
MG132
G
H
Vector
E155/E164A
HA(PRMT6/E155/E164A)
ACTIN
I
FLAG(RBM39)
His-Ub
IP:FLAG
UB
J
K
FLAG(R92K)
L
Indisulam
HA(DCAF15)
M
N
O
IP:FLAG
IB:HA(DCAF15)
IB:FLAG

Figure4

P

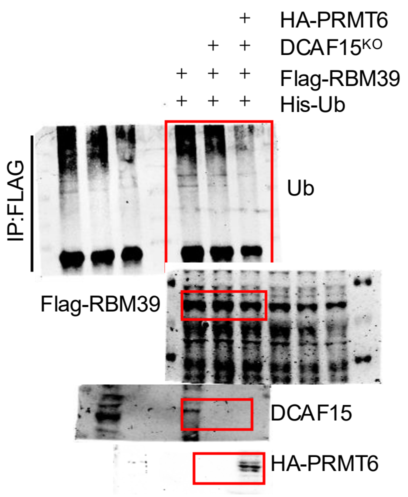
HA-PRMT6
DCAF15KO
Flag-RBM39
His-Ub
IP:FLAG
Ub
Flag-RBM39
DCAF15
HA-PRMT6

R

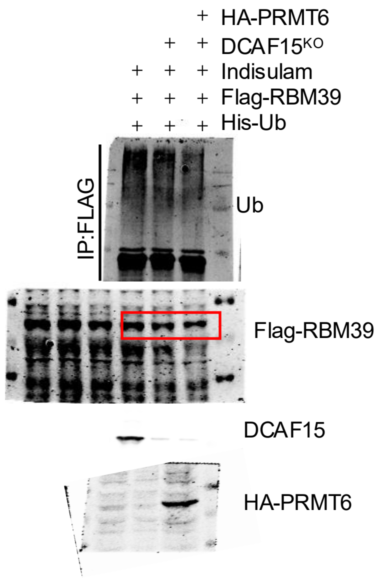
HA-PRMT6
DCAF15KO
Indisulam
Flag-RBM39
His-Ub
IP:FLAG
Ub
Flag-RBM39
DCAF15
HA-PRMT6

Figure5

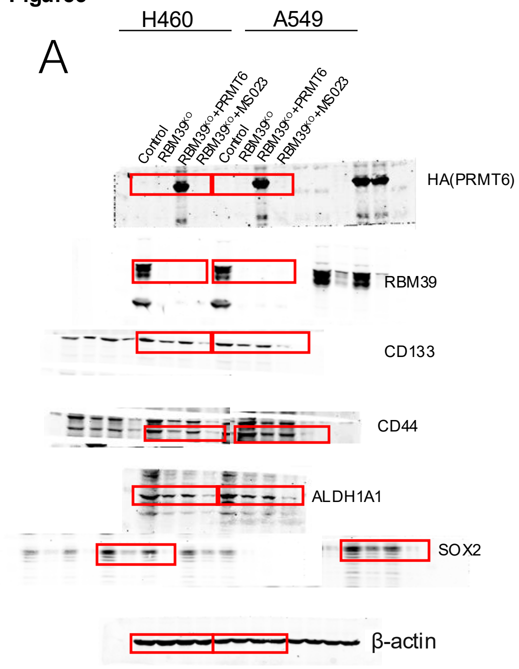
A
H460
A549
Control
RBM39^KO
RBM39^KO+PRMT6
RBM39^KO+MS023
HA(PRMT6)
RBM39
CD133
CD44
ALDH1A1
SOX2
β-actin

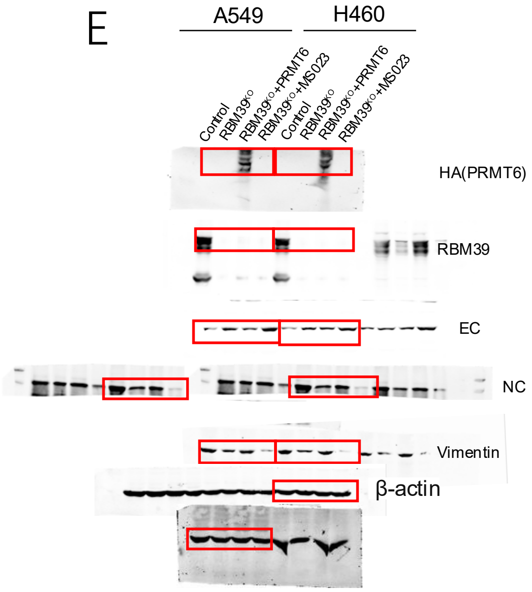
E
A549
H460
Control
RBM39^KO
RBM39^KO+PRMT6
RBM39^KO+MS023
HA(PRMT6)
RBM39
EC
NC
Vimentin
β-actin

Figure6

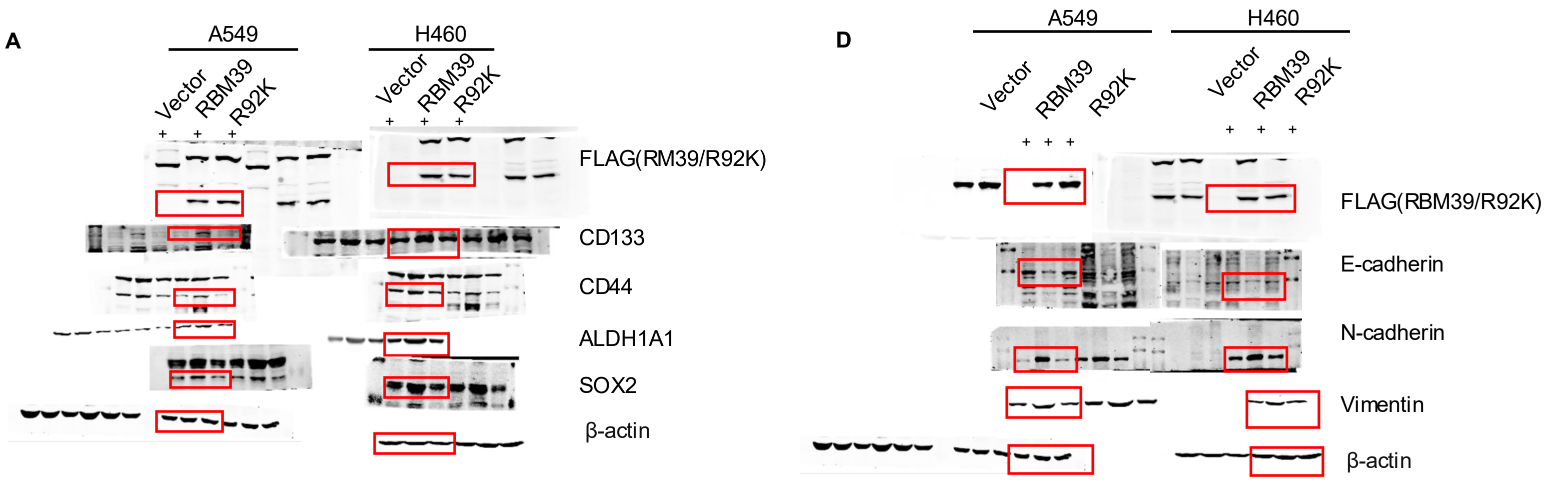
A
A549
H460
Vector
RBM39
R92K
+
FLAG(RM39/R92K)
CD133
CD44
ALDH1A1
SOX2
β-actin
D
FLAG(RBM39/R92K)
E-cadherin
N-cadherin
Vimentin

Figure7

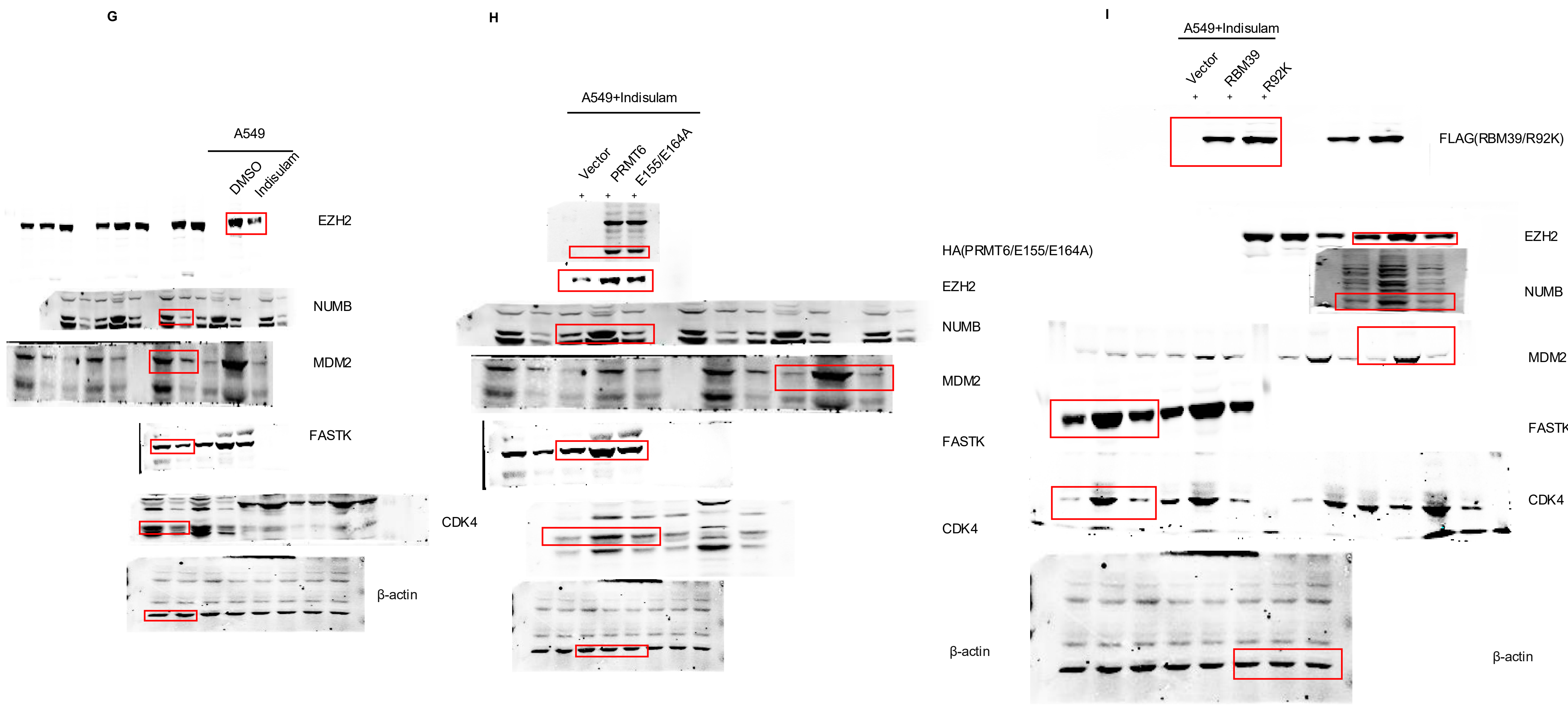
G
A549
DMSO
Indisulam
EZH2
NUMB
MDM2
FASTK
CDK4
β-actin
H
A549+Indisulam
Vector
PRMT6
E155/E164A
+
+
+
HA(PRMT6/E155/E164A)
EZH2
NUMB
MDM2
FASTK
CDK4
β-actin
I
A549+Indisulam
Vector
RBM39
R92K
+
+
+
FLAG(RBM39/R92K)
EZH2
NUMB
MDM2
FASTK
CDK4
β-actin

Figure6

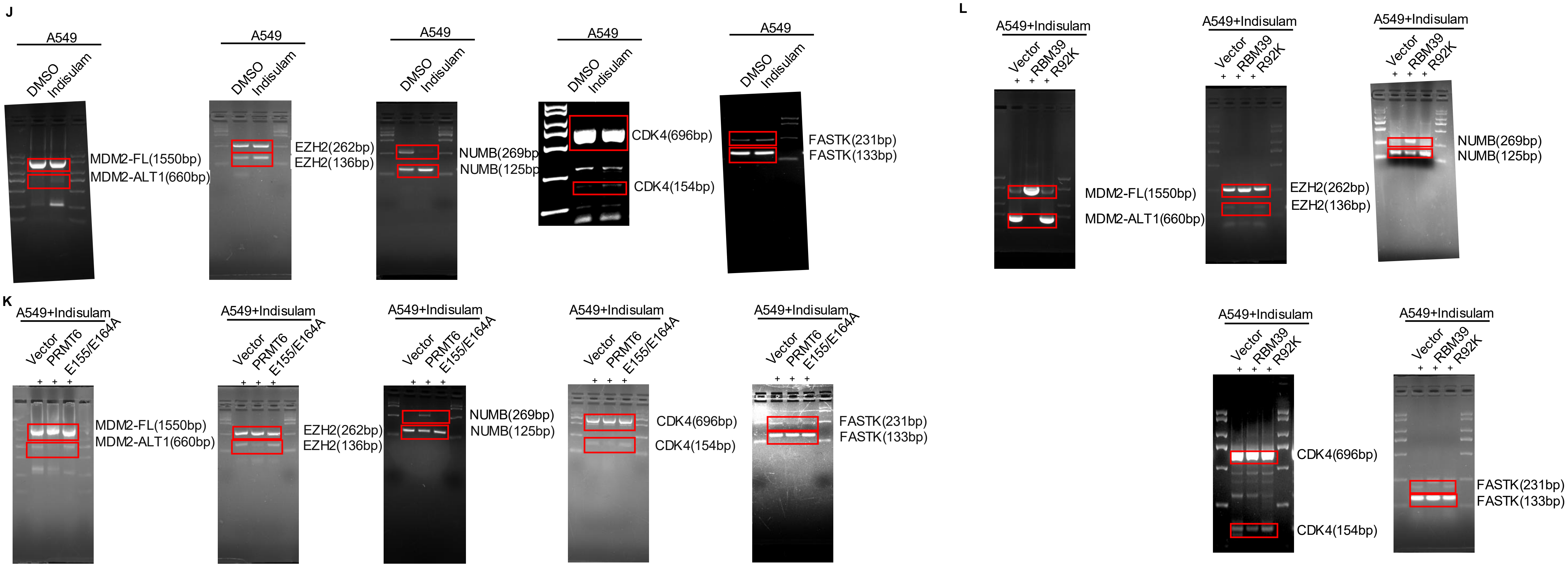
J
A549
DMSO
Indisulam
MDM2-FL(1550bp)
MDM2-ALT1(660bp)
A549
DMSO
Indisulam
EZH2(262bp)
EZH2(136bp)
A549
DMSO
Indisulam
NUMB(269bp
NUMB(125bp
A549
DMSO
Indisulam
CDK4(696bp)
CDK4(154bp)
A549
DMSO
Indisulam
FASTK(231bp)
FASTK(133bp)
L
A549+Indisulam
Vector
RBM39
R92K
MDM2-FL(1550bp)
MDM2-ALT1(660bp)
A549+Indisulam
Vector
RBM39
R92K
EZH2(262bp)
EZH2(136bp)
A549+Indisulam
Vector
RBM39
R92K
NUMB(269bp)
NUMB(125bp)
K
A549+Indisulam
Vector
PRMT6
E155/E164A
MDM2-FL(1550bp)
MDM2-ALT1(660bp)
A549+Indisulam
Vector
PRMT6
E155/E164A
EZH2(262bp)
EZH2(136bp)
A549+Indisulam
Vector
PRMT6
E155/E164A
NUMB(269bp)
NUMB(125bp)
A549+Indisulam
Vector
PRMT6
E155/E164A
CDK4(696bp)
CDK4(154bp)
A549+Indisulam
Vector
PRMT6
E155/E164A
FASTK(231bp)
FASTK(133bp)
A549+Indisulam
Vector
RBM39
R92K
CDK4(696bp)
CDK4(154bp)
A549+Indisulam
Vector
RBM39
R92K
FASTK(231bp)
FASTK(133bp)

Figure8

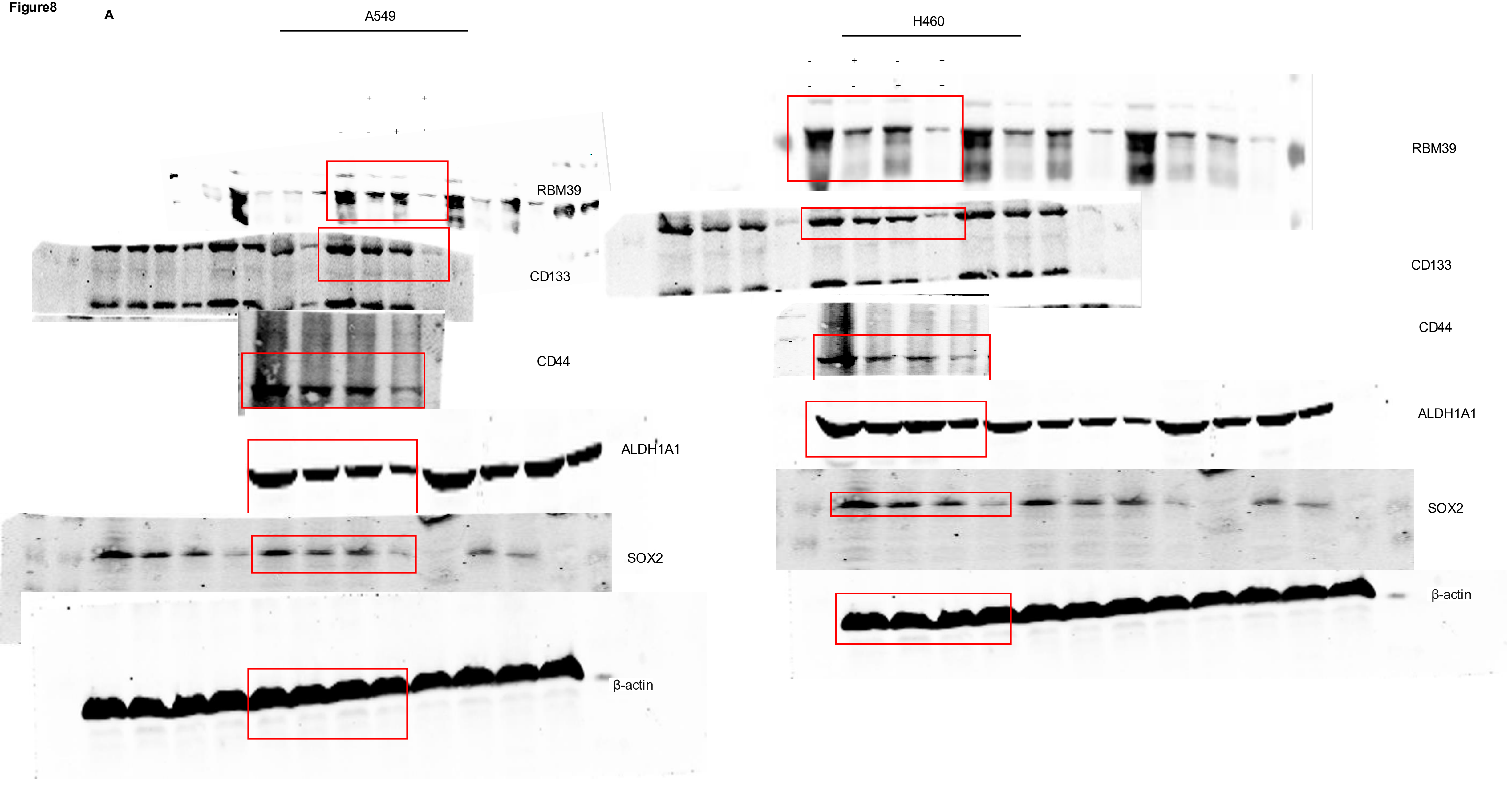
A
A549
H460
RBM39
CD133
CD44
ALDH1A1
SOX2
β-actin
RBM39
CD133
CD44
ALDH1A1
SOX2
β-actin

Figure8

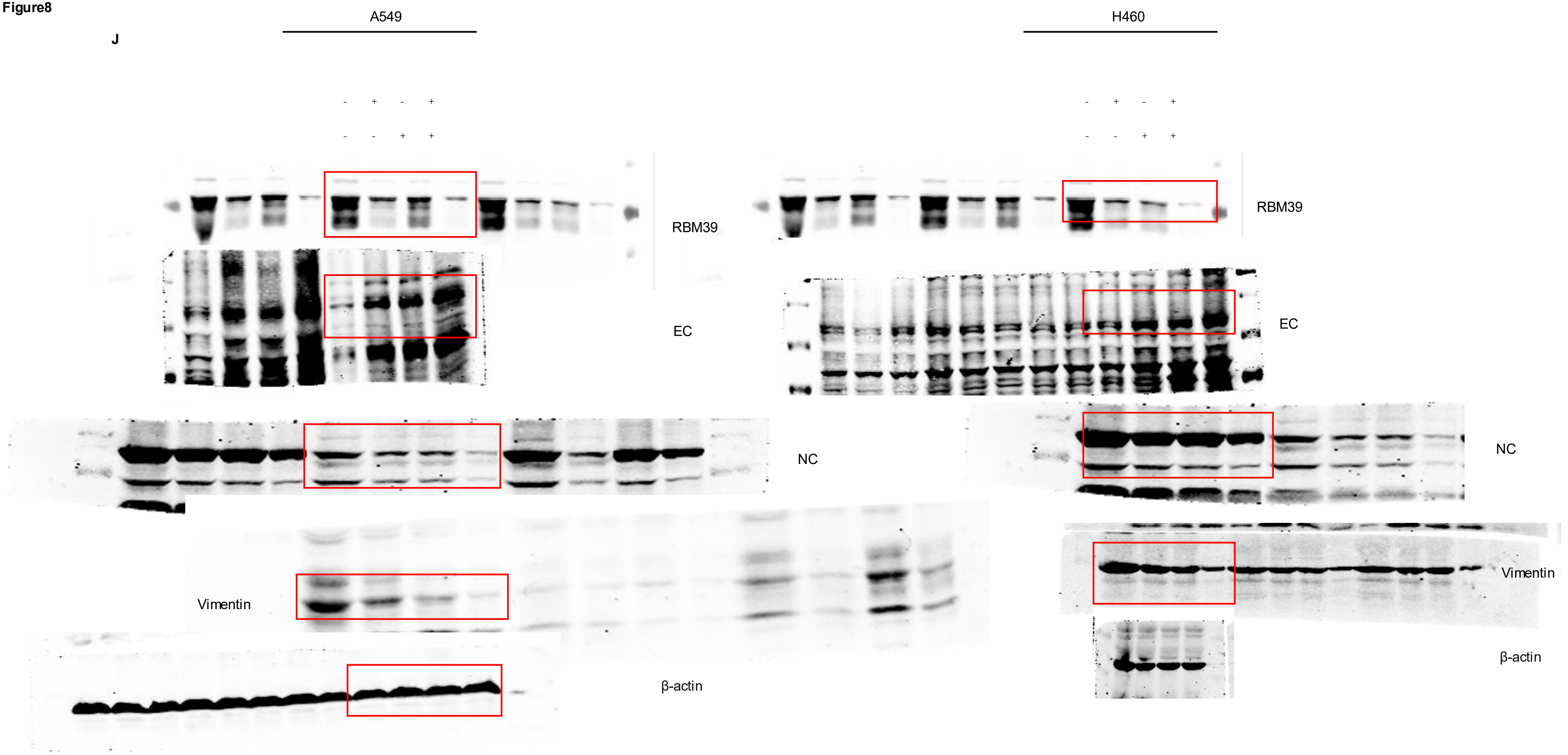
J
A549
H460
- + - +
- - + +
- + - +
- - + +
RBM39
RBM39
EC
EC
NC
NC
Vimentin
Vimentin
β-actin
β-actin

FigureS2

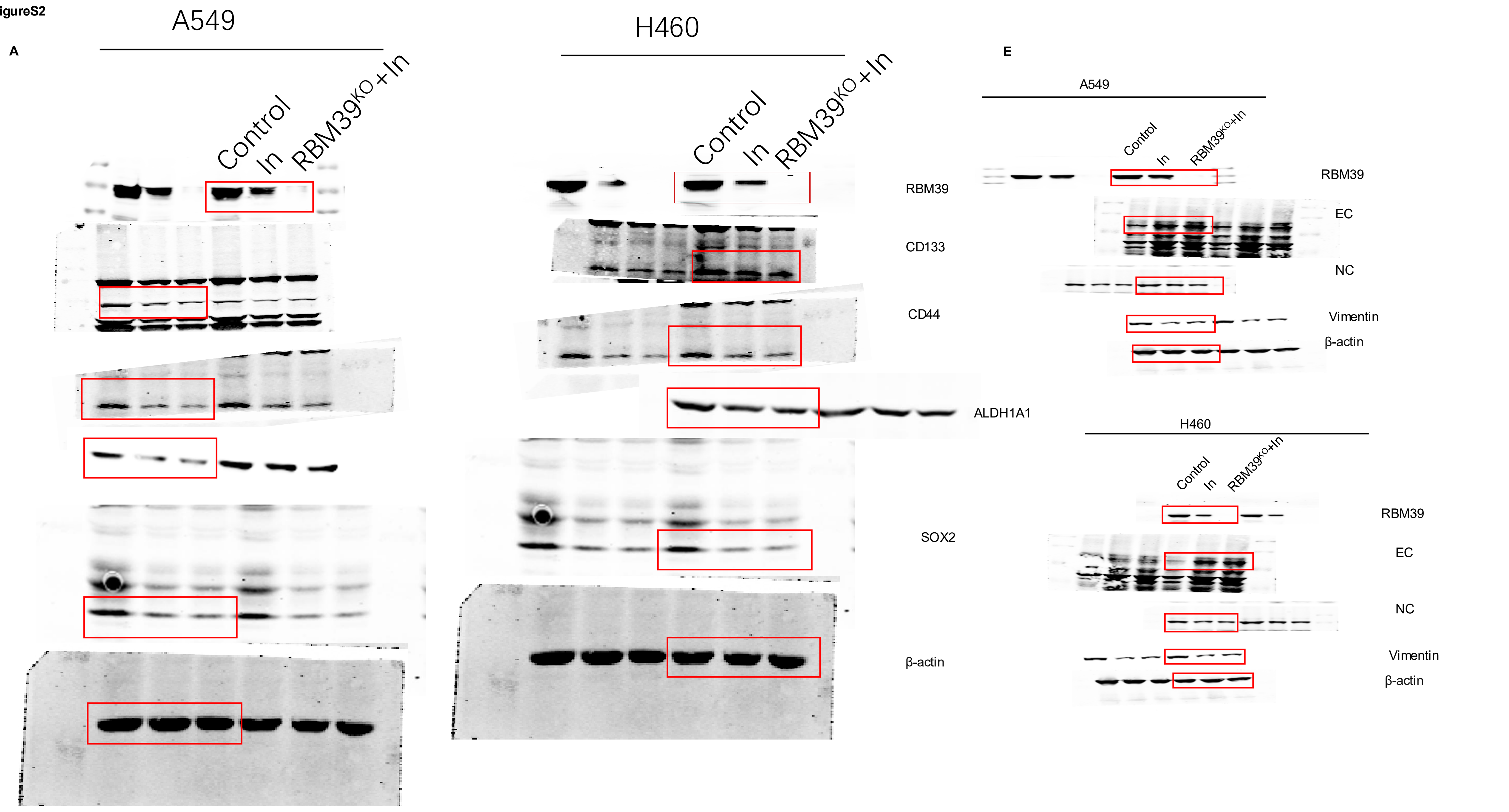
A
A549
Control
In
RBM39KO+In
H460
Control
In
RBM39KO+In
RBM39
CD133
CD44
ALDH1A1
SOX2
β-actin
E
A549
Control
In
RBM39KO+In
RBM39
EC
NC
Vimentin
β-actin
H460
Control
In
RBM39KO+In
RBM39
EC
NC
Vimentin
β-actin

FigureS3

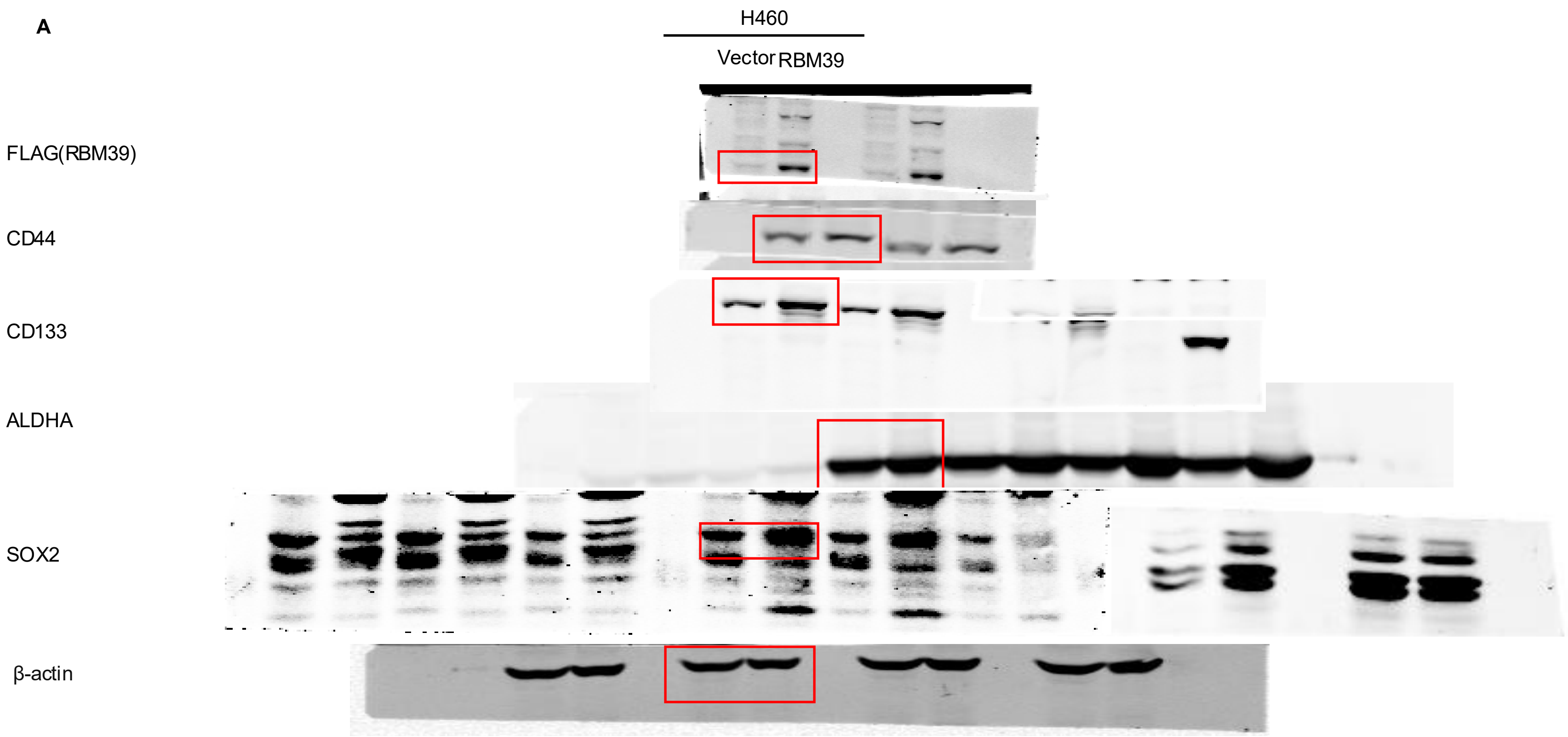
A
H460
Vector RBM39
FLAG(RBM39)
CD44
CD133
ALDHA
SOX2
β-actin

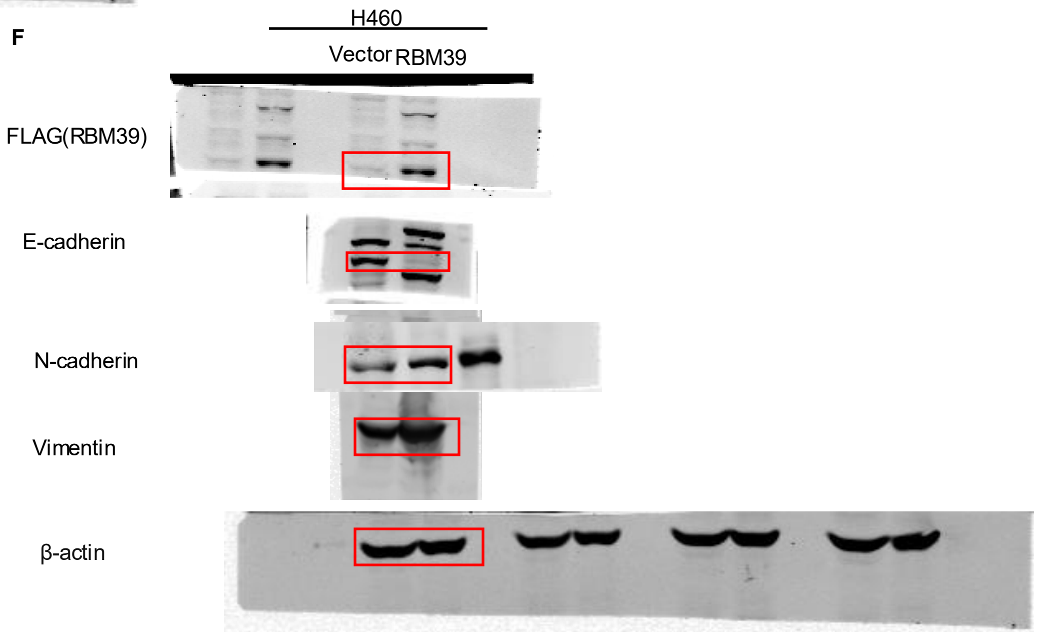
F
H460
Vector RBM39
FLAG(RBM39)
E-cadherin
N-cadherin
Vimentin
β-actin

FigureS3

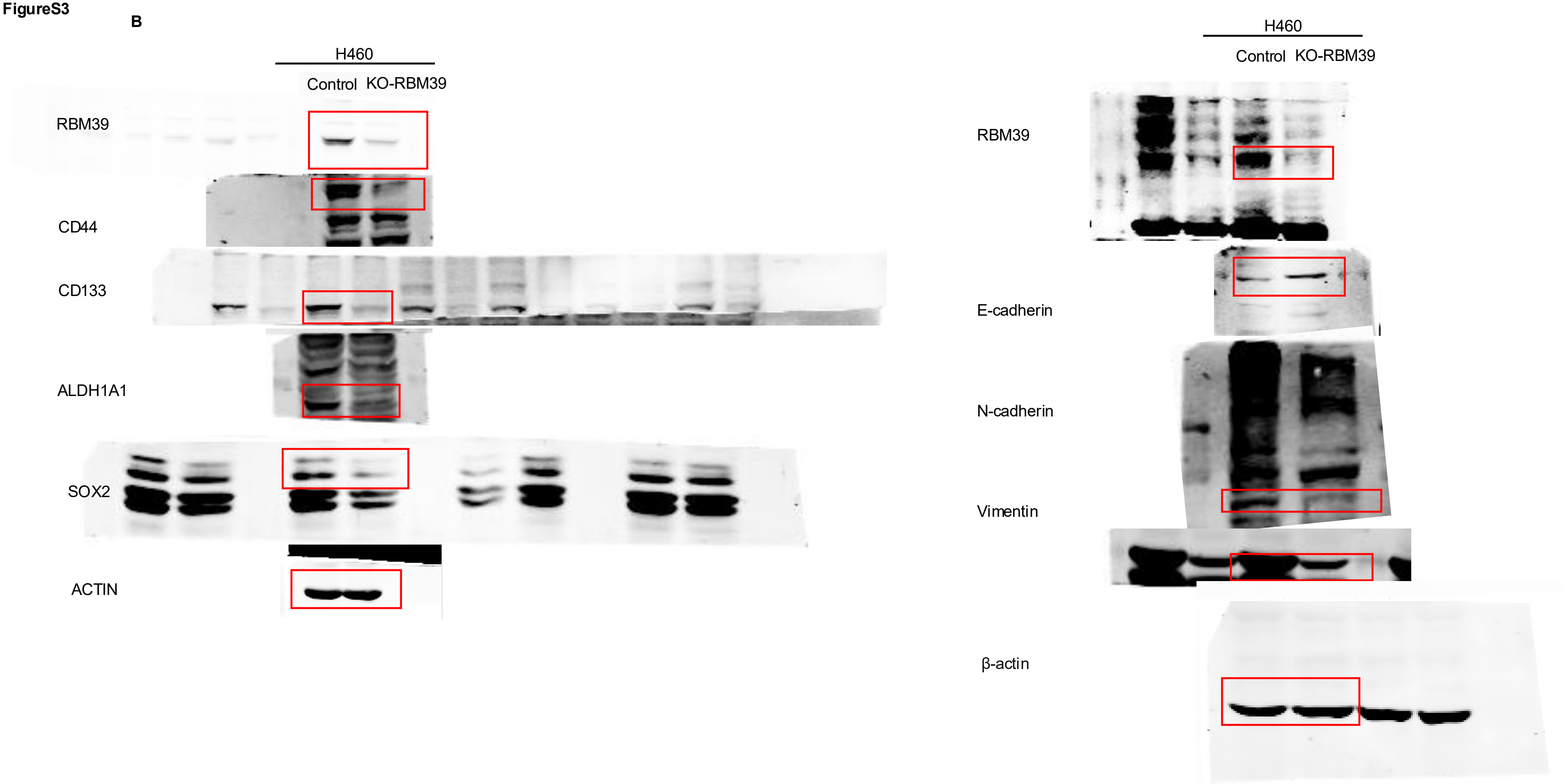
B
H460
Control
KO-RBM39
RBM39
CD44
CD133
ALDH1A1
SOX2
ACTIN
H460
Control
KO-RBM39
RBM39
E-cadherin
N-cadherin
Vimentin
β-actin

FigureS4

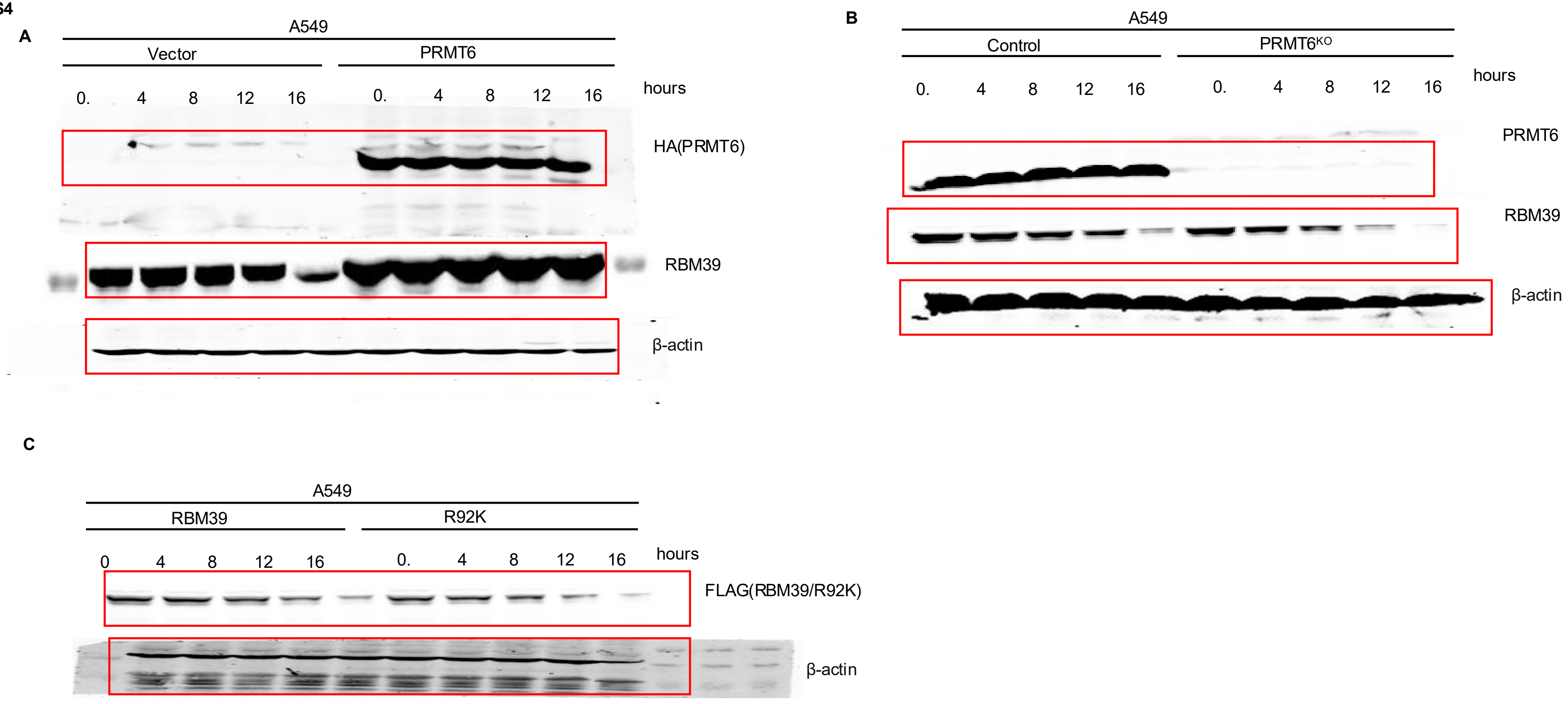
A
A549
Vector
PRMT6
0. 4 8 12 16 0. 4 8 12 16
hours
HA(PRMT6)
RBM39
β-actin
B
A549
Control
PRMT6KO
0. 4 8 12 16 0. 4 8 12 16
hours
PRMT6
RBM39
β-actin
C
A549
RBM39
R92K
0 4 8 12 16 0. 4 8 12 16
hours
FLAG(RBM39/R92K)
β-actin

FigureS5

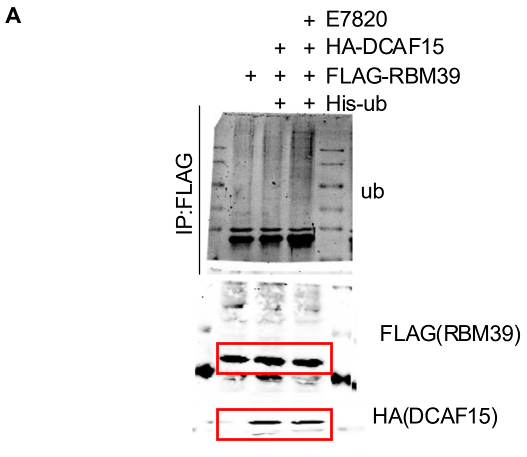
A
+ E7820
+ + HA-DCAF15
+ + + FLAG-RBM39
+ + His-ub
IP:FLAG
ub
FLAG(RBM39)
HA(DCAF15)

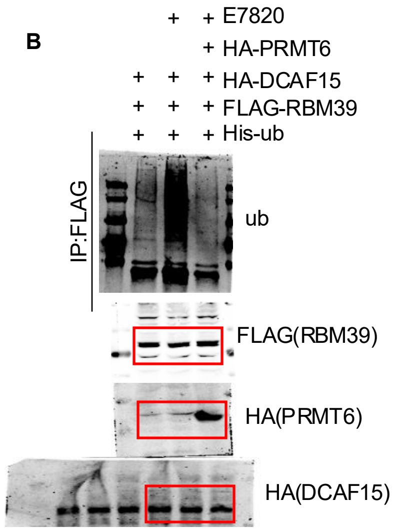
B
+ + E7820
+ HA-PRMT6
+ + + HA-DCAF15
+ + + FLAG-RBM39
+ + + His-ub
IP:FLAG
ub
FLAG(RBM39)
HA(PRMT6)
HA(DCAF15)

FigureS6

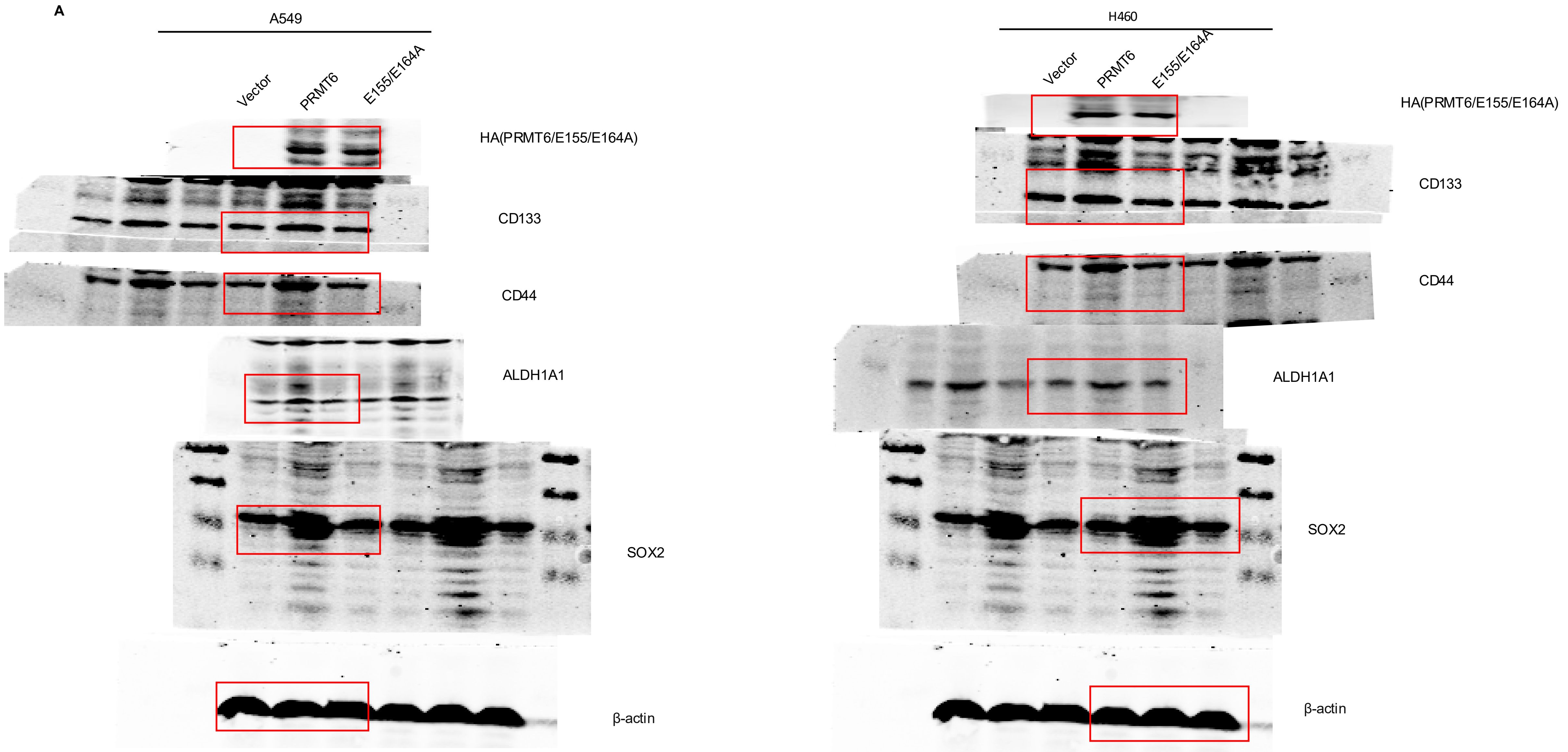
A
A549
Vector
PRMT6
E155/E164A
HA(PRMT6/E155/E164A)
CD133
CD44
ALDH1A1
SOX2
β-actin
H460
Vector
PRMT6
E155/E164A
HA(PRMT6/E155/E164A)
CD133
CD44
ALDH1A1
SOX2
β-actin

FigureS6

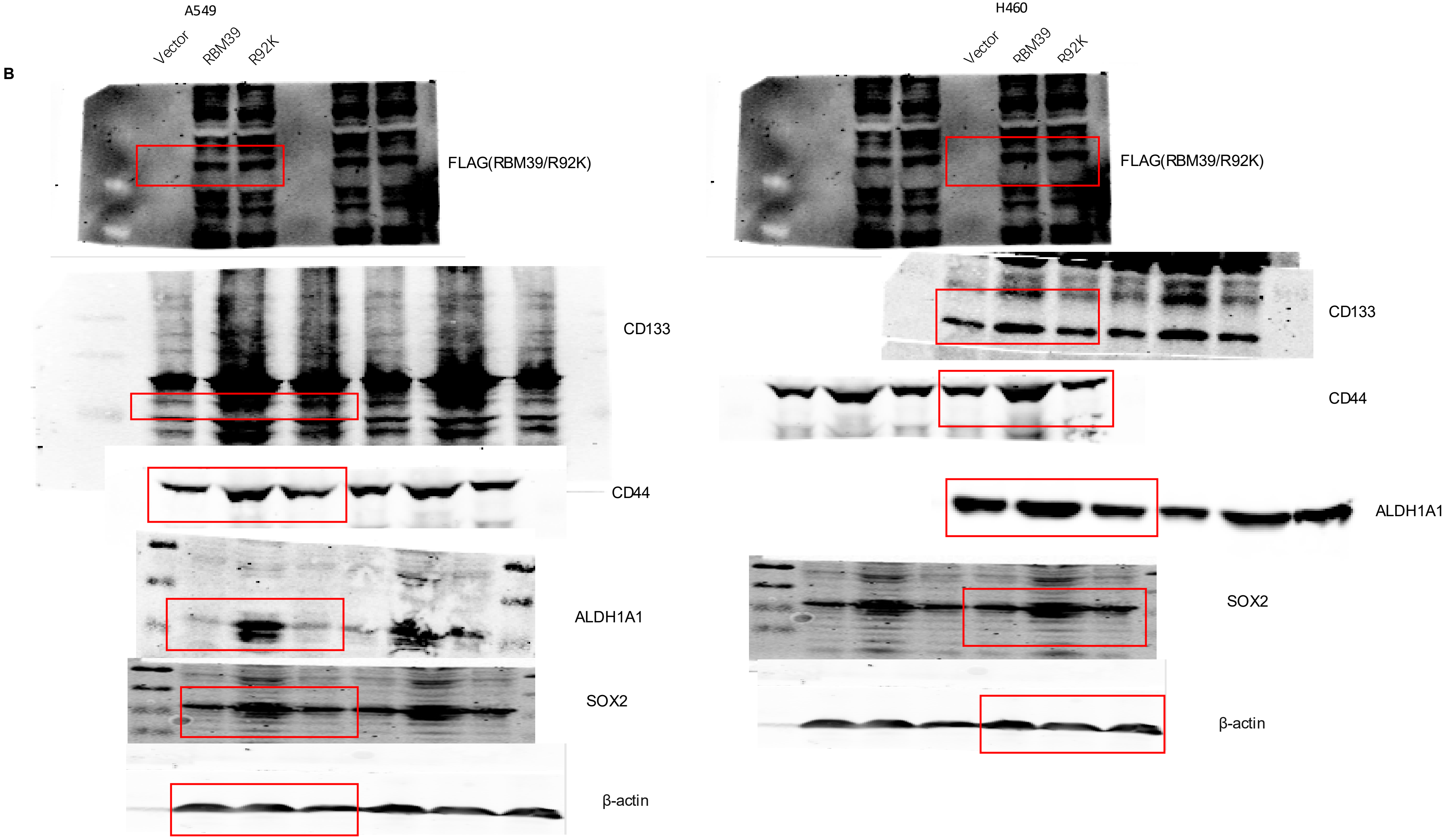
A549
Vector
RBM39
R92K
B
FLAG(RBM39/R92K)
CD133
CD44
ALDH1A1
SOX2
β-actin
H460
Vector
RBM39
R92K
FLAG(RBM39/R92K)
CD133
CD44
ALDH1A1
SOX2
β-actin

FigureS6

G

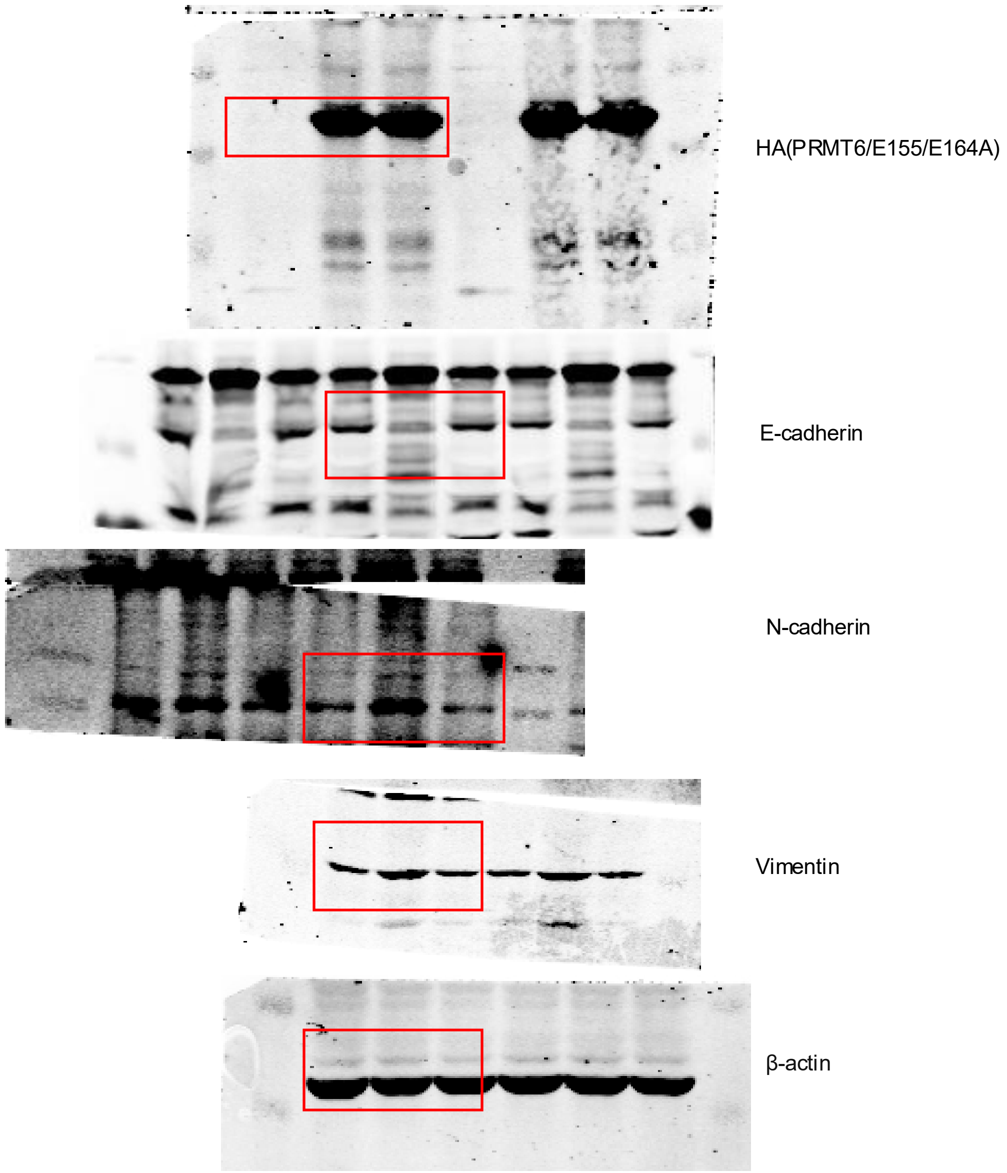

HA(PRMT6/E155/E164A)
E-cadherin
N-cadherin
Vimentin
β-actin

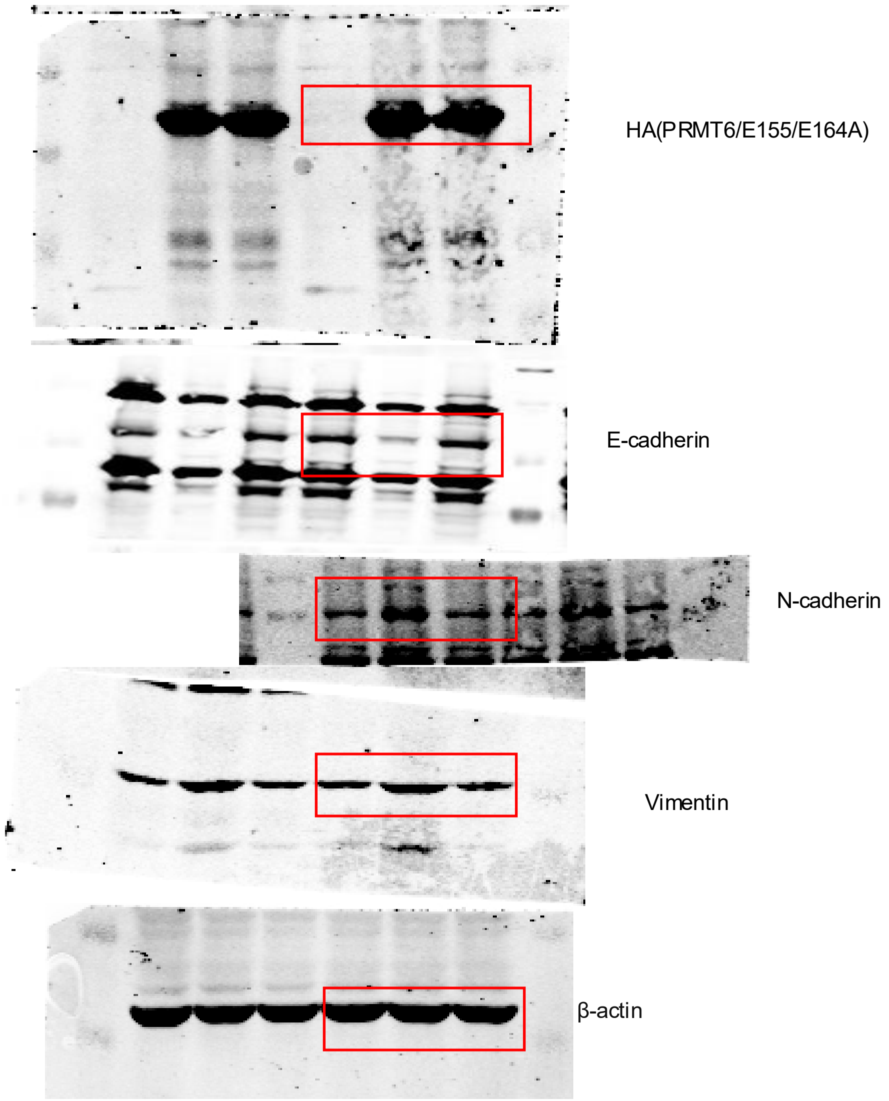

HA(PRMT6/E155/E164A)
E-cadherin
N-cadherin
Vimentin
β-actin

FigureS6

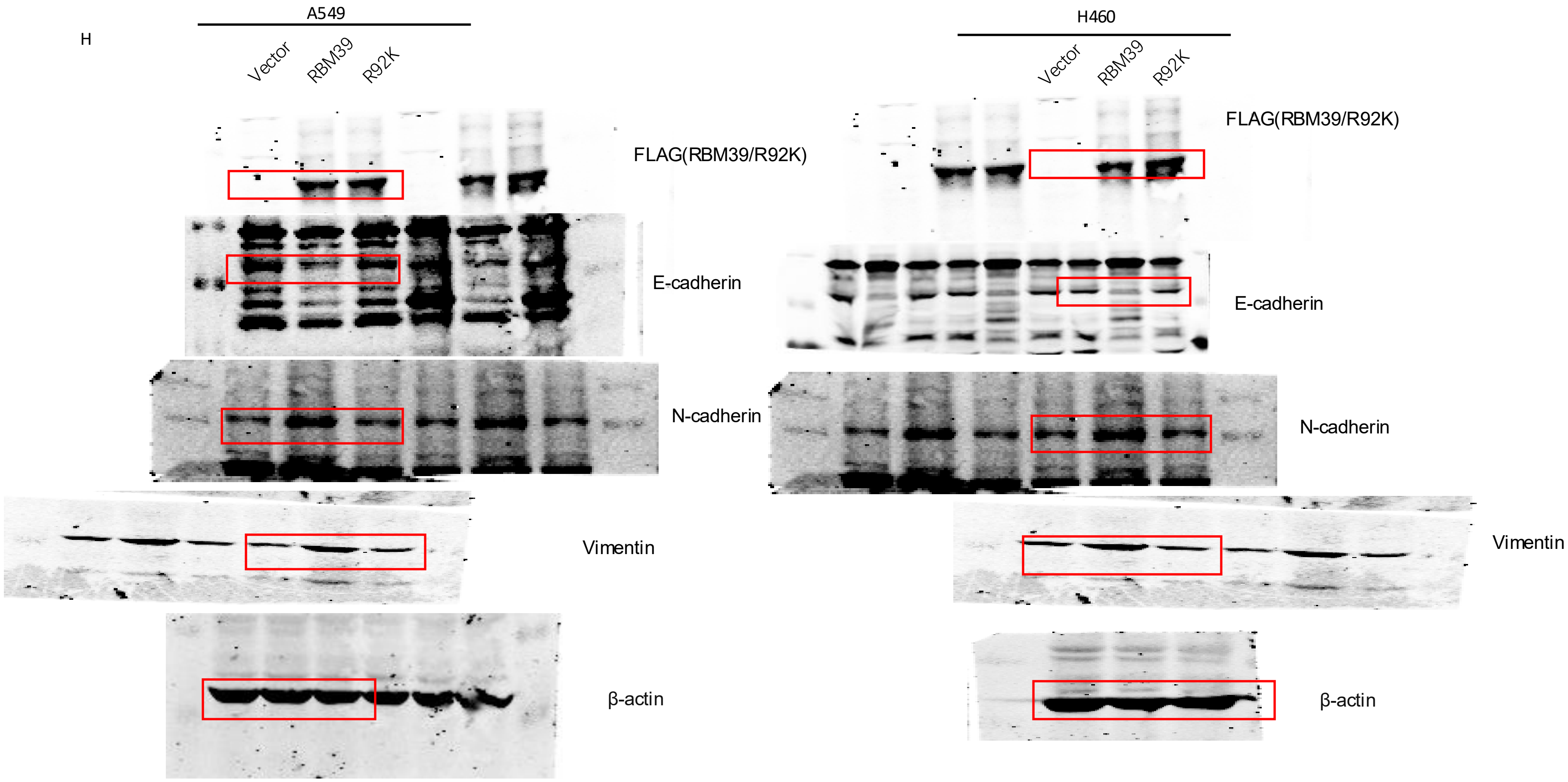
H
A549
Vector
RBM39
R92K
H460
Vector
RBM39
R92K
FLAG(RBM39/R92K)
E-cadherin
N-cadherin
Vimentin
β-actin
FLAG(RBM39/R92K)
E-cadherin
N-cadherin
Vimentin
β-actin

FigureS7

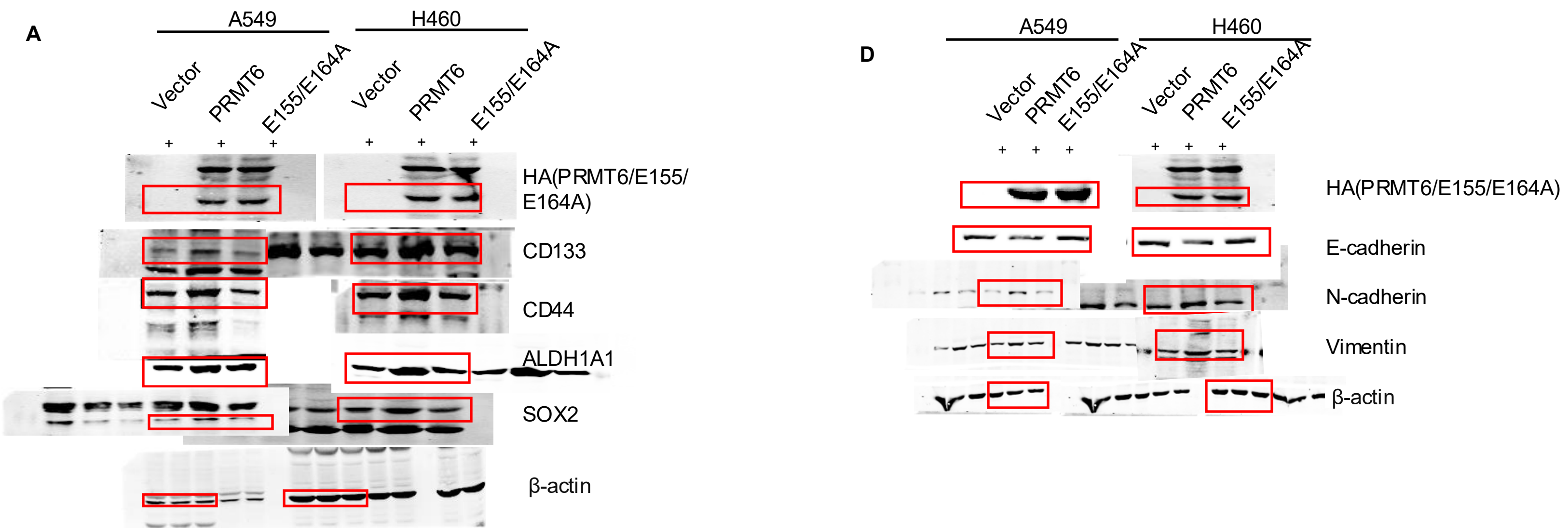
A
A549
H460
Vector
PRMT6
E155/E164A
Vector
PRMT6
E155/E164A
HA(PRMT6/E155/E164A)
CD133
CD44
ALDH1A1
SOX2
β-actin
D
A549
H460
Vector
PRMT6
E155/E164A
Vector
PRMT6
E155/E164A
HA(PRMT6/E155/E164A)
E-cadherin
N-cadherin
Vimentin
β-actin

FigureS8

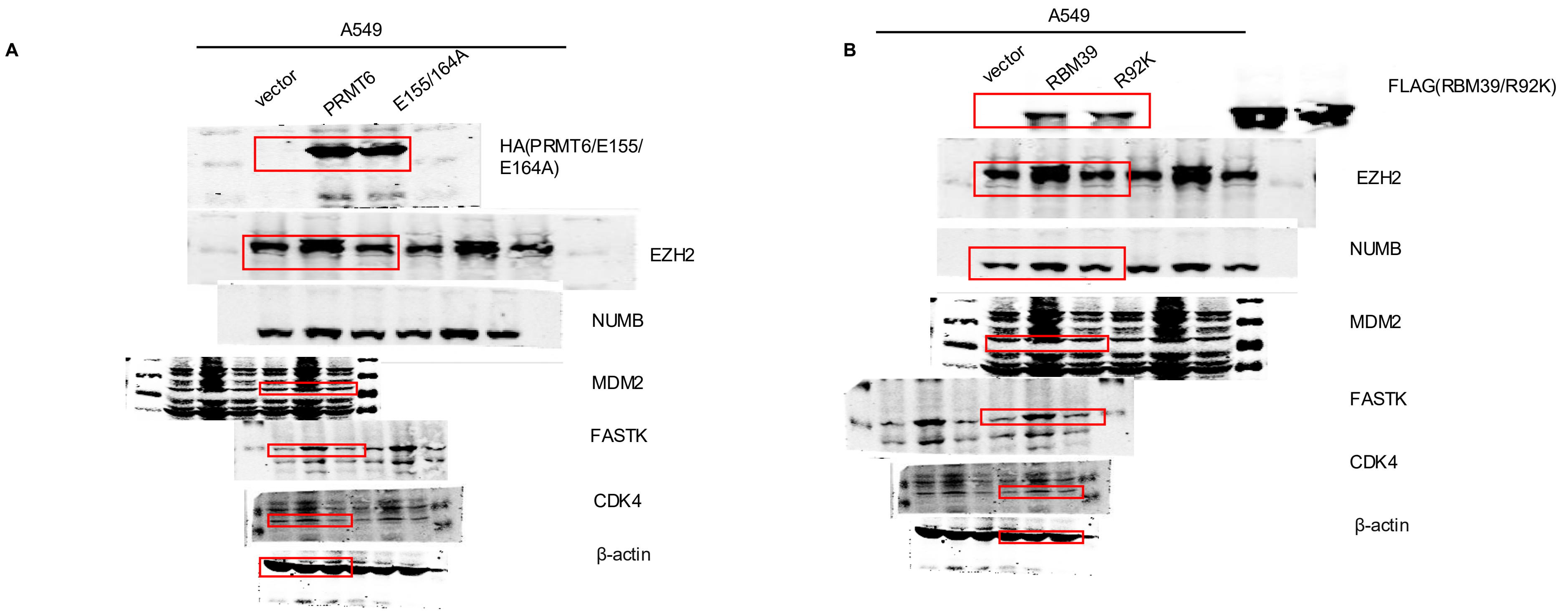
A
A549
vector
PRMT6
E155/164A
HA(PRMT6/E155/
E164A)
EZH2
NUMB
MDM2
FASTK
CDK4
β-actin
B
A549
vector
RBM39
R92K
FLAG(RBM39/R92K)
EZH2
NUMB
MDM2
FASTK
CDK4
β-actin

FigureS8

C

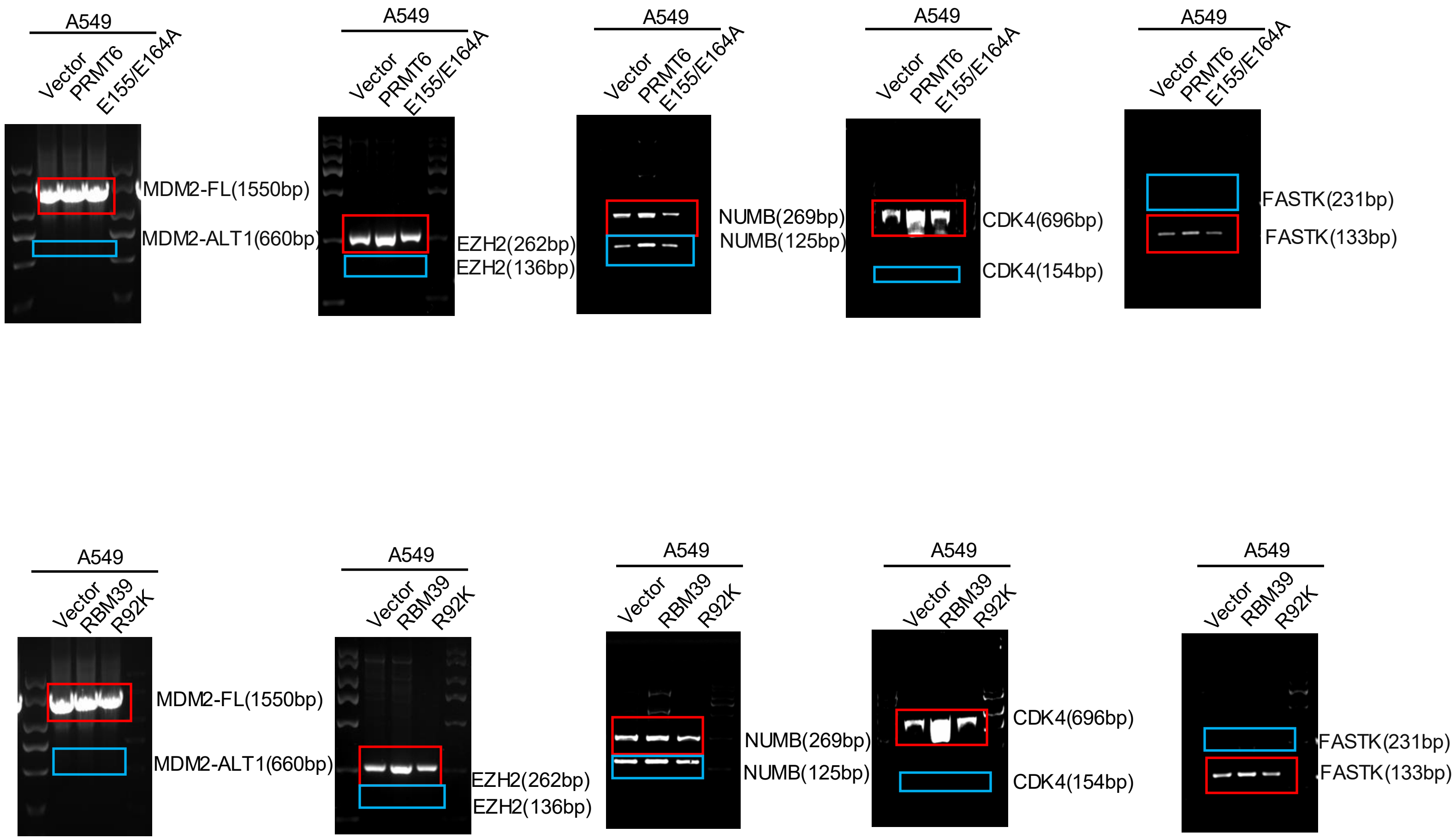
A549
Vector
PRMT6
E155/E164A
MDM2-FL(1550bp)
MDM2-ALT1(660bp)
EZH2(262bp)
EZH2(136bp)
NUMB(269bp)
NUMB(125bp)
CDK4(696bp)
CDK4(154bp)
FASTK(231bp)
FASTK(133bp)
RBM39
R92K

FigureS9

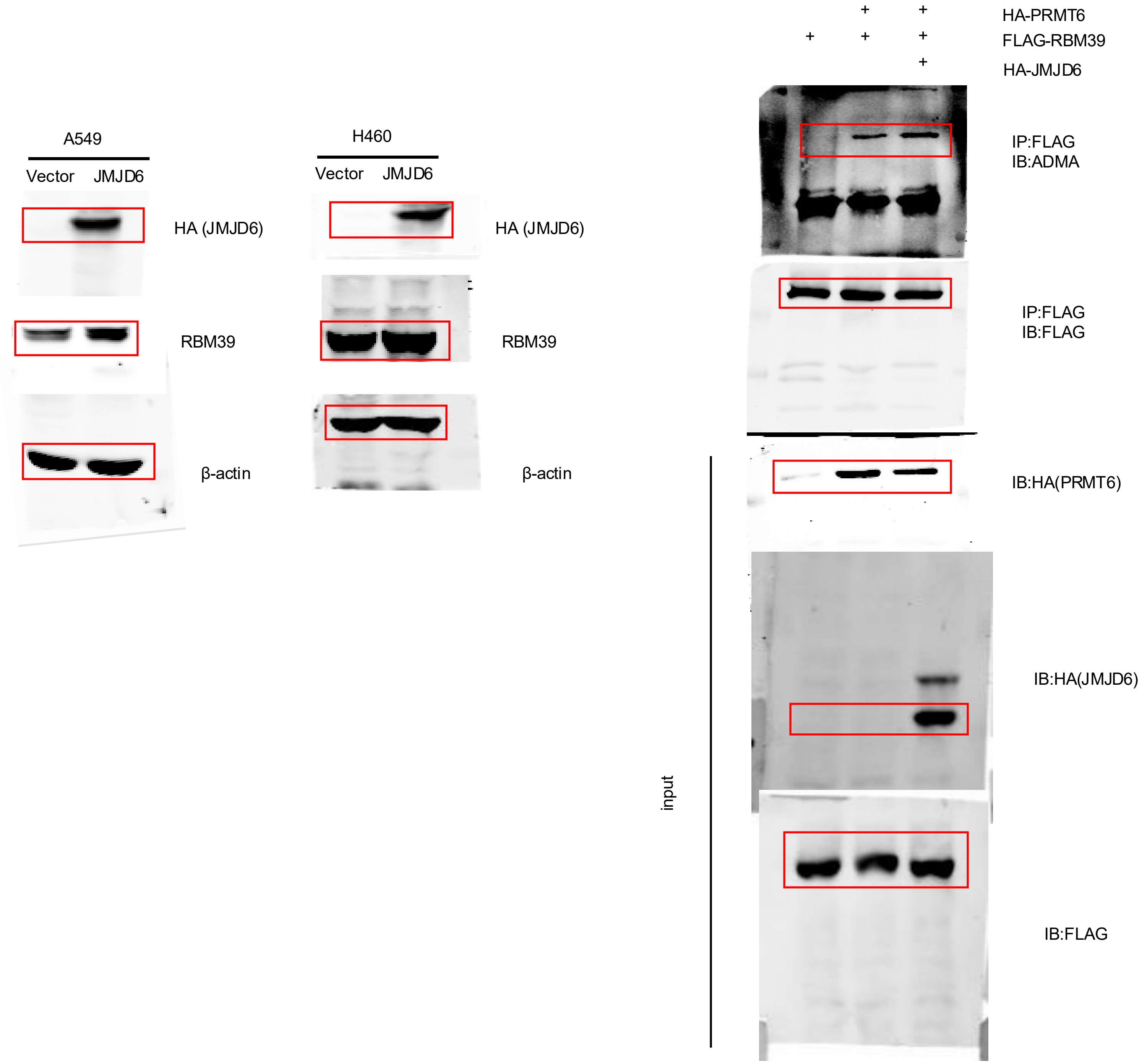
A549
Vector
JMJD6
HA (JMJD6)
RBM39
β-actin
H460
Vector
JMJD6
HA (JMJD6)
RBM39
β-actin
HA-PRMT6
FLAG-RBM39
HA-JMJD6
IP:FLAG
IB:ADMA
IP:FLAG
IB:FLAG
IB:HA(PRMT6)
IB:HA(JMJD6)
IB:FLAG
input
